# A deep learning-based method enables the automatic and accurate assembly of chromosome-level genomes

**DOI:** 10.1093/nar/gkae789

**Published:** 2024-09-17

**Authors:** Zijie Jiang, Zhixiang Peng, Zhaoyuan Wei, Jiahe Sun, Yongjiang Luo, Lingzi Bie, Guoqing Zhang, Yi Wang

**Affiliations:** Integrative Science Center of Germplasm Creation in Western China (CHONGQING) Science City, Biological Science Research Center, Southwest University, Chongqing, China; Integrative Science Center of Germplasm Creation in Western China (CHONGQING) Science City, Biological Science Research Center, Southwest University, Chongqing, China; Integrative Science Center of Germplasm Creation in Western China (CHONGQING) Science City, Biological Science Research Center, Southwest University, Chongqing, China; Integrative Science Center of Germplasm Creation in Western China (CHONGQING) Science City, Biological Science Research Center, Southwest University, Chongqing, China; Integrative Science Center of Germplasm Creation in Western China (CHONGQING) Science City, Biological Science Research Center, Southwest University, Chongqing, China; Integrative Science Center of Germplasm Creation in Western China (CHONGQING) Science City, Biological Science Research Center, Southwest University, Chongqing, China; Integrative Science Center of Germplasm Creation in Western China (CHONGQING) Science City, Biological Science Research Center, Southwest University, Chongqing, China; Integrative Science Center of Germplasm Creation in Western China (CHONGQING) Science City, Biological Science Research Center, Southwest University, Chongqing, China

## Abstract

The application of high-throughput chromosome conformation capture (Hi-C) technology enables the construction of chromosome-level assemblies. However, the correction of errors and the anchoring of sequences to chromosomes in the assembly remain significant challenges. In this study, we developed a deep learning-based method, AutoHiC, to address the challenges in chromosome-level genome assembly by enhancing contiguity and accuracy. Conventional Hi-C-aided scaffolding often requires manual refinement, but AutoHiC instead utilizes Hi-C data for automated workflows and iterative error correction. When trained on data from 300+ species, AutoHiC demonstrated a robust average error detection accuracy exceeding 90%. The benchmarking results confirmed its significant impact on genome contiguity and error correction. The innovative approach and comprehensive results of AutoHiC constitute a breakthrough in automated error detection, promising more accurate genome assemblies for advancing genomics research.

## Graphical Abstract

**Graphical Abstract:**
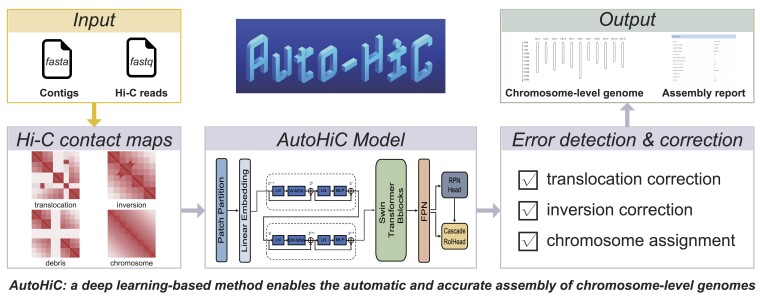

## Introduction

Genomics is crucial for understanding gene function and evolutionary relationships among species. Chromosome-level genome sequences information is essential for scientific progress in these areas (1). This objective is critical as it provides the foundation for uncovering the principles of biological systems and investigating the molecular mechanisms underlying disease.

Recent advances in genome assembly have been propelled by the emergence of long-read sequencing technologies (2,3), such as PacBio’s Single Molecule Real-Time (SMRT) (4) sequencing and Oxford Nanopore Technologies (ONT) (5,6). These technological advancements have overcome the limitations of traditional next-generation sequencing (NGS) methods (7) by providing read lengths that exceed conventional boundaries. These factors have resulted in a significant increase in comprehensive and contiguous genome assemblies. Nonetheless, despite these advancements, achieving chromosome-level assembly remains a challenging goal due to the inability of current read lengths to span entire chromosomes.

In the process of genome assembly, high-throughput chromosome conformation capture (Hi-C) sequencing has emerged as an indispensable tool. This technique, which combines proximity ligation with sequencing, seeks to scaffold contigs into chromosome-level assemblies by leveraging the increased density of Hi-C linkage pairs among adjacent contigs (8–13). Numerous tools, including Lachesis (14), 3D-DNA (15), SALSA (16,17), YaHS (18), instaGRAAL (19), EndHiC (20), AllHiC (21) and Pin_hic (22), have been developed to scaffold Hi-C data into chromosome-level scaffolds. However, each of these tools has its limitations and is subject to various biases. Some require prespecification of the chromosome number, which poses a challenge for users. Additionally, errors in the output from these tools often necessitate manual correction, prolonging the process and introducing the potential for human error. This reliance on manual intervention hinders the realization of fully automated genome assembly, particularly when aiming for chromosome-level accuracy with Hi-C data.

In addition to major genomic research initiatives such as the Bird 10000 Genomes (B10K) Project (23), the Earth Biogenome Project (EBP) (24), the i5K initiative (25) and the Vertebrate Genomes Project (VGP) (26), there is an urgent need for the automation of high-quality chromosome-level genome assembly at scale. However, traditional assembly methods struggle with the growing size and complexity of datasets and face challenges related to algorithmic accuracy and the availability of human resources. Deep learning has become increasingly integral to the life sciences (27–29), making significant contributions to data analysis and processing. The Transformer (30) architecture, an attention-based model designed for handling long sequences, has achieved notable success in language processing and has shown versatility in various fields, including image analysis, gene expression and protein folding. Despite the introduction of software such as DeepC (31), EagleC (32), VEHiCLE (33), DeepLoop (34), SnapHiC-D (35) and hicGAN (36), the potential of Hi-C data for identifying assembly errors is still largely untapped. Large datasets offer an opportunity for deep learning to effectively harness Hi-C data.

Here, we introduce AutoHiC, a scalable and computationally efficient method for deep learning-based error correction in Hi-C assembly. By utilizing genome-wide chromatin contact data from over half a million Hi-C images across approximately 300 species, AutoHiC automates the correction of assembly errors, enhancing both the contiguity and accuracy of genome assemblies. The efficacy of the AutoHiC recognition and error correction algorithm is demonstrated through a comparative analysis of pre- and post-adjustment contact heatmaps. Our findings revealed that AutoHiC surpasses other software for improving genome contiguity. The accuracy of the AutoHiC adjustments was confirmed by comparing the adjusted genome sequences with the telomere-to-telomere (T2T) reference genomes, which showed a high degree of consistency with the T2T genome sequence. Furthermore, to ascertain the broad applicability of AutoHiC, we conducted tests across various species, including those with large genomes, numerous chromosomes and polyploidy. The results confirmed that AutoHiC is applicable and effective in diverse genomic assembly scenarios.

In conclusion, AutoHiC uses deep learning and Hi-C data to automate chromosome-level genome assembly and improve scaffolding accuracy. It detects and corrects errors in Hi-C assemblies, complementing manual curation efforts and streamlining the assembly of chromosome-level genomes. We hope that AutoHiC will advance genomics research and deepen our understanding of three-dimensional genome structure and functionality.

## Materials and methods

### AutoHiC workflow

AutoHiC consists of three main steps. To begin the process, users must prepare preexisting contig data, Hi-C sequencing data and information about the restriction enzymes used in the Hi-C experimental setup.

The first step was to use Juicer to align the Hi-C reads to the contig genome. An intermediary file called merged_nodups.txt is subsequently generated. This file contains a list of paired alignments from an *in situ* Hi-C experiment, with duplicates removed. This intermediate file is utilized by 3D-DNA (version 180922) to enhance the assembly of contigs into scaffolds. This phase produces several outputs, including the hic, assembly and scaffold files. These files are crucial inputs for the error detection and correction module of AutoHiC.

In the second stage, AutoHiC can utilize the error detection module to identify the location of errors in the hic file output by 3D-DNA and record the corresponding information. The error correction module can then adjust the errors based on the detected error and sequence information. The identification and correction processes are iterative and the number of iterations depends on the user’s settings in the configuration file. The default is to continue iterating until all the error adjustments are completed. The chromosome assignment module accurately identifies chromosomal regions and delineates their boundaries according to the refined outcomes. In the final stage, all the relevant assembly information is synthesized holistically. The genome was then statistically analyzed and a comprehensive report of the results was generated.

Notably, AutoHiC can directly call 3D-DNA, which is currently the most widely used scaffolder. However, users operating different assembly software could convert the outcomes into hic formats to use AutoHiC for assembly correction and related tasks.

### Error detection module

AutoHiC’s error detection module utilizes a deep learning detection model to identify translocations, inversions, debris and chromosomes on Hi-C contact heatmap images. By learning many translocations, inversions, debris and chromosome images, the model can accurately identify these objects in the image and their positions. The position of the error in the image can be mapped to the genome sequence by combining the corresponding sequence region information.

AutoHiC first converts the contact matrix in Hi-C format into contact images. The Hi-C format file is a highly compressed binary file that stores contact matrices at multiple resolutions (usually 500 bp to 1.25 Mb). To extract the contact matrix (${M}_i$) based on the hic and assembly files in the 3D-DNA output result, we first utilize hicstraw (v1.3.1, parameters: ${\rm resolutions}$ = getResolutions(); getMatrixZoomData(‘assembly’, ‘assembly’, ‘observed’, ‘NONE’, ‘BP’, ${\rm resolution}$); ${M}_i$ = getRecordsAsMatrix(${X}_i$, ${Y}_i$, ${X}_i$,${\mathrm{\ }}{Y}_i$)) and sliding window algorithms.


\begin{eqnarray*}\left\{ {\begin{array}{@{}*{1}{l}@{}} {{X}_i = {\rm resolution}*400\left( {i - 1} \right)\qquad \left( {0 \le {X}_i < {L}_g} \right)}\\ {{Y}_i = {X}_i + {\rm resolution}*700\qquad\quad \left( {0 \le {Y}_i \le {L}_g} \right)}\\\qquad\qquad\qquad\qquad {i \le}\left\lfloor {\frac{{{L}_g}}{{{Y}_i}}}\right\rfloor \end{array}} \right.\end{eqnarray*}

where ${L}_g$ represents the full length of the genome in the hic file. ${\rm resolution}$ iterates through each value in the ${\rm resolutions}$ array. ${X}_i$ denotes the starting position for obtaining the contact matrix, while ${Y}_i$ denotes the ending position. During the extraction process, a specific window length and step size are used to perform the extraction along the diagonal line of the entire contact map $({\rm windows}{\mathrm{\ }}{\rm length }= {\rm resolution}{\mathrm{*}}700;{\rm step}{\mathrm{\ }}{\rm size }= {\rm resolution}{\mathrm{*}}400$). The contact matrix for the local region is extracted and visualized as an image using the matplotlib (37) (v3.7.1) function pyplot in Python. AutoHiC records the corresponding genome region and resolution for each image during this process.

AutoHiC inputs each generated contact image into the object detection model. The model scans the entire image to identify translocations, inversions or debris. If any of these are present, their location ($X,{\mathrm{\ }}Y,{\mathrm{\ }}W,{\mathrm{\ }}H$) on the image is recorded. These locations contain incorrect breakpoints. The position of the error in the genome can be calculated by integrating the position information recorded in the image with the information recorded in the assembly file through ratio calculations (Supplementary Figure S8). The formulas for converting the positions are as follows:


\begin{eqnarray*}{E}_{\left( {s,{\mathrm{\ }}x} \right)} = X{\mathrm{*}}\frac{{{L}_{\left( {e,x} \right)} - {L}_{\left( {s,x} \right)}}}{{{I}_w}} + {L}_{\left( {s,x} \right)}\end{eqnarray*}

\begin{eqnarray*}{E}_{\left( {e,x} \right)} = W{\mathrm{*}}\frac{{{L}_{\left( {e,x} \right)} - {L}_{\left( {s,x} \right)}}}{{{I}_w}} + {L}_{\left( {s,x} \right)}\end{eqnarray*}

\begin{eqnarray*}{E}_{\left( {s,y} \right)} = Y{\mathrm{*}}\frac{{{L}_{\left( {e,y} \right)} - {L}_{\left( {s,y} \right)}}}{{{I}_h}} + {L}_{\left( {s,y} \right)}\end{eqnarray*}

\begin{eqnarray*}{E}_{\left( {e,y} \right)} = H{\mathrm{*}}\frac{{{L}_{\left( {e,y} \right)} - {L}_{\left( {s,y} \right)}}}{{{I}_h}} + {L}_{\left( {s,y} \right)}\end{eqnarray*}

The starting and ending positions of the error on the *x*-axis and *y*-axis of the contact matrix are represented by ${E}_{( {s,{\mathrm{\ }}x} )},{\mathrm{\ }}{E}_{( {e,x} )}$ and ${E}_{( {s,y} )},{\mathrm{\ }}{E}_{( {e,y} )}$, respectively. $X$ and $Y$ represent the starting positions of the upper-left corner of the error box on the *x*- and *y*- axes, respectively. $W$ and $H$ represent the width and length of the error box, respectively. The start and end positions of the contact image on the genome are represented by ${L}_{( {s,x} )},{\mathrm{\ }}{L}_{( {e,x} )}$ and ${L}_{( {s,y} )},{\mathrm{\ }}{L}_{( {e,y} )}$, respectively. ${I}_h$ and ${I}_w$ are the width and height of the contact images, respectively. Furthermore, AutoHiC filters errors according to the length settings specified in the configuration file. Finally, the filtered error information is saved to the appropriate folder. AutoHiC saves data separately based on the error type and counts the number of each type of error.

The identification of errors may need to be repeated multiple times depending on the user’s choice, as there may be multiple hic results from the previous step. For instance, 3D-DNA allows users to specify the number of corrections and obtain multiple results from which to choose. When faced with multiple results, AutoHiC identifies errors in each and selects the one with the fewest translocation and inversion errors for correction.

### Error correction module

This module leverages information (generated by the detection module) about the locations of errors within the genome to rectify them. The primary categories of errors discussed include translocation and inversion.

First, the error correction algorithm cuts at the misjoin sites on both sides of the error to correct it. The implementation is based on the start and end position information of the error returned by the detection module. It then searches for the scaffold sequence where the error is located on the genome. The sequence is divided into two fragments according to the start and end sites of the error: the error sequence fragment and the fragment before the error. Appropriate strategies for different types of errors are adopted. For the correction of translocations, the peak algorithm is employed to identify the insertion site, which precedes the actual correction. By utilizing the start and end coordinates of the translocation in conjunction with the assembly data, the scaffold fragment is pinpointed to the specified interval and subsequently repositioned according to the peak algorithm’s calculation. Inversion errors are rectified by rotating the previously isolated inversion fragment to its correct orientation within the genomic sequence.

After AutoHiC corrects these errors, it will generate a new assembly file containing space-delimited text that encodes, in a concise manner, a set of instructions to be performed on the draft sequences including assigning them, changing their order and orientation and anchoring them into chromosomes. The script (run-asm-pipeline-post-review.sh) can then be used to create a corrected genome sequence file based on this new assembly file. When the scaffold assemblies are high-quality, a single iteration of AutoHiC correction is often enough to rectify all identified errors comprehensively. This iterative strategy significantly contributes to increasing the algorithm’s accuracy and overall reliability.

### Chromosome assignment module

The chromosome assignment module encompasses two principal phases: chromosomal region identification and boundary delineation.

First, hicstraw (v1.3.1, parameters: contact data = getMatrixZoomData(‘assembly’, ‘assembly’, ‘observed’, ‘NONE’, ‘BP’, ${\rm resolution}$); $X$ = getRecordsAsMatrix(0, ${L}_g$, 0,$\ {L}_g$); ${\rm resolutions}$ = getResolutions()) is used to extract the contact matrix of the entire genome based on the genome hic file output by the correction module in the previous step. The resolution is extracted as follows:


\begin{eqnarray*}{\rm resolution }= \left\{ {\begin{array}{@{}*{1}{c}@{}} {{\rm resolutio}{n}_{i - 1}*1440 < {L}_g}\\ {{\rm resolutio}{n}_i*1440 \ge {L}_g} \end{array}} \right.\end{eqnarray*}

${\rm extract\ length} = ( {0,{L}_g} )$ , where ${L}_g$ represents the full length of the genome in the hic file. The matrix of contact is then presented as a contact image using the pyplot.matshow (parameters: A = $X$, cmap = red_map, vmin = 0, vmax = ${Q}_{95}$) function of matplotlib (v3.7.1). The red_map value was calculated using the color (LinearSegmentedColormap.from_list(‘bright_red’, [(1, 1, 1), (1, 0, 0)])) function of matplotlib (v3.7.1). ${Q}_{95}$ represents the 95th percentile of the contact matrix. The formula for its calculation is as follows:


\begin{eqnarray*}{Q}_{95} = \left\{ {\begin{array}{@{}*{1}{c}@{}} {{X}_k\ \left( {k \in \mathbb{Z}} \right)}\\ {{X}_{{\rm floor}\left( k \right)} + \left( {k - \left\lfloor {k}\right\rfloor} \right)*\left( {{X}_{{\rm ceiling}\left( k \right)} - {X}_{{\rm floor}\left( k \right)}} \right)\left( {k \notin \mathbb{Z}} \right)} \end{array}} \right.\end{eqnarray*}

$X$ is the entire contact matrix. $k$ is the index at position $0.95{\mathrm{*}}n$, where $n$ is the number of flattened contact matrix data points. $k$ denotes rounding $k$ down to the nearest whole number. $k$ denotes rounding $k$ up to the nearest whole number. ${X}_{{\rm floor}( k )}$ represents the value in the flattened array that corresponds to the $k$ integer value. ${X}_{{\rm ceiling}( k )}$ represents the value in the flattened array that corresponds to the $k$ integer value. $\mathbb{Z}$ represents the set of integers.

The generated global contact image is subsequently input into the detection model, which identifies the chromosome regions and returns the border value of each region in the contact image. AutoHiC utilizes this information, along with the error position conversion formula, to obtain the start and end information of each chromosome in the genome. AutoHiC cuts the genome sequence based on the start and stop information of each chromosome. This process involves sequentially cutting the scaffolds where the start and end sites of each chromosome are located and then connecting the scaffolds belonging to the same chromosome. Redundant sequences that do not belong to any chromosome are merged into a scaffold at the end of the genome.

### Assembly report

The assembly report is divided into four sections: summary, adjusting results, error adjustment and additional information. Each section utilizes different software or statistical methods, and then all results are consolidated into JSON format. Finally, Jjinja2 (v3.1.2) generates HTML pages using predesigned templates.

The Summary section presents the results obtained by QUAST (v5.2.0, parameters: –large -t threads) through the calculation of the AutoHiC-corrected genome results. The thread parameters are obtained from the user’s configuration file. The CC ratio is determined by dividing the number of scaffolds by the number of chromosome pairs in a genome assembly.


\begin{eqnarray*}CC\ {\rm ratio }= \frac{{{N}_{{\rm scaffolds}}}}{{{N}_{{\rm chromosomes}}}}\end{eqnarray*}

where ${N}_{{\rm scaffolds}}$ is the number of scaffolds and ${N}_{{\rm chromosomes}}$ is the number of chromosomes. The structural error ratio is derived from the error detection module, and its calculation formula is as follows:


\begin{eqnarray*}{R}_{{\rm error}} = \frac{{\mathop \sum \nolimits_{i = 1}^n {T}_i + \mathop \sum \nolimits_{j = 1}^m {I}_j}}{{{L}_g}}\end{eqnarray*}

The structural error ratio is represented by ${R}_{{\rm error}}$, where ${T}_i$ represents the length of each translocation, ${I}_j$ represents the length of each inversion, and ${L}_g$ represents the total length of the corrected genome sequence (excluding redundant sequences). The number of chromosomes is obtained from the result returned by the chromosome assignment module. The calculation formula for the Hi-C Anchor rate is as follows:


\begin{eqnarray*}{\rm Anchor}\ {\rm rate}\left( \% \right) = \ \frac{{{L}_{{\rm contig}}}}{{{L}_g}}*100\% \end{eqnarray*}

where ${L}_{{\rm contig}}$ represents the size of the genome before correction (user input).

The Adjusting Result displays the global contact images before and after correction. To obtain this information, the hic file is used before and after correction, employing the same method as in the chromosome assignment module. The Error Adjustment section delineates the categories of errors, translocation and inversion, highlighting the five most prevalent instances of each for exhibition. In instances where fewer than five errors are detected, all identified errors are presented. The accompanying imagery and locational data are derived from the error detection module.

The additional information comprises details about the genome prior to correction, the error rate for each error, the total number of errors and the image showing chromosome length proportions. The fundamental information of the genome was calculated prior to correction using QUAST. The method for calculating the structural error ratio is consistent with the method used in the summary. The formulas for calculating the error rates are as follows:


\begin{eqnarray*}{R}_{{\rm translocation}} = \frac{{\mathop \sum \nolimits_{i = 1}^n {T}_i}}{{{L}_g}}\end{eqnarray*}

\begin{eqnarray*}{R}_{{\rm inversion}} = \frac{{\mathop \sum \nolimits_{j = 1}^m {I}_j}}{{{L}_g}}\end{eqnarray*}

${L}_g$ represents the uncorrected genome size. During each correction process, the number of errors is determined based on the statistical results of the error detection module. The image showing chromosome length proportions was obtained by the AutoHiC Python script (gen_chr_fig.py).

### Peak algorithm

The main task of the peak algorithm (Supplementary Figure S22) is to find insertion sites for translocation errors. The contact matrix data containing the error and insertion site are extracted using hicstraw (v1.3.1, parameters: contact data = getMatrixZoomData(‘assembly’, ‘assembly’, ‘observed’, ‘NONE’, ‘BP’, $Resolutio{n}_{best}$); $X$ = getRecordsAsMatrix(0, ${L}_g$, $Erro{r}_{start}$,${\mathrm{\ }}Erro{r}_{end}$); $R{\mathrm{\ }}$= getResolutions()), based on the length of the error and the best resolution. The formula for the best resolution is as follows:


\begin{eqnarray*}Resolutio{n}_{best} = argmin\left\{ {abs\left( {\frac{{error\ length}}{3} - {R}_i} \right)} \right\}\ \left( {{R}_i \in R} \right)\end{eqnarray*}

\begin{eqnarray*}error\ length = \ Erro{r}_{end} - Erro{r}_{start}\end{eqnarray*}

where ${L}_g$ is the total length of the genome hic file and $Erro{r}_{start}$ and $Erro{r}_{end}$ represent the start and end positions of the error on the contact matrix, respectively, from the results returned by the error detection module. $R$ is an array composed of various resolutions obtained by the above function.

The find_peaks (parameters: ${X}_i,$ height = $Pea{k}_{95}$, distance = $distance\ threshold$) algorithm of SciPy (38) (v1.10.1) is subsequently used to obtain the peak’s index of each row ($P$) in the contact matrix in a loop. ${X}_i$ is a subset of the error contact matrix ($X$) that was extracted earlier and contains only one row of contact values. $Pea{k}_{95}$ represents the 95th percentile of the contact peak value, while $distance{\mathrm{\ }}threshold$ indicates the minimum distance between each pair of peaks. These values are calculated as follows:


\begin{eqnarray*}distance\ threshold &=& \left\lfloor {\frac{{Erro{r}_{end}}}{{Resolutio{n}_{best}}}} \right\rfloor - \left\lfloor {\frac{{Erro{r}_{start}}}{{Resolutio{n}_{best}}}} \right\rfloor \nonumber\\ &&\times \ \ \left( {distance\ threshold \ge 0} \right)\end{eqnarray*}

\begin{eqnarray*}Pea{k}_{95} = \left\{ {\begin{array}{@{}*{1}{c}@{}} {{X}_k\ \left( {k \in \mathbb{Z}} \right)}\\ {{X}_{floor\left( k \right)} + \left( {k - \left\lfloor{k}\right\rfloor} \right)*\left( {{X}_{ceiling\left( k \right)} - {X}_{floor\left( k \right)}} \right)\left( {k \notin \mathbb{Z}} \right)} \end{array}} \right.\end{eqnarray*}

$X$ is the contact matrix. The index of the error’s peak ($Pea{k}_{error}$) is then calculated.


\begin{eqnarray*}Pea{k}_{error} = \left[ {\left\lfloor {\frac{{Erro{r}_{start}}}{{Resolutio{n}_{best}}}} \right\rfloor \ - 2,\left\lfloor {\frac{{Erro{r}_{end}}}{{Resolutio{n}_{best}}}} \right\rfloor \ + 2\ } \right]\end{eqnarray*}

We remove values less than $Pea{k}_{95}$ from $P$ and eliminate values in $Pea{k}_{error}$. The value with the highest number of repetitions ($i$) was selected, which is the index value of the translocation error insertion site. Finally, the insertion position $I$ is determined based on the insertion index ($i$) using the following formula:


\begin{eqnarray*}I = i*\frac{{{L}_g}}{{Resolutio{n}_{best}}}\end{eqnarray*}

### AutoHiC model

The AutoHiC model consists of four components: the Backbone (Swin Transformer), Neck (FPN), Dense Head (RPN) and region of interest (RoI) Head.

The Swin Transformer is used as the backbone network to extract high-level features from the input contact images. It has an embedding dimension of $96$ and a depth configuration of $[ {2,{\mathrm{\ }}2,{\mathrm{\ }}6,{\mathrm{\ }}2} ]$. Different layers have varying numbers of attention heads $[ {4,{\mathrm{\ }}8,{\mathrm{\ }}16,{\mathrm{\ }}32} ]$, and a window size of $7$ is utilized in the attention mechanism. The MLP (Multilayer Perceptron) ratio is set to $4.0$ to control the dimensions of the hidden layers in the multilayer perceptron. For improved generalization of the network, dropout and drop path (drop_rate = 0, drop_path_rate = 0.2) mechanisms are also utilized.

The feature pyramid network (FPN) receives high-level feature maps from the Swin Transformer backbone, which are characterized by channel dimensions of $[ {96,{\mathrm{\ }}192,{\mathrm{\ }}384,{\mathrm{\ }}768} ]$. It yields an output with a channel dimension of 256. The FPN generates a comprehensive set of five hierarchical feature maps as its output.

The region proposal network (RPN) takes feature maps with an input channel dimension of 256. These feature maps undergo further transformation within the RPN to achieve the same channel dimension. The AnchorGenerator generates anchor boxes (scales = $[ 8 ]$, ratios = $[ {0.5,{\mathrm{\ }}1.0,{\mathrm{\ }}2.0} ]$, strides = $[ {4,{\mathrm{\ }}8,{\mathrm{\ }}16,{\mathrm{\ }}32,{\mathrm{\ }}64} ]$). The DeltaXYWHBBoxCoder is responsible for transforming the anchor box coordinates into encoded representations (target_means = $[ {.0,{\mathrm{\ }}.0,{\mathrm{\ }}.0,{\mathrm{\ }}.0} ]$, target_stds = $[ {1.0,{\mathrm{\ }}1.0,{\mathrm{\ }}1.0,{\mathrm{\ }}1.0} ]$). The regression loss is computed using the smooth L1 loss (beta = 1.0/9.0, loss_weight = 1.0). This loss function is applied to the bounding box regression.

The RoI head processes the candidate object boxes from the RPN in three stages. First, the BBox RoI extractor uses RoIAlign to extract features from the RoIs. The features are subsequently used for classification and bounding box regression. Finally, the output includes the classification and bounding box information for the object box ($X$).

The model takes multichannel contact images (from the error detection module) and identifies errors in each image. If errors are present, the model returns a list of information $X$ for each error.


\begin{eqnarray*}X = \left[ {type,{\mathrm{\ }}x,y,w,h,score} \right]\end{eqnarray*}

$type$ refers to the error type, while $x$ and $y$ represent the coordinates of the upper left corner of the error prediction box. $w$ and $h$ indicate the width and height of the prediction box, respectively. $score$ denotes the confidence score of the prediction category. This information is utilized by the error detection module for further processing.

The deep learning model is implemented using MMDetection (39) (v.2.11.0) and PyTorch (v1.10.1) in Python (v.3.9). The detailed configuration files for the model can be obtained from https://github.com/Jwindler/AutoHiC/blob/main/src/models/cfgs/error_cascade_mask_rcnn_sswi_fpn.py. For comprehensive technical details, readers can refer to the implementation code (https://github.com/Jwindler/AutoHiC).

### Sliding window

The sliding window algorithm is based on a set size and extracts the local contact matrix by sampling the contact matrix at different resolutions. For different resolutions, the size of the sliding window is $M{\mathrm{\ }}( {resolution{\mathrm{*}}700} )$, and the sliding step is $N{\mathrm{\ }}( {resolution{\mathrm{*}}400} )$. The whole process is performed along the diagonal of the contact matrix from the top left to the bottom right.

In the initial phase of the algorithm, an initial window is established by selecting the first $M \times N$ elements of the contact matrix. The elements within the window are then extracted through hicstraw (parameters: contact data = getMatrixZoomData(‘assembly’, ‘assembly’, ‘observed’, ‘NONE’, ‘BP’, $resolution$); contact matrix = getRecordsAsMatrix($N{\mathrm{*}}( {n - 1} ),{\mathrm{\ }}N{\mathrm{*}}( {n - 1} ) + M,{\mathrm{\ }}N{\mathrm{*}}( {n - 1} ),{\mathrm{\ }}N{\mathrm{*}}( {n - 1} ) + M$)) for visualization as contact images. The window is then shifted along the entire contact matrix by a step size of $N{\mathrm{\ }}( {resolution{\mathrm{*}}400} )$, iteratively repeating the operation on the new window. This process continues until the entire contact matrix is covered.

### Datasets

The dataset used in this article is divided into three parts: the Hi-C original contact matrix (in hic format), contact images for model training and genomic data used for testing AutoHiC.

This dataset includes the original contact files in Hi-C format. which can be downloaded from the DNA Zoo (15). A total of 324 hic files were downloaded, and 21 additional hic files were generated using Juicer (v1.6; parameters: -S early) (40) and 3D-DNA. The data generated for model training using the Python script were not included. Supplementary Table S10 contains detailed information on the raw data obtained from the DNA Zoo.

The data used for model training and testing in the second part are the contact image data. The contact matrix can be extracted from the hic files using hic-straw. The same method as in the error detection module was used to convert these contact matrices into local contact images. Approximately 500 000 local contact images were obtained and selected using the proposed error classification model. Ultimately, 12 049 images were chosen and labeled; these included 5673 chromosomes, 1783 debris, 1155 inversions and 3438 translocations (Supplementary Text S5). These numbers represent only the quantity of images used to train or test the error detection model, not the quantity of each type of error. It is possible for multiple chromosomal regions or errors to occur in a single contact image. During model training, the data were augmented using techniques such as zooming, cropping and flipping.

The genome data included contig-level genomes, Hi-C sequencing data and reference genomes, which were downloaded from the DNA Zoo and NCBI databases. Details regarding the genome and Hi-C sequencing data can be found in Supplementary Table S9.

### Generation of training data

The data used for training the model were generated and simulated based on collected Hi-C data. The generated data were created using a script that follows predetermined length gradients and resolutions, while the simulated data were produced by randomly adjusting conatct matrix values derived from actual data, resulting in data that closely resemble real-world observations. Three main steps were used to generate the simulation data. First, the genomic contig sequences were cut into sequences ranging from 10 to 50 kb in length. Second, a python script was used to randomly select 60% of the sequences, after which the sequences were randomly moved or flipped. Finally, the Hi-C sequencing data were realigned to these cut genomes. The corresponding hic files were obtained, and the contact matrix and visualization were extracted using hicstraw and matplotlib for model training. The color thresholds applied to the contact heatmaps were determined by extracting the 95th quantile of the contact matrix distribution.

### Image data preprocessing

The initial data were subjected to preprocessing to convert them into local contact heatmaps represented in jpg format. Due to the substantial volume of generated data, direct utilization for model training was unfeasible. To address this, a classification network was implemented to curate usable images from the generated dataset.

Subsequently, manual annotation was performed on the selected data using the labelme (v.5.3.1, https://github.com/labelmeai/labelme) tool. To ensure proper training, the outer border of each type was selected in labelme for every contact image and labeled accordingly. Then, the script labelme2coco.py was used to convert the labeled data to the COCO format. Prior to the training phase, data augmentation procedures were applied, including transformations such as flipping, rotation, scaling, cropping and shifting. These augmentations are designed to amplify the diversity and robustness of the training dataset.

### Classification network

Because many original contact images were generated, only a small portion of the images could be used to construct the model. To address this, we filtered the data using a classification model. The classification model was based on EfficientNetV2 (41) (https://github.com/WZMIAOMIAO/deep-learning-for-image-processing) and was built using open-source code. Then, 2000 contact images were manually selected and categorized for training the model. The categories included translocation (400), inversion (400), debris (400), white (400) and other (400). The classification model was trained using these datasets. After a total of 200 epochs, the model tended to converge, resulting in an overall accuracy of 0.9. This model was subsequently used for data classification. To use AutoHiC for image classification, all contact images are simply placed in a designated folder. The program will then call the classification model to judge each image and mark its type. Similar contact pictures will be moved to a separate file for data labeling.

### Model training

The dataset used for model training was derived from data that had been filtered by the classification model. These data were manually labeled and used for training. The dataset was divided into two distinct subsets. Approximately 85% of the data were used as the training set to iteratively refine the model parameters. The remaining portion was allocated as the test set, which was intended to meticulously assess the performance of the trained models. The dataset used during training is completely shuffled and contains cross-species data.

To train the network, we used the stochastic gradient descent (SGD) optimizer (learning rate = 0.02, momentum = 0.9, weight decay = 0.0001). Our approach utilizes a stepwise learning rate scheduling strategy that includes linear warm-up and two steps of learning rate adjustment. The linear warm-up gradually increases the learning rate over the first 500 iterations, which helps the model adapt better to the training data. Adjustments occur at the end of the 8th and 11th training stages, introducing a dynamic mechanism for adapting the learning rate to optimize the convergence and stability of the model training process.

Due to the updating of the dataset, the model underwent a total of nine iterative trainings, with each training consisting of 200 epochs. The pretrained model from each training session was used as the optimal model for subsequent training. On average, a training session lasted for two days. Training was conducted on NVIDIA GeForce RTX 3080 Ti GPUs. After training the model, the script ‘analyze_logs.py’ (parameters: ‘plot_curve’, ‘–keys loss’) was used to obtain the changes in accuracy and loss during the training process. The pyplot function from the matplotlib library was subsequently used to visualize the training curve.

### Model evaluation

Furthermore, we assessed the generalization proficiency of AutoHiC through an evaluation conducted on an independent test subset that was entirely segregated from the training process. To evaluate the detector efficacy after training, we leveraged both the confusion matrix and the area under the precision−recall curve (AUPRC). For each pair of adjacent precision and recall rates, the values were treated as the upper and lower bases of a trapezoid, and the area of the trapezoid was calculated to estimate the area under the PR curve at the current stage. The areas of all the trapezoids were calculated and added to obtain an approximation of the area under the PR curve. During the cross-validation procedure, we judiciously selected the hyperparameters that yielded the highest AUPRC, ensuring the optimal configuration settings. The AUPRC was generated for the reserved dataset with a script (pr.py; parameters: –eval-options ‘classwise = True’, –eval bbox proposal) from MMDetection. The confusion matrix was generated by the pyplot drawing function (plot_confusion_matrix.py) in matplotlib.

Intersection over union (IOU) (Supplementary Figure S7) is a metric employed to quantify the overlap between two error bounding boxes. It is expressed as the ratio of the area of their intersection to the area of their union. The IOU is calculated using the following formula, given the coordinates of two bounding boxes $( {{x}_1,{\mathrm{\ }}{y}_1,{\mathrm{\ }}{x}_2,{\mathrm{\ }}{y}_2} )$ and $( {x_1^{\mathrm{{\prime}}},{\mathrm{\ }}y_1^{\mathrm{{\prime}}},{\mathrm{\ }}x_2^{\mathrm{{\prime}}},{\mathrm{\ }}y_2^{\mathrm{{\prime}}}} )$:


\begin{eqnarray*}IOU = \frac{{{\rm Intersection}{\mathrm{\ }}{\rm Area}}}{{{\rm Union}{\mathrm{\ }}{\rm Ares}}}\end{eqnarray*}

\begin{eqnarray*}IOU = \frac{{\left( {{x}_2 - x_1^{\mathrm{{\prime}}}} \right){\mathrm{*}}\left( {y_2^{\mathrm{{\prime}}} - {y}_1} \right)}}{{\left( {{x}_2 - {x}_1} \right){\mathrm{*}}\left( {{y}_2 - {y}_1} \right) + \left( {x_2^{\mathrm{{\prime}}} - x_1^{\mathrm{{\prime}}}} \right){\mathrm{*}}\left( {y_2^{\mathrm{{\prime}}} - y_1^{\mathrm{{\prime}}}} \right) - Intersection{\mathrm{\ }}Area}}\end{eqnarray*}

The intersection area refers to the area shared between two error bounding boxes, while the union area represents the combined area encompassed by the boxes. A higher IOU indicates greater overlap, which in turn suggests more accurate alignment between the predicted and ground truth bounding boxes. Conversely, a lower IOU value suggests a reduced degree of overlap.

### AutoHiC algorithm verification

To assess the feasibility of the AutoHiC error correction algorithm, we employed a comparative approach involving the generation of contact heatmaps and contact curves before and after the error correction process.

To extract the contact matrix corresponding to the interval in which the error is located, we used hicstraw. We then visualized the contact matrix as a heatmap using matplotlib. Additionally, we intercepted the local matrix where the error was located from the contact matrix by combining the contact matrix and error information. Finally, we visualized the local contact matrix as a curve using matplotlib. For ease of comparison, we used two resolutions. The determination of resolution is indicated above the contact heatmap. In the contact heatmap, specific areas of the contact matrix are shown. Notably, the intervals between the pre- and postcorrection phases remained consistent. While the data within the contact matrix remained unnormalized, the extracted contact matrix underwent normalization to the range of 0 to 1, subsequently facilitating the generation of the associated interaction curve.

### Scaffolder tool search

To find all available (recent) Hi-C scaffolders, we performed a literature search in PubMed for publications between 27 March 2020 and 27 March 2023. The search terms ‘(hi-c scaffolding) or (hi-c assembly) or (hi-c genome assembly) or (hi-c scaffolding) or (hi-c assembly) or (hi-c genome assembly)’ yielded 699 results. Nine scaffolding methods were identified for publishing genomes (Supplementary Table S1). We retained only four software programs, among which support was no longer provided for Lachesis, and the official website recommended using 3D-DNA instead. instaGRAAL can only run in a GPU environment. A software error occurred while running EndHiC, and the author did not respond to a request for assistance. HiRise (9) does not need to use Hi-C data. AllHiC is used mainly for plant genome assembly and requires genomes from homologous species.

### Benchmarking the performance of AutoHiC

All the compared software programs were obtained, installed, and evaluated by following the procedures outlined in the provided publication sources. When assessing the performance of different software programs on the same species (*Caenorhabditis elegans*, *Arabidopsis thaliana*, *Drosophila melanogaster*, *Danio rerio* and *Homo sapiens*), uniform datasets were used to ensure consistency. The input data for software such as SALSA2 (v2.3), YaHS (v1.1) and Pin_hic (v3.0.0) consisted of BAM or BED files generated by the HiC-Pro (42) (v3.1.0) pipeline. The parameter configurations for each of the compared software programs were maintained at their default settings as stipulated by the original documentation for each program.

The genome contiguity assessment predominantly relied on key metrics such as the N10-N90, L50, NGA50 and NG50 values and the CC ratio. Specifically, the N10−N90 and L50 values were computed utilizing the QUAST software package (Supplementary Tables S2–S4). The parameter settings for all compared software were retained in their default states, as prescribed in the original documentation. The CC ratio, representing the ratio of the scaffold number to the chromosome number, was established manually.

For the quantitative analysis of structural variations, a reference genome was selected from Supplementary Table S9. The analysis was conducted using MUM&Co software (v3.8), maintaining the parameter configurations in line with the default specifications provided in the original documentation.

### Synteny analysis

To conduct a comparative analysis, we evaluated the 3D-DNA assembled genomes and those corrected by AutoHiC against their respective T2T reference genomes. This evaluation involved using the MUM&Co tool to capture structural variation information. We then visualized the structural variation regions within the 3D-DNA assembly outcomes using NGenomeSyn (43) (v.1.41). We used SeqKit (44) (v.2.5.1) to extract sequences that contained structural errors. BLASTn (-outfmt 6, -evalue 1e-5) was subsequently used to compare these sequences with the genomes adjusted by AutoHiC. This comparison allowed us to obtain corresponding sequence positional information. We filtered the comparison results based on sequence length and alignment consistency. We used SeqKit to extract the aligned sequences, enabling thorough comparison and collinearity analysis with the T2T reference genome. The dot plot is generated as follows. First, use Minimap2 (v2.26-r1175 -x asm5) (45) was used to obtain the paf file of the whole-genome alignment. Then, pafCoordsDotPlotly (v -s -t -l, https://github.com/tpoorten/dotPlotly) was used to generate the corresponding dot results.

### Genome assembly with AutoHiC

During AutoHiC testing, only the original genome and Hi-C sequencing data were needed. AutoHiC automatically performs error correction and generates result reports. The genome and contact matrix files before and after correction are available in the AutoHiC results for visualization and statistical analysis. The number of assembly errors can be directly obtained from the results. According to the genome size, the Hi-C data size and the running time of AutoHiC for the number of correction iterations can be obtained from Supplementary Table S11. AutoHiC provides two chromosome-level genome files (Supplementary Table S12), taking into account the presence of redundant sequences in the Hi-C data. Users can choose which file to use based on the description provided on GitHub. The species (*Pholis gunnellus*, *Taurulus bubalis* and *Thunnus maccoyii*) were sequenced using long-read sequencing technology. Hifiasm was utilized to construct contig-level genomes from scratch, and AutoHiC was used for correction. The assembly process included the following sequence of steps: initial PacBio assembly generation with Hifiasm (v0.16.1-r375, parameters: ‘-t 20 -a 4 -m 10000000 -p 0 -n 3 -x 0.8 -y 0.2 –hom-cov auto –lowQ 70 –b-cov 0 –h-cov 1 -O 1 –purge-max auto –n-hap 2″) (46,47); retained haplotig separation with purge_dups; if there is ONT data were available, the products were added to the hifisam assembly at the same time; longranger basic (v2.2.2, default parameters) was used to preprocess the 10× original data, and tigmint(v1.2.10, parameters: ‘-j 40″) was used to correct the haplotigs based on the preprocessed 10× data.

## Results

### Overview of the AutoHiC pipeline

AutoHiC harnesses the knowledge derived from manual correction within assembly tools (48,49) to provide a fully automated, deep learning-based approach to identifying and correcting misassembled genomes. At the macroscopic level, AutoHiC operates in three stages (Supplementary Figure S1). The first stage involves leveraging 3D-DNA (15) or other scaffolders to generate preliminary assembly results based on the existing contig and Hi-C reads. The outcomes of this step include scaffolds (uncorrected) and Hi-C contact matrix at various resolutions. In the second step, AutoHiC includes an error detection and correction module to improve assembly quality. The module effectively corrects translocation and inversion errors at their identified locations. Based on the model’s ability to detect chromosome areas, the chromosome assignment module divides the previously corrected genome into a chromosome-level genome. In the final stage, a visual report is generated to show the genome before and after error correction. This report allows users to evaluate genome quality, analyze relevant metrics and review details of the error correction process for subsequent analyses.

### Assembly report of AutoHiC

The assembly result report provides users with a comprehensive understanding of their genome, including a detailed assessment of AutoHiC corrections. We compiled a report detailing the outcomes from a single test species (*Brassica rapa*). Structurally, the report is divided into four sections, each addressing distinct facets of the genomic correction process. The initial section of the report describes genome summary statistics such as N50, L50, Hi-C anchor rate, scaffold number and GC content (Supplementary Figure S2). This section is instrumental in providing an overview of the genome’s characteristics after AutoHiC correction. The next section compares the genome-wide contact heatmaps before and after the error correction process. This analysis provides insight into the effectiveness of AutoHiC corrections in improving the reliability of genomic contact (Supplementary Figure S3). The third section discusses the details of the error correction process, including the type, dimensions and positional coordinates of the identified errors within the genome (Supplementary Figure S4). Finally, the last section of the report contains Supplementary Data. This section is instrumental in recording and tracking the change in errors during the correction process (Supplementary Figure S5). The assembly report results serve as a crucial tool for comprehending the genome quality and error correction outcomes.

### The AutoHiC model

**Figure 1.**
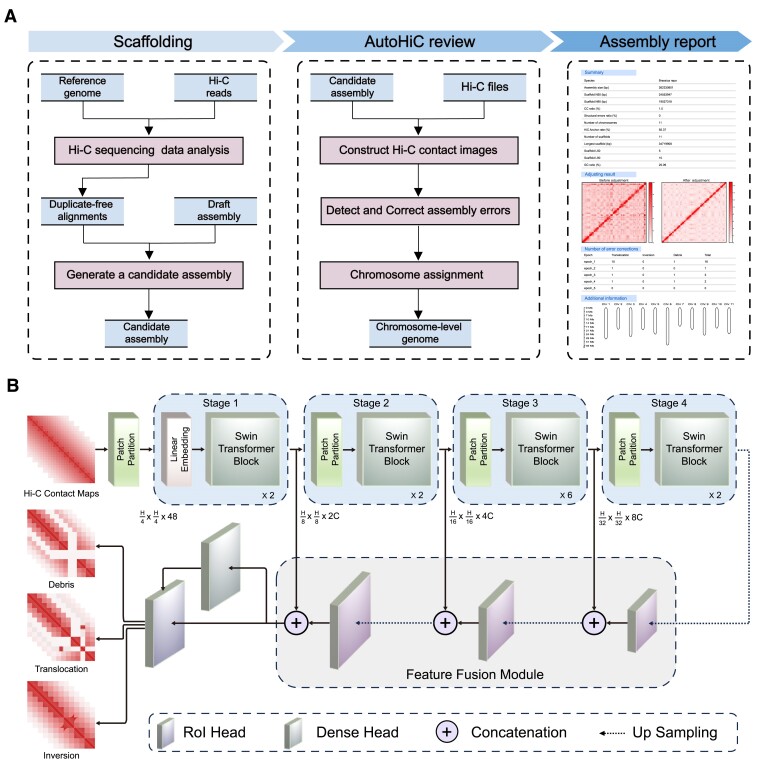
Overview of the AutoHiC framework. (**A**) AutoHiC runs processes and data flows. The AutoHiC assembly process is divided into three main steps. Each step uses different data, and the initial input only requires Hi-C sequencing data and genome. (**B**) AutoHiC model description. The architecture of the Swin Transformer network employed in AutoHiC is depicted. The input is a three-channel contact image, and the outputs are the types of errors detected by the model and the corresponding location information. Different layers adopt different configurations, which are distinguished by colors, and the specific information subscripts are visible.

Accurately identifying the type of error and its location is crucial for correcting assembly errors. The AutoHiC model (Figure 1B), as part of the detection module, is responsible for detecting errors in contact images and providing positional error information. The AutoHiC model is a deep neural network that uses two-stage object detectors to detect genome assembly errors and extract error features by leveraging Hi-C contact heatmap images.

The AutoHiC model comprises four essential components: Backbone, Neck, Dense Head and RoI Head. To extract error features from contact images more effectively, we use the Swin Transformer (50) as the backbone network of AutoHiC. This network adopts a layered transformer structure, dividing the image into different blocks and performing self-attention (30) operations within each block. This hierarchical architecture enables efficient handling of large-scale Hi-C contact images. To reduce the computational complexity, the model uses a windowed attention mechanism that limits self-attention operations to local windows within each block instead of performing global computations. Furthermore, it introduces a deep cross-local area attention mechanism to establish long-range dependencies among different blocks, capturing a broader range of contextual information. To facilitate feature transformation and fusion, AutoHiC integrates the FPN (51) as a neck. The FPN is adept at handling visual tasks at various scales and layers, making it an ideal choice for the AutoHiC architecture. Finally, for the precise screening and localization of the extracted error features, the RPN (52) and cascade RoI head are employed.

A trained AutoHiC model can scan the entire Hi-C contact image and extract error information for each image, including the locations and types of errors in the image. The error detection module utilizes information from AutoHiC model results that are mapped back from the image to the scaffold genome coordinates. Finally, the error correction module corrects the detected errors based on the provided information.

### Principles of the AutoHiC algorithm

The ability of the AutoHiC to correcting errors and assigning chromosomes, which are crucial in genome assembly. The AutoHiC algorithm effectively locates erroneous regions in misassembled scaffolds by retrieving relevant contact matrix based on the error position information provided by the model. The error correction module rectifies errors using information provided by the model in conjunction with the Hi-C contact matrix.

**Figure 2.**
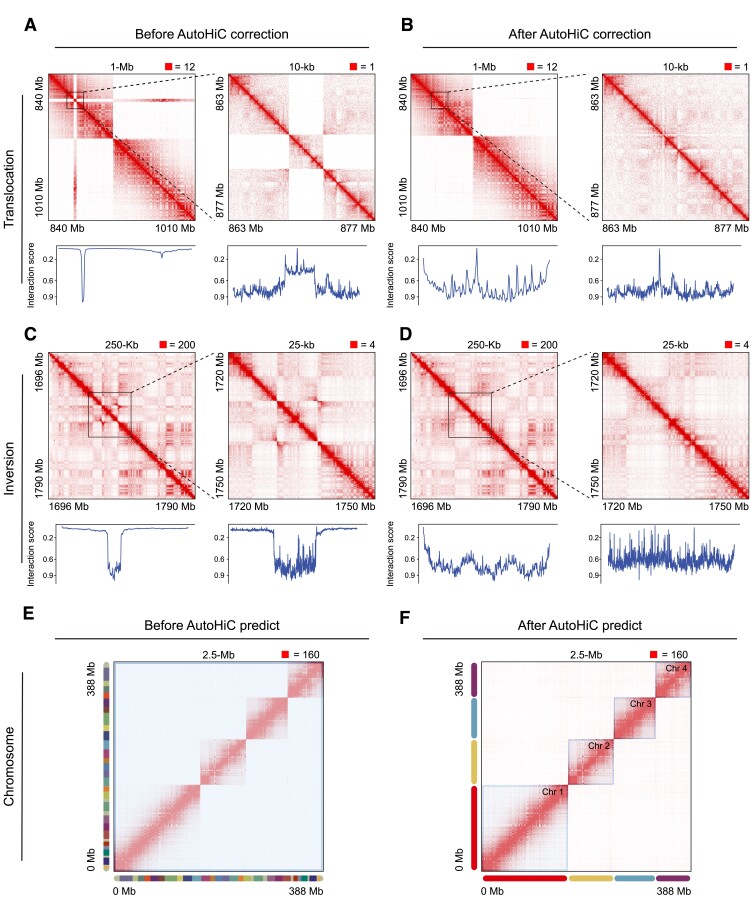
Comparison of AutoHiC error correction effects. (**A,C**) Contact heatmaps and contact curves with assembly errors. (**B,D**) Contact heatmaps and curves after error correction without assembly errors. The contact heatmap is at two resolutions, with the high resolution on the right. The resolution and contact boundaries are marked at the top. The lower boundary has a marker indicating the extent of the contact area. Contact curves were generated from the contact matrix used in the contact heatmap. The contact curves in the figure represents the interaction scores. The contact values are normalized to 0–1. (**E,F**) Global contact heatmaps before and after AutoHiC chromosome assignment. Each rounded rectangle, distinguished by a unique color, represents a distinct scaffold (left) or chromosome (right). The area surrounded by the blue box is the contact area within the chromosome (right).

For translocations, the AutoHiC algorithm extracts the regions characterized by the error detection model. The contact curves are then generated for the corresponding regions, and the interaction pattern of the contact curve corresponds to the contact heatmap (Figure 2A). The contact curve indicates the location of each translocation error and the site at which an insertion should be made (as indicated by the arrow). AutoHiC eliminates peaks at these sites and filters redundant peaks to determine the exact insertion site of the translocation error due to the presence of interfering signals (‘Materials and Methods’ section). AutoHiC moves the sequence of the region where translocation occurred to the corresponding insertion site. As shown in Figure 2B, translocation errors were eliminated in the contact heatmap after AutoHiC adjustment. Inversions exhibit distinct characteristics in both contact heatmaps and contact curves (Figure 2C). Specifically, the area where inversion error occurs has a butterfly shape in the contact heatmap, and the corresponding contact curve has a peak area of a certain length. To correct this, AutoHiC adjusts the sequence interval and length based on error information from the detection module and then reverses the sequence in the affected area. After adjustment, the algorithm effectively eliminates the features in the contact heatmap and the peaks on the contact curve (Figure 2D), demonstrating its ability to handle inversion errors. In addition to identifying and rectifying genome misassembly, AutoHiC can be applied to chromosome assignment. After correcting all the errors, the chromosome assignment module infers the number of chromosomes in the genome by utilizing a global contact heatmap (Figure 2E). Subsequently, the genome sequences were assigned to the respective chromosomes (Figure 2F).

### Performance evaluation of AutoHiC

**Figure 3.**
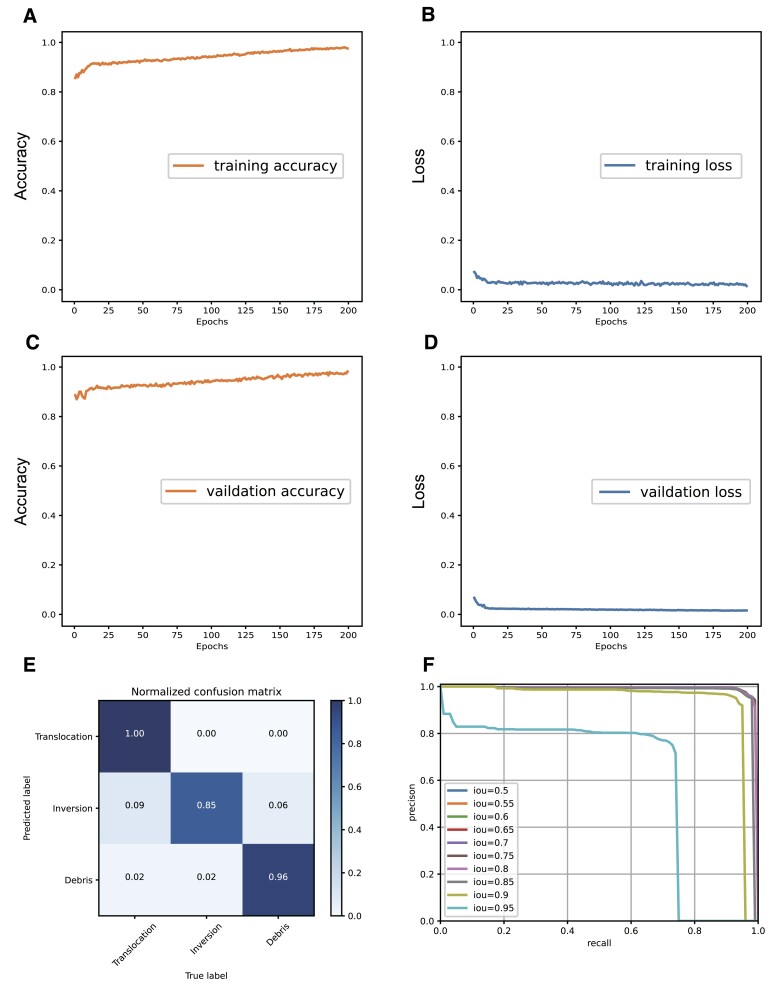
AutoHiC model effect verification. (**A–D**) Changes in model accuracy and loss rate during training and validation. The training process is shown on the left, and the verification process is shown on the right. The lower curve is the training loss and the upper curve is the training accuracy. (**E**) Confusion matrix results for the AutoHiC error detection model. The horizontal axis of the graph represents the actual error, and the vertical axis represents the model-predicted error. The diagonal line is the correct prediction result, and the darker the color of the cube is, the more images are correctly predicted. (**F**) Precision−recall curve for the different IOU thresholds (0.5–0.95) as in the panel. Curves represent the PR results at different IOU thresholds.

The model was tested and verified using the prereserved test dataset from the training process. We present the training results of AutoHiC for assembly error detection and chromosome detection (Supplementary Figure S6). The accuracy and loss during model training are depicted to illustrate the convergence and fitness of the model to the training data after 200 epochs (Figure 3A–D), which indicates an effective learning process. To assess the model’s performance comprehensively, we employ the confusion matrix (53) and precision–recall (PR) curve (54). The confusion matrix provides valuable insights into the model’s performance across different classes (translocation, inversion and debris). Evaluating the model’s performance for each specific class offers an internal perspective on its effectiveness, yielding a more nuanced assessment than that obtained by considering overall accuracy. Notably, the confusion matrix (Figure 3E) indicates that the model predicts translocation, inversion and debris with image accuracies exceeding 90%, while inversion prediction achieves 85% accuracy.

The consistent patterns observed in the confusion matrix align with the dataset’s regularity, validating the reliability of the AutoHiC model for error detection. Furthermore, we compared the performance of the AutoHiC model using the PR curve (Figure 3F) and the area under the curve (AUC). Multiple PR pairs were calculated by varying the IOU thresholds (0.5–0.95), and visualizing them allowed us to compute the AUC using the composite trapezoidal rule. A higher AUC, ranging from 0 to 1, indicates superior model performance. The PR curve shows that when the IOU threshold is set between 0.5 and 0.95, the AUC approaches 1, highlighting the model’s strong performance.

### AutoHiC outperforms other methods in improving genome contiguity

**Figure 4.**
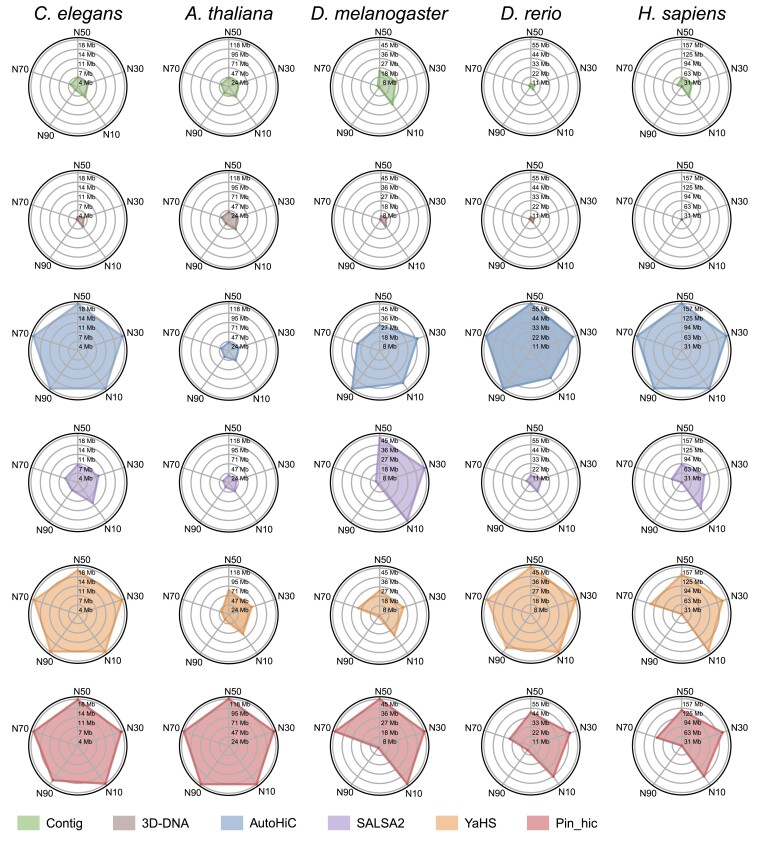
Comparison of genome contiguity benchmarks. The radar chart shows the contiguity of the assembly results. Each row represents the different software programs. The radar chart shows N10, N30, N50, N70 and N90. The different species are shown separately.

To further benchmark (55) the performance of AutoHiC, we compared AutoHiC with several competitive and representative tools, including 3D-DNA, SALSA2, YaHS and Pin_hic (Supplementary Table S1). Our primary goal was to evaluate the ability of AutoHiC to improve genome contiguity. To achieve this goal, we selected five species: *C. elegans*, *A. thaliana*, *D. melanogaster*, *D. rerio* and *H. sapiens*. These species were selected because they provide a good representation of currently studied model species, including plants and animals. The current tool used for contiguity assessment is N50 (56), which indicates the degree of contiguity of the genome assembly and is defined by the length of the shortest contig for which longer and equal length contigs cover at least 50% of the assembly. Higher N50 values indicate better contiguity. Additionally, the L50 value corresponds to the N50 value and indicates the number of contigs (or scaffolds) required to achieve the N50 value. Lower L50 values indicate better contiguity. Therefore, we calculated the Nx (Figure 4) and L50 values of these software assembly results using QUAST (57).

AutoHiC demonstrated exceptional performance against five species, achieving the highest N50 in *C. elegans*, *D. rerio* and *H. sapiens* (Figure 4). However, its performance in *A. thaliana* and *D. melanogaster*, was suboptimal, prompting a thorough investigation (Supplementary Text S4). Analysis of the assembly results by visualizing each scaffold length revealed that, during *A. thaliana* assembly (Supplementary Figure S10), YaHS merged two scaffolds, whereas Pin_hic combined nearly all the sequences into a single scaffold. In the *D. melanogaster* assembly (Supplementary Figure S11), the scaffold lengths from SALSA2 and Pin_hic significantly deviated from the expected *D. melanogaster* chromosome lengths. Further examination of the assembly results for *C. elegans*, *D. rerio* and *H. sapiens* showed no irregularities, with AutoHiC performing strongly well (Supplementary Figure S9, S12 and S13). Compared to the raw data and the outputs of the other software programs, AutoHiC improved the contiguity of the data by approximately 18-fold (compared to Contig) and 7-fold (compared to SALSA2).

For a more robust assessment of contiguity, we also introduce the L50 (Supplementary Figure S14) and CC ratio (58). The CC ratio results (Supplementary Figure S15) showed that the outputs from all the software tools had significantly greater CC ratio, except for AutoHiC, which remained at 1. This finding showed that the genome assembled with AutoHiC had high contiguity at the chromosome level. Additionally, we provided dot plots against the reference genome (Supplementary Figure S23–S47). Based on the aforementioned evaluation results, AutoHiC significantly enhances genome continuity and ensures accurate assembly.

### Validation of the AutoHiC results

Owing to the absence of experimental conditions, we conducted a comparative analysis of the genome, *H. sapiens* (59), before and after AutoHiC correction, against the existing T2T reference genome to evaluate the ability of AutoHiC to improve genome accuracy and contiguity. The selection of the T2T reference genome was based mainly on the following rationale. First, the T2T reference genome accurately reflects the actual size of the genome and can be compared with the adjusted results from AutoHiC. Second, we can check whether AutoHiC is overtuned by comparing the number of structural variations before and after AutoHiC correction.

**Figure 5.**
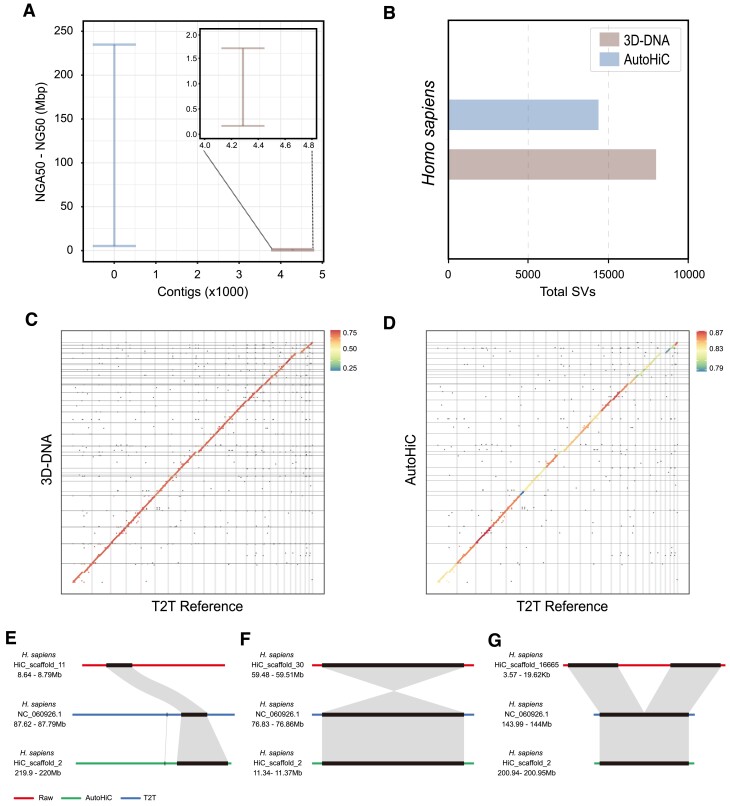
Assessment of the performance of AutoHiC correction. (**A**) Comparison of the contiguity of assemblies scaffolded by 3D-DNA and AutoHiC as measured by QUAST. The *Y*-axes show the range of NGA50 to NG50 lengths to indicate the uncertainty caused by true genomic variation between the individual and the reference genome. (**B**) Comparison of the accuracy of assemblies scaffolded by 3D-DNA and AutoHiC as measured by MUM&Co. The *X*-axis shows the total number of structural variations, reflecting the ability of AutoHiC to correct errors. (**C,D**) T2T whole-genome alignment dot plot. Species names are marked at the top. The left side shows the comparison results between 3D-DNA and T2T. The right side shows the comparison results between AutoHiC and T2T. The shading reflects the sequence consistency. The legend is at the top right, and the left and right areas are different. (**E–G**) Collinearity block plots before and after three rounds of error correction for translocation, inversion and deletion. The uncorrected collinearity with the T2T genome is shown above. Below is the AutoHiC-corrected collinearity with the T2T genome in the same region. The names and positions of the sequences are indicated on the left.

Initially, we utilized QUAST to analyze the assembled genome before and after AutoHiC error correction (Supplementary Table S5). Remarkably, AutoHiC significantly improved genome contiguity (Figure 5A), with the number of scaffolds in the corrected genome approaching the number of chromosomes (approximately equal to the number of chromosomes). The NGA50 and NG50 values also greatly improved. Additionally, we employed MUM&Co (60) to compare the assembly results with those of the T2T reference genome and quantified the number of structural variations (Figure 5B; Supplementary Tables S6 and S7). This evaluation partly reflects the accuracy and completeness of the assembly software. Notably, compared to the assembly results from other software, the AutoHiC assembly results showed the fewest structural variations, highlighting the superiority of AutoHiC in improving genome accuracy and indicating that there was no over-tuning of AutoHiC. If AutoHiC is over-tuned, there will be a large difference between the genome assembly results of AutoHiC and the T2T genome, and additional structural variations will be found after the whole-genome comparison between them.

We also constructed global dot plots to compare the AutoHiC correction results with the T2T reference genome (Figure 5C and D). From the dot plot, AutoHiC connects fragments on the same chromosome, reducing the number of scaffolds to improve consistency with the T2T reference genome. In addition, to determine the details of the adjusted genome, we compared the *H. sapiens* genome with the T2T reference genome by synteny analysis. Based on the structural error information detected by MUM&Co, we selected three types of corresponding error sequences and constructed a block collinearity map. Regarding translocation errors (Figure 5E), the collinearity map shows that, using the T2T genome as a reference, the region where the translocation error occurred was where the sequence had obvious placement errors. However, after AutoHiC adjustment, this issue was eliminated, the position of the sequence returned to normal, and there was a better collinearity result in the same region. Regarding inversion errors (Figure 5F), the regions where translocation errors occurred showed opposite sequence characteristics on the collinearity map, and after correction by AutoHiC, the collinearity with the T2T genome was more consistent. Additionally, there was a deletion error (Figure 5G). A collinearity map revealed that there was a fragment without collinearity in the original genome resulting from accidental insertion or other methods. Collinearity analysis indicated that in the genome adjusted by AutoHiC, the collinearity of this region was more consistent with that of the reference T2T genome. Additionally, we examined the contact heatmaps before and after the application of AutoHiC (Supplementary Figure S16), discovering that AutoHiC efficiently eliminates redundant sequences within the genome.

In conclusion, AutoHiC improved the accuracy of the genome assembly. We anticipate that when the AutoHiC model is integrated with other genome assembly software packages, it will also effectively address Hi-C assembly errors.

### Extending AutoHiC to complex genomes

To comprehensively assess the performance of AutoHiC and showcase its versatility, we applied the tool to a diverse set of complex genomes (Supplementary Table S8), including exceptionally large genomes such as *Schistocerca americana* (9 Gb), those with a substantial number of chromosomes such as *Chiloscyllium punctatum* (2*n* = 104), instances of polyploidy in plants, as exemplified by *Arachis hypogaea* (tetraploid) and three long-read sequenced genomes (*Pholis gunnellus*, *Taurulus bubalis* and *Thunnus maccoyii*).

To assess genome contiguity, we calculated the N50, L50 and CC ratio (Supplementary Figures S17–19) and observed a significant improvement in genome contiguity after AutoHiC correction. Moreover, we developed an independent error rate model to visualize the correction effect. Typically, translocation and inversion errors disappear after one round of correction (Supplementary Figure S20). The error rate was computed based on the length of the error relative to the genome size. As a result, its value exhibits minimal fluctuation; however, a discernible downward trend is evident.

**Figure 6.**
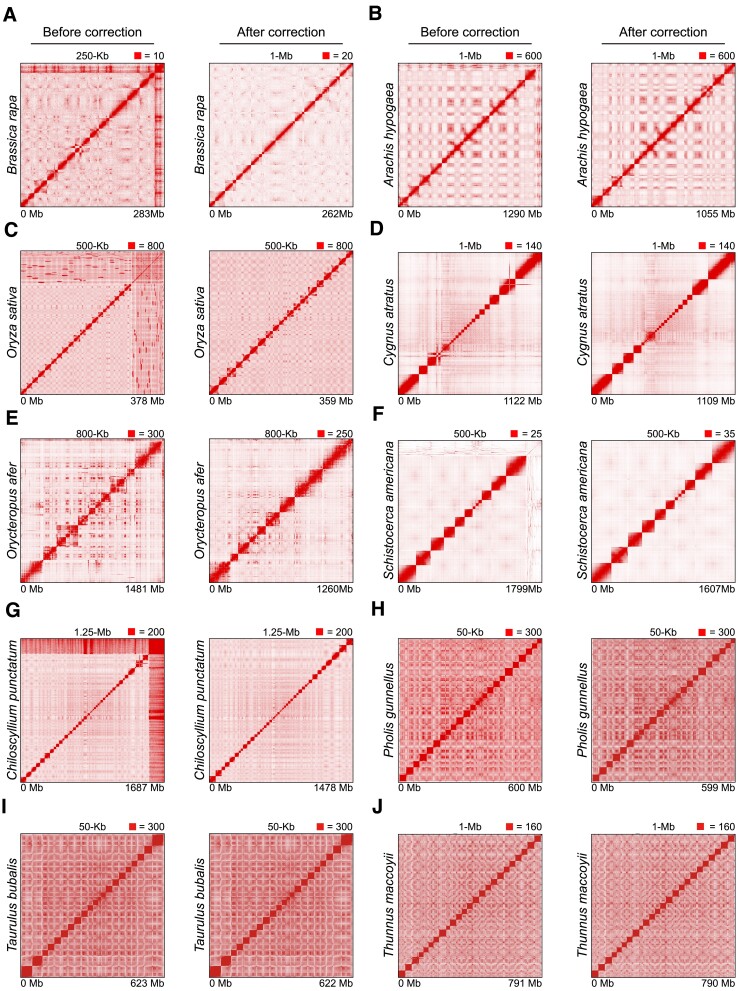
Genomic dataset extension validation. (**A–J**) Global contact heatmaps before and after AutoHiC error correction. The heatmap on the left shows the results before correction. The heatmap on the right has been corrected. The resolution and contact thresholds are marked at the top. The lower the value is the genome length.

To compare the changes before and after processing, Hi-C contact heatmaps of the ten species before and after correction are shown (Figure 6A–J). The assembly errors observed in the Hi-C contact heatmap prior to correction were eliminated, and redundant sequences were removed. Additionally, some contact heatmaps were compared before and after error correction to clearly illustrate the detailed changes (Supplementary Figure S21).

The results above demonstrate that AutoHiC significantly improves both contiguity and accuracy in terms of genome assembly by correcting genomic misassemblies. In summary, we have shown the effectiveness of AutoHiC when applied to complex genomic datasets and highlighted its broad applicability.

## Discussion

In this study, we present an innovative deep learning-based method called AutoHiC, which is designed to identify and correct misassembled contigs within assemblies. We introduce the ability to improve genome contiguity and accuracy across diverse datasets with varying complexities. AutoHiC stands apart from prior methodologies in two key aspects. First, the traditional assembly method (61–64) uses only the relationship between the contact matrix and the genome sequence for assembly, but AutoHiC converts the 2D contact matrix into a 3D contact heatmap to detect and correct assembly errors. A genome can be assembled more comprehensively and accurately, compensating for certain shortcomings of traditional methods. For example, 3D Hi-C contact data can reflect not only the strength of contact between adjacent fragments but also the contact patterns around them. This information can be used in genome assembly to correct structural errors, which is not possible with traditional methods. Second, the AutoHiC assembly pipeline operates in a fully automated manner (Figure 1A); neither error correction nor chromosome identification (Figure 2) necessitates manual intervention—only the provision of genome and Hi-C data. By optimizing the use of computational resources, AutoHiC utilizes the multithreading capabilities of both the CPU and GPU to deliver results swiftly. Additionally, AutoHiC generates detailed reports for each assembled genome, offering significant value for subsequent analyses.

Although AutoHiC has the above advantages, it also has shortcomings. For instance, in cases of complex genomes with a large number of repeated fragments and high heterozygosity, as well as low genome quality and problematic Hi-C sequencing data, AutoHiC may require multiple rounds of iterations to correct all errors.

Of all errors, debris is usually the most recognized error, but whether debris is a real assembly error is debatable. In the context of Hi-C contact heatmaps, debris is identified in regions exhibiting minimal or absent signals, which can be attributed to factors such as highly repetitive sequences, insufficient sequencing depth, technical noise and structural variations within the genome. These areas are typically represented by white or light-colored zones, indicating negligible or non-existent genomic contacts. Although there is no definitive proof linking debris formation to assembly errors, our analysis approached the phenomenon from multiple angles (Supplementary Text S3). The origins of debris are multifaceted, making it challenging to ascertain whether it represents an authentic erroneous fragment through contact heatmap analysis alone. The most recent update of AutoHiC defaults to not adjusting debris, allowing users the flexibility to modify this setting in the configuration file. For the purpose of debris correction, the error correction module segregates the scaffold into three segments, relegating the debris segment to the genome’s extremity to serve as a surplus sequence.

In addition, although AutoHiC improves the accuracy of genome assembly, it has encountered challenges in the accuracy of genome single bases, especially at sequence-adjusted sites. Therefore, generating a single-base contact matrix from the sequencing data is not feasible. This limitation affects AutoHiC’s ability to detect and correct errors, as it relies on contact matrices. To determine the extent of the effect, we used the single-base genome quality assessment tool, CRAQ, to compare AutoHiC with other genome assembly software (Supplementary Text S6). Our results indicate that while AutoHiC cannot correct at the single-base level, it is equally effective as other software. Furthermore, we investigated the impact of deep learning-based corrections versus manual corrections on genome quality. Specifically, we focused on the correction of translocation and inversion errors. Our results demonstrate that AutoHiC’s error correction improves genome quality more efficiently than manual adjustments performed with Juicebox.

With the increasing availability of high-quality Hi-C datasets, we hope that AutoHiC will help users obtain better chromosome-level genomes and liberate scientists from labor-intensive manual correction.

## Supplementary Material

gkae789_Supplemental_Files

## Data Availability

AutoHiC is available on GitHub at https://github.com/Jwindler/AutoHiC under an MIT license. All scripts and code used in this study are available in the attachment. All the datasets utilized in this study were obtained from publicly available databases. The Hi-C data can be downloaded from the DNA Zoo website (https://www.dnazoo.org). The genome and Hi-C read data can be downloaded from NCBI. Unpublished genome assemblies and sequencing data were obtained with permission from the DNA Zoo Consortium (dnazoo.org). The training dataset was generated using the AutoHiC script. All the data used in this article are available in Supplementary Tables S9 and S10. Additionally, the AutoHiC source code used in this study is also accessible on Zenodo (https://zenodo.org/records/10893906) and Figshare (https://doi.org/10.6084/m9.figshare.26117572).
