# Supplementary material for: A deep learning-based method enables the automatic and accurate assembly of chromosome-level genomes
Source: Nucleic Acids Res. 2024 Sep 17;52(19):e92. doi: 10.1093/nar/gkae789 (PMC11514472; doi:10.1093/nar/gkae789)
Supplement: gkae789_Supplemental_Files [file gkae789_supplemental_files.zip › Supplemental Information.pdf]

Supplementary Text

Text S1. Definition and Correction of Translocation

Definition: "Translocation" errors are common chromosomal translocation errors in genome assemblies and are characterized by cross-like regions observed in Hi-C contact heatmaps. The center of this area has contact characteristics, and the surrounding areas have basically no contact. This pattern reflects unexpectedly high levels of Hi-C contact (blue area) between two sequences that are not in close 1D proximity in the reference genome assembly.

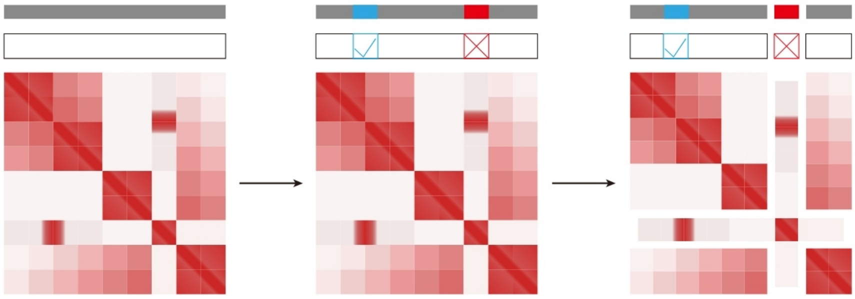

Translocation correction example. The image on the left displays a translocation on the contact heatmap. The position of the translocation on the contact heatmap is marked by the red X in the middle figure. The right image shows the contact heatmap and the sequence after the translocation cutting. The gray line above represents the scaffold.

Correction: To correct translocation errors, translocation fragments were removed. The objective of the repair process is to align the order of draft scaffolds with the actual chromosomal layout. The AutoHiC error detection module can identify the region where the translocation occurs (red square with X), and the peak algorithm calculates the correct insertion position (blue square) based on the location of the error. The error correction module then adjusts the sequence to correct the errors.

Text S2. Definition and Correction of Inversion

Definition: "Inversion" in a genome assembly denotes an error where the sequence orientation of a single scaffold or a group of adjacent scaffolds is erroneously reversed. In contact heatmaps, this phenomenon often manifests as a bowtie enrichment contact parallel to the diagonal.

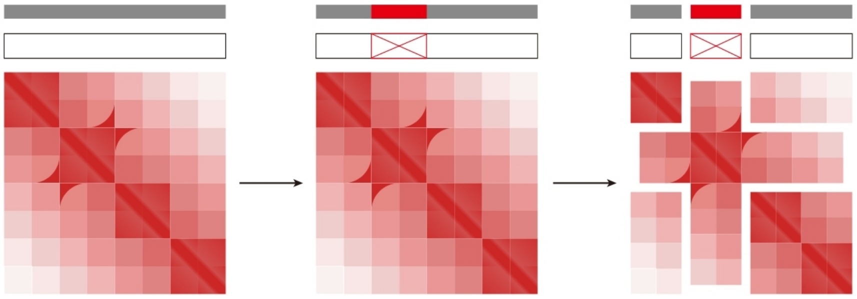

Inversion correction example. The left image displays the characteristics of the inversion on the contact heatmap. The position of the inversion on the contact heatmap is marked by the red X in the middle figure. The image on the right shows the contact heatmap and the sequence after inversion cutting. The gray line above represents the scaffold.

Correction: The objective was to correct the error by selecting and reversing the orientation of the scaffold sequences to adjust the genome assembly. The AutoHiC error detection module can identify the regions where inversions occur by analyzing contact heatmaps. The error correction module divides the scaffold fragment into three parts: two fragments are divided into two normal contact fragments and an inversion fragment (red square with X). The inversion fragment is then flipped to correct the error.

Text S3. Definition of Debris

Definition: Regions with sparse or zero signals in the Hi-C contact heatmap are defined as "Debris". A contact heatmap typically displays white or light-colored areas to indicate little or no contact, respectively.

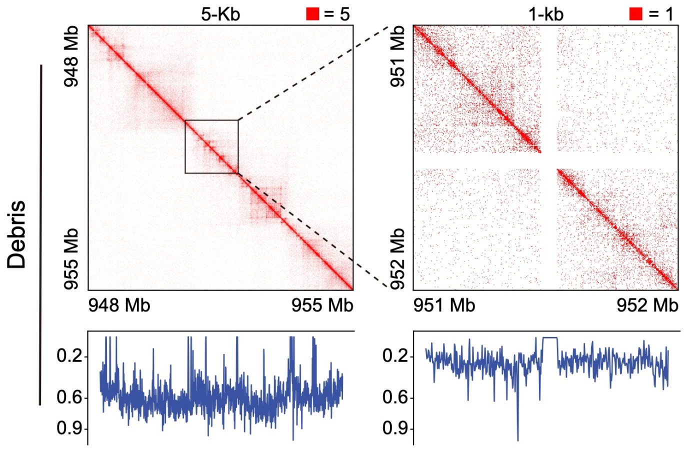

Debris example. The left image displays the characteristics of the debris as depicted on the contact heatmap, while the right image presents the high-resolution debris contact heatmap. Below, the contact curve is normalized to a range of 0-1.

These regions may be characterized by highly repetitive sequences, low sequencing depth, sparse contact frequencies, technical noise, and genomic structural attributes. Insufficient sequencing depth in Hi-C experiments can lead to inadequate detection of contact frequencies in certain genomic regions, resulting in small or zero values in corresponding elements of the Hi-C contact matrix. Under certain cellular conditions or states, certain genomic regions may exhibit lower contact frequencies with others. Elements associated with these regions in the contact matrix may have small values or zeros, reflecting the sparse nature of contact. Technical noise during experimental procedures or sequencing can introduce inaccuracies in capturing certain contact signals, which may result in small or zero values in specific elements of the Hi-C contact matrix. The presence of certain genomic regions may result in lower contact frequencies due to their structural features or chromatin states. This can be reflected in the corresponding elements of the Hi-C matrix, which may exhibit small values or zeros.

Text S4. Description of anomalous software assembly results.

A comparison of the software data revealed that the N50 of Pin_hic (1) and YaHS (2) were very large and were close to the genome size; therefore, we carefully studied their assembly results. We generated a histogram of the genome size and length of the top n sequences (n is the number of chromosomes) in the software assembly results. It is obviously wrong to say that YaHS and Pin_hic merge multiple chromosomes together. To avoid misunderstanding, their results are shown in the appendix. Additionally, we conducted a thorough review of the research articles by YaHS and Pin_hic. Our aim was to rectify the results by adjusting the parameters of both the YaHS and Pin_hic tools for the *Arabidopsis thaliana* genome assembly. However, we observed only minimal improvement in the N50 length of the scaffolds.

YaHS:

In each round, YaHS constructs a contact matrix by dividing each contig into chunks of a specific size (resolution) and assigns Hi-C contact signals to cells of chunk pairs. To improve the results, we attempted to adjust the resolution by increasing or decreasing the chunk size. We tested the default parameters, increasing (-r 5000,10000,20000,50000,100000,200000,500000,1000000,2000000,5000000,10000000,20000000)) and decreasing (-r 50000,100000,200000,500000,1000000,2000000,5000000,10000000,20000000) the chunk size.

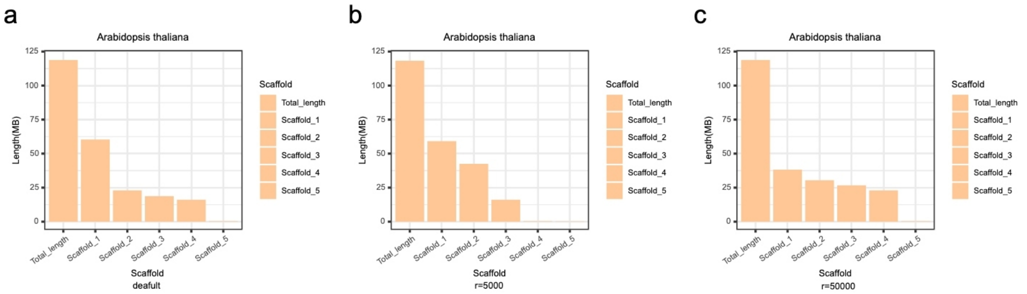

YaHS parameter survey diagram. The same data and separate parameters were used for each plot. The parameters are labeled below the graph.

Unfortunately, based on the aforementioned results, this approach does not appear to be effective. The largest scaffold still contained two separate scaffolds.

Pin_hic:

Pin_hic iteratively pursues the generation of chromosome-level scaffolds through two steps: Step A involves building a contact matrix by counting linking read pairs between contig ends based on BAM files and, if available, the SAT file. Step B constructs a scaffolding graph

based on the N-best neighbors of contigs using the contact matrix, performs joins, and outputs results in the 'SAT' format. We increase the default iteration count from 3 to 5 and 10:

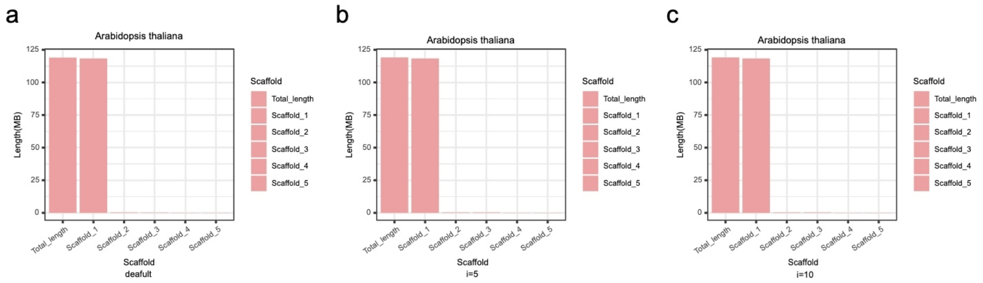

Pin_hic parameter survey diagram. The same data and separate parameters were used for each plot. The parameters are labeled below the graph.

Despite increasing the default iteration count from 3 to 5 and 10, there was no noticeable improvement in the quality of the scaffolds. During our investigation of Pin_hic, we discovered a similar issue on GitHub (https://github.com/dfguan/pin_hic/issues/5). However, the author's solution involves manual separation, which may not be ideal. In summary, we cannot obtain accurate results by adjusting the parameters.

Text S5. Model training data sources and quantity

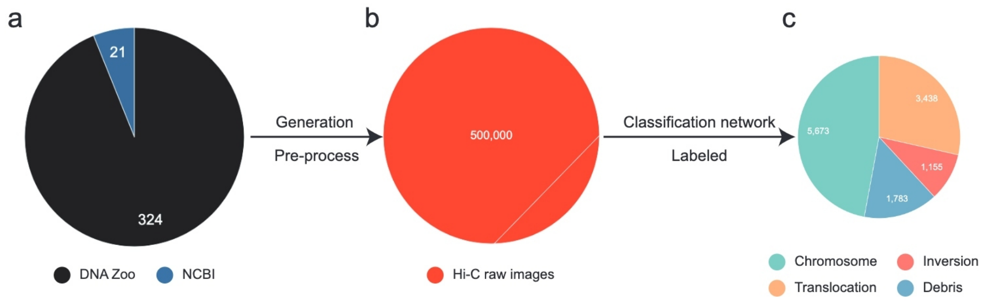

The data sets utilized for AutoHiC model training were derived from two distinct databases: DNA Zoo and NCBI. The Hi-C contact matrix of 324 species was obtained from DNA Zoo, while the Hi-C contact matrix of 21 species was constructed from the original Hi-C sequencing data sourced from NCBI. A total of approximately 500,000 contact heatmaps were generated using the Hi-C contact matrices of these species. Four types were selected for training and testing of the final model: chromosome, translocation, inversion, and debris. These datasets were not augmented with validation data, and the training process employed cross-species data for cross-training and testing.

Text S6. Comparison of single bases with various software programs.

Given the absence of manual refinement tools capable of achieving single-base resolution, we aimed to investigate the impact of this limitation on genome assembly. Clipping Information for Revealing Assembly Quality (CRAQ) is a novel reference-free tool that maps raw reads back to assembled sequences, identifying regional and structural assembly errors through effective clipped alignment information. It transforms error counts into corresponding assembly evaluation indices, reflecting assembly quality at single-nucleotide resolution.

We employ CRAQ (3) to compute both R-AQI and S-AQI by analyzing the assembly outcomes from various software programs. The comparison of these results provides insights into the quality of the assembly results.

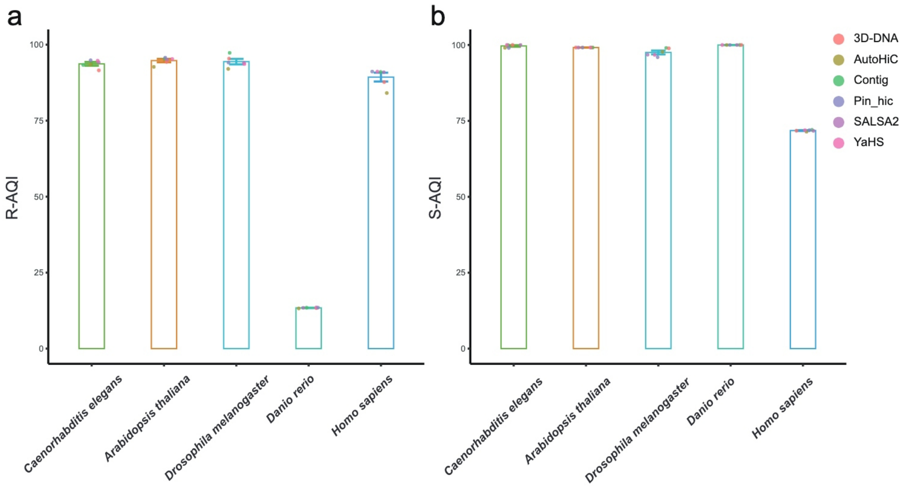

CRAQ measurements of five model species. The results of the R-AQI metric are presented on the left. On the right is the S-AQI. The dot colors represent the various software packages.

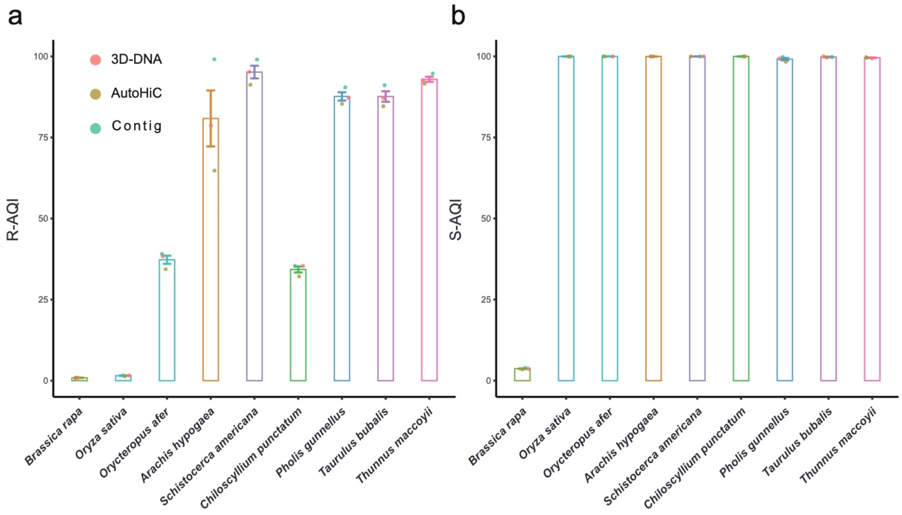

CRAQ measurements of the test species. The results of the R-AQI metric are presented on the left. On the right is the S-AQI. The dot colors represent the various software packages.

The CRAQ results indicate that compared to other tools, AutoHiC does not significantly improve genome quality in single base resolution. This lack of improvement is primarily due to the resolution of the Hi-C contact images not reaching single-base resolution. Manual or artificial intelligence (AI) adjustments are inevitable. In addition, AutoHiC call 3D-DNA (4), which uses a relatively aggressive correction strategy that results in numerous fragmentations, negatively affects the CRAQ metrics. Consequently, the single-base evaluation method does not accurately reflect the genome assembly capabilities of AutoHiC. However, according to the CRAQ's evaluation, the performance of AutoHiC matches or exceeds that of other software.

To thoroughly assess the influence of different error types on the CRAQ evaluation metrics before and after correction, we employed the CRAQ to evaluate the changes in individual errors by using AutoHiC and manual refinement (Juicebox) (5). We confirmed instances of translocation and inversion by selecting three examples of each, based on available data. Initially, we analyzed the genome with CRAQ prior to correction, followed by post-correction assessment for individual errors. Given the variable number of errors present in a genome, it may be necessary to test each error across multiple genomes.

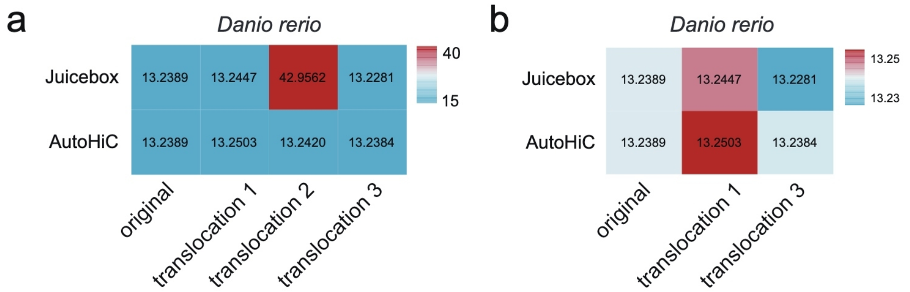

CRAQ measurements of translocations. CRAQ results of translocation error correction. The numerical values represent the R-AQI values. Species names are marked above the heatmap.

Translocation errors were confirmed in *D. rerio*. Correction of three errors resulted in an improvement in the R-AQI in two instances and a decrease in one. Further analysis revealed that the abnormal increase in R-AQI was attributed to translocation-2, which was subsequently removed. AutoHiC consistently outperforms manual refinement.

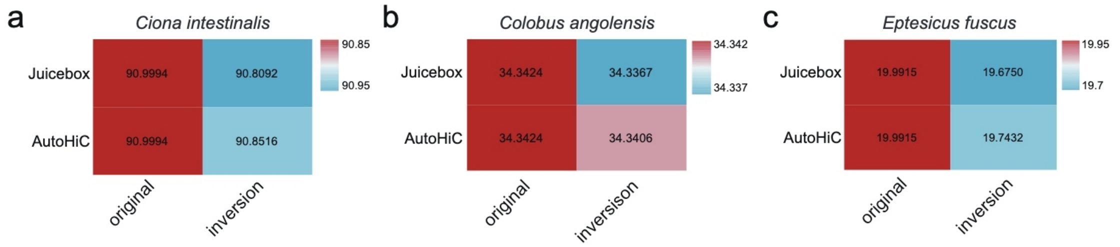

CRAQ inversion measurements. CRAQ results of inversion error correction. The numerical values represent the R-AQI values. Species names are marked above the heatmap.

The test, which targeted three species, was initiated due to the low incidence of inversion errors. The depicted results illustrate a reduction in R-AQI following manual application of Juicebox and AutoHiC. Nonetheless, the performance of AutoHiC was superior to that of manual refinement.

Compared to previous methods, AutoHiC has made improvements in resolution recognition, including the following steps: (1) Although both Juicebox and AutoHiC can detect and correct errors at different resolutions, AutoHiC iterates through contact heatmaps at each resolution and uses a filtering algorithm based on the location of each error. If the interval between two errors is less than the minimum resolution, they are considered to be the same error. In AutoHiC, errors with high resolution are selected as the result. The advantage of this approach is that false detections on high-resolution heatmaps have smaller errors relative to their locations in the genome. This is currently the optimal solution. However, implementing this approach on Juicebox can be challenging due to varying user screen sizes. When manually detecting errors at high resolutions, the contact heatmap may not be fully displayed, making it difficult to observe errors or allowing only partial observation. (2) The main task of the peak algorithm is to find insertion sites for translocation errors. One advantage of this method is its ability to accurately locate insertions. This differs from Juicebox, where insertion positions are manually determined on the basis of the color of the contact heatmap. The peak algorithm calculates the dimensions of the extraction matrix based on the error length and minimum resolution and then extracts the contact value of each row of the contact matrix to find the peak (i.e., the point with the highest contact value). The insertion site of the translocation can be identified by removing the peak with the most repeats, as verified in the manuscript. This approach utilizes the data characteristics of the contact matrix to reduce human errors. The insertion site was calculated based on the minimum resolution, ensuring that errors were minimized.

In summary, current Hi-C technology cannot construct contact matrices at the single-base level, which limits the ability of AutoHiC to detect and correct errors at this resolution. However, the CRAQ results indicate that the performance of AutoHiC is on par

with, if not superior to, that of other software, with its outcomes surpassing of manual refinement.

Supplementary Figures

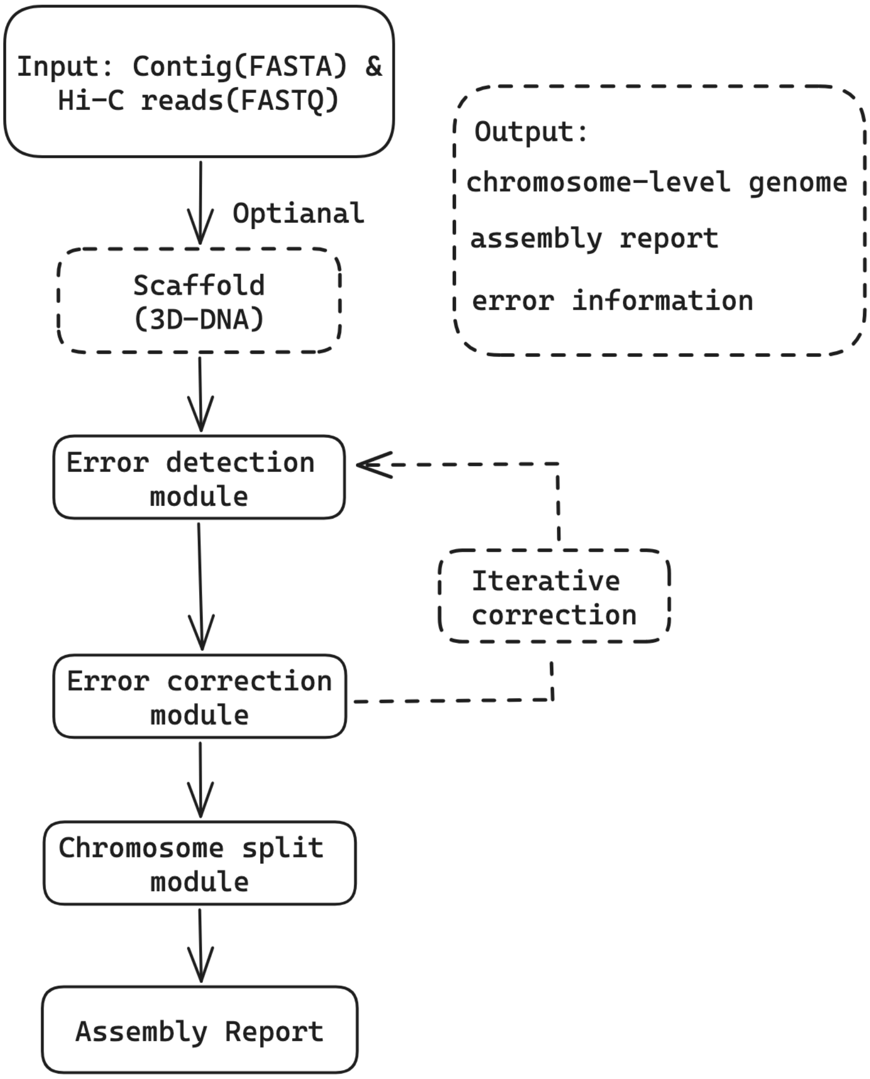

Figure S1. Schematic of the AutoHiC algorithm. AutoHiC takes the contig genome and Hi-C reads as input files and maps these reads to the assembly to obtain the contact matrix. The tool then identifies misjoins, translocations, inversions, and debris based on the contact heatmap. Additionally, AutoHiC provides an assembly report of the correction process, which helps users understand the correction process and genomic information. Moreover, users of scaffold software can choose the software they prefer. Error correction may require multiple iterations.

Summary

Species	Brassica rapa
Assembly size (bp)	262330601
Scaffold N50 (bp)	24553947
Scaffold N90 (bp)	19027318
CC ratio (%)	1.00
Structural errors ratio (%)	0
Number of chromosomes	11
HiC Anchor rate (%)	92.37
Number of scaffolds	11
Longest scaffold (bp)	34719908
Scaffold L50	5
Scaffold L90	10
GC (%)	35.09

Figure S2. AutoHiC Results Report Part I. The basic information of the genome was assembled by AutoHiC, including genome size, N50, CC ratio, anchor rate and GC content. This part is derived from the analysis of the assembly result report test, which is obtained through analysis of the same species.

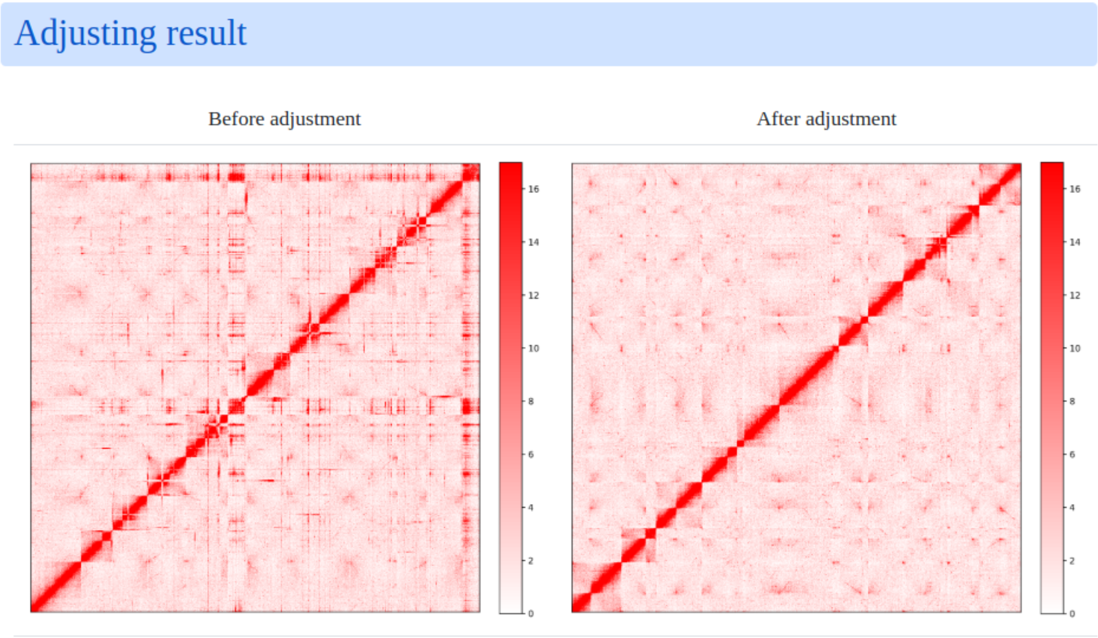

Figure S3. AutoHiC Results Report Part II. Comparison of global contact heatmaps before and after AutoHiC error correction. The left panel shows the data before adjustment. On the right, the model is adjusted. This part is derived from the analysis of the assembly result report test, which is obtained through analysis of the same species.

Error adjustment

Translocation

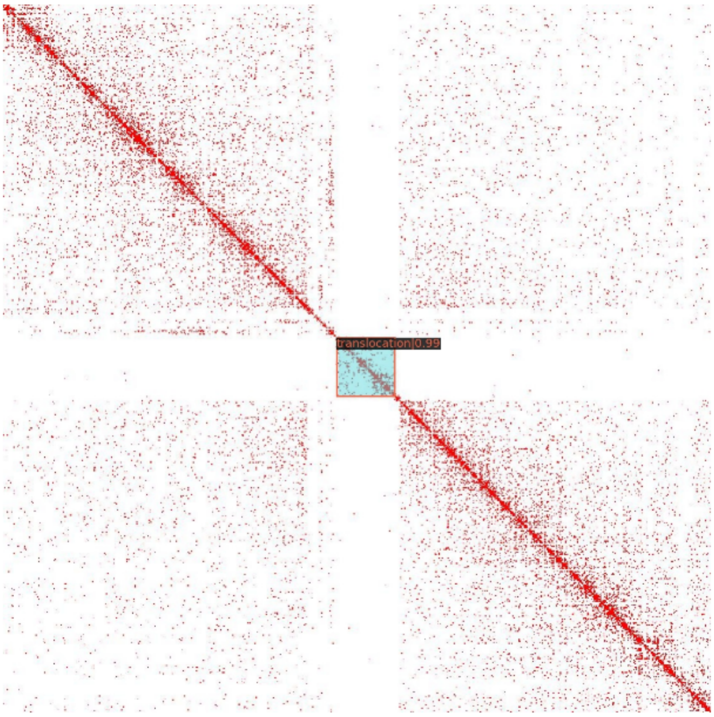

Location: 21648009-21934703

1

Figure S4. AutoHiC Results Report Part III. Error details were detected by AutoHiC, which contains error visualization and location information. The area marked by the box is the area where the error occurred. The specific locations where this occurs are listed below. This part is derived from the analysis of the assembly result report test, which is obtained through analysis of the same species.

Chromosome length

Molecule	Length(bp)	GC(%)
All	262330601	35.09
Chromosome 1	29096201	33.34
Chromosome 2	20030698	33.37
Chromosome 3	26915038	33.83
Chromosome 4	20539817	33.91
Chromosome 5	24553947	33.82
Chromosome 6	34719908	34.28
Chromosome 7	17287781	34.57
Chromosome 8	20321949	33.79
Chromosome 9	25536935	33.99
Chromosome 10	19027318	33.13
Chromosome 11	24301009	33.79

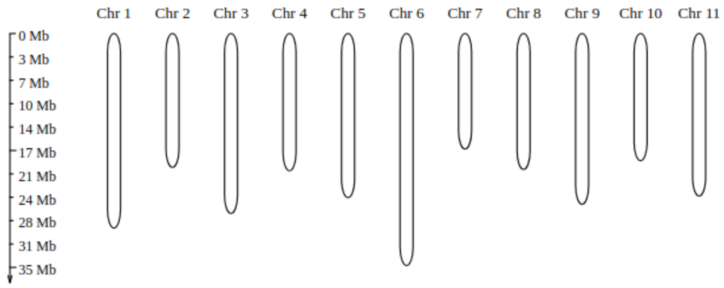

Figure S5. AutoHiC Results Report Part IV. The chromosome number and length were predicted by AutoHiC, and the GC content of each chromosome was calculated. A visualization of the chromosomes is shown below. This part is derived from the analysis of the assembly result report test, which is obtained through analysis of the same species.

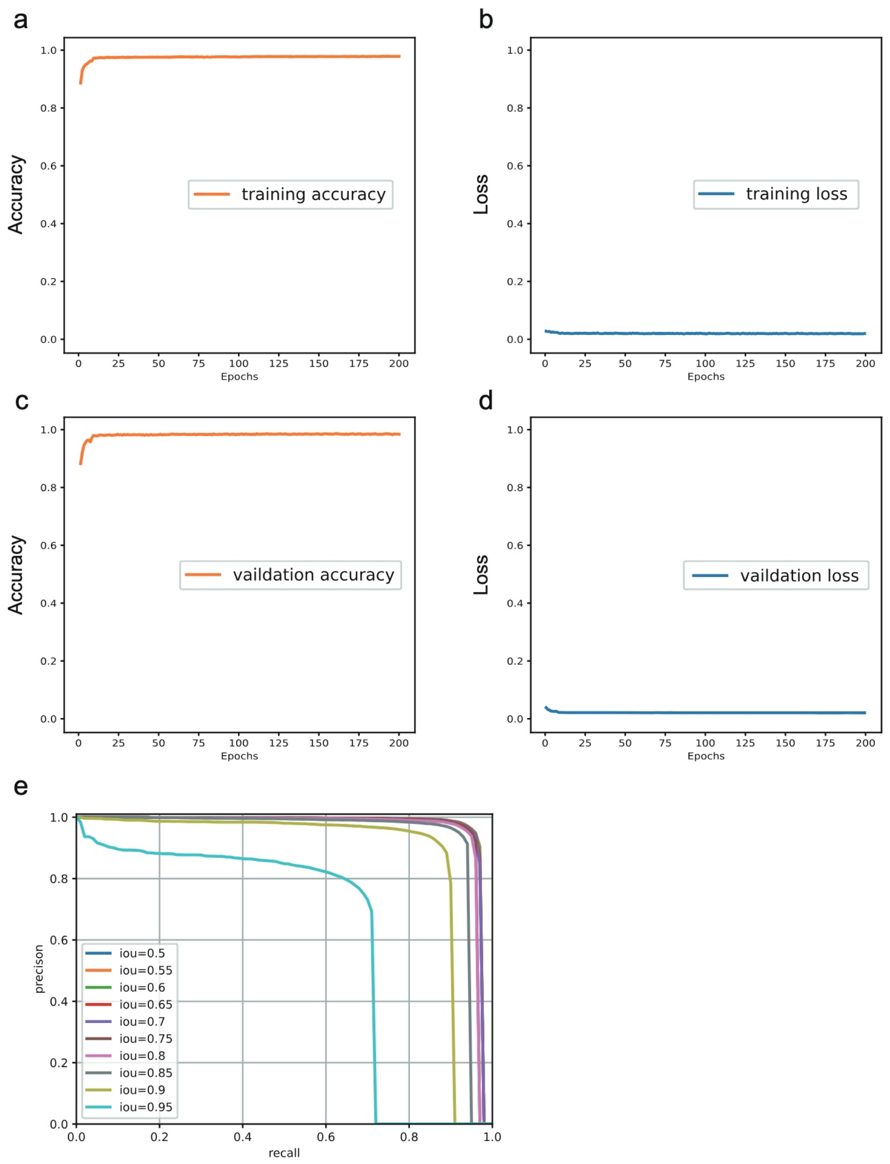

Figure S6. Chromosome identification model evaluation. a, **b, c, d** Changes in model accuracy and loss rate during training and validation. **e** Precision-recall (PR) curve for the different IOU thresholds shown in the panel.

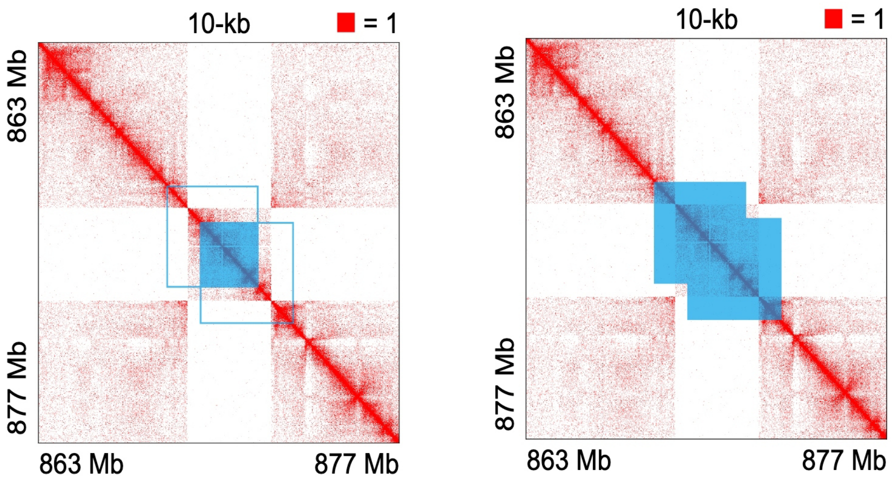

Figure S7. Intersection over Union calculation diagram. Calculation diagram of the intersection over union during error detection. The blue boxes represent candidate boxes that were falsely detected. The intersection of the two candidate boxes in the picture on the left represents the intersection area, while the union area of the two candidate boxes in the picture on the right represents the union area.

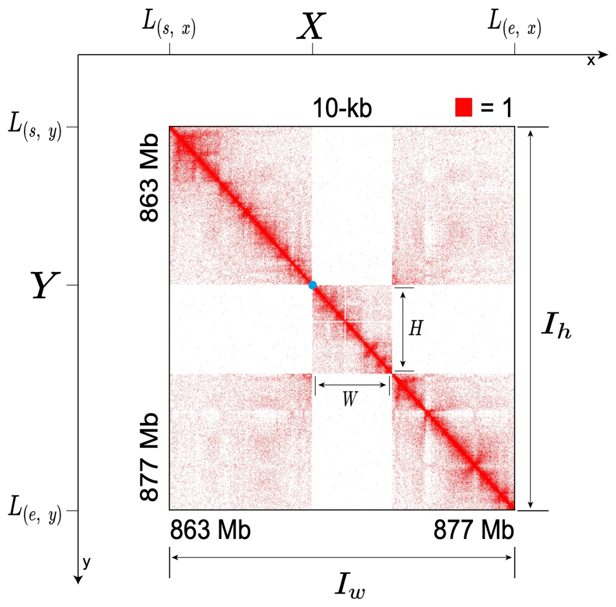

Figure S8. Error location conversion diagram. The error is located at the blue point in the upper left corner, with coordinate $[X,\ Y,\ W,\ H]$. X and Y represent the starting positions of the upper left corners of the error box on the x- and y-axes, respectively. W and H represent the width and length of the error box, respectively. The start and end positions of the contact image on the genome are represented by $L_{(s,x)}, L_{(e,x)}$ and $L_{(s,y)}, L_{(e,y)}$, respectively. I_h and I_w are the width and height of the contact images, respectively.

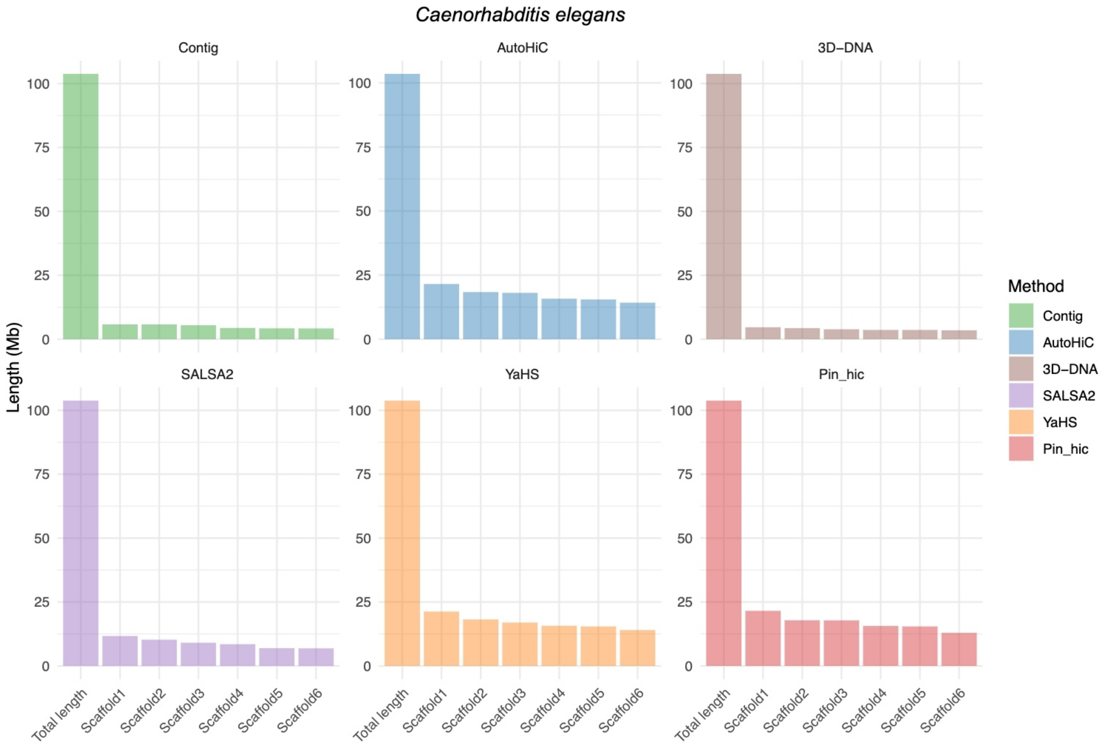

Figure S9. Genome and scaffold length of *Caenorhabditis elegans*. Only the genome length and the length of the first n scaffolds were counted, where n is the number of chromosomes. One panel per software.

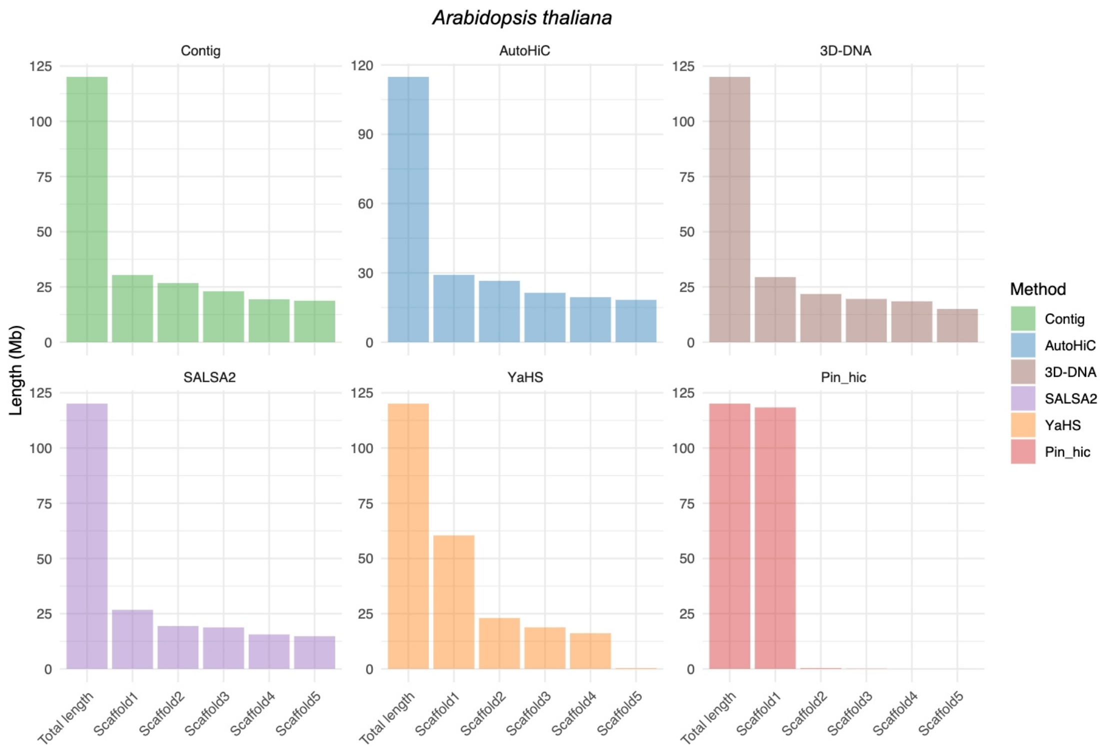

Figure S10. Genome and scaffold length of *Arabidopsis thaliana*. Only the genome length and the length of the first n scaffolds were counted, where n is the number of chromosomes. One panel per software.

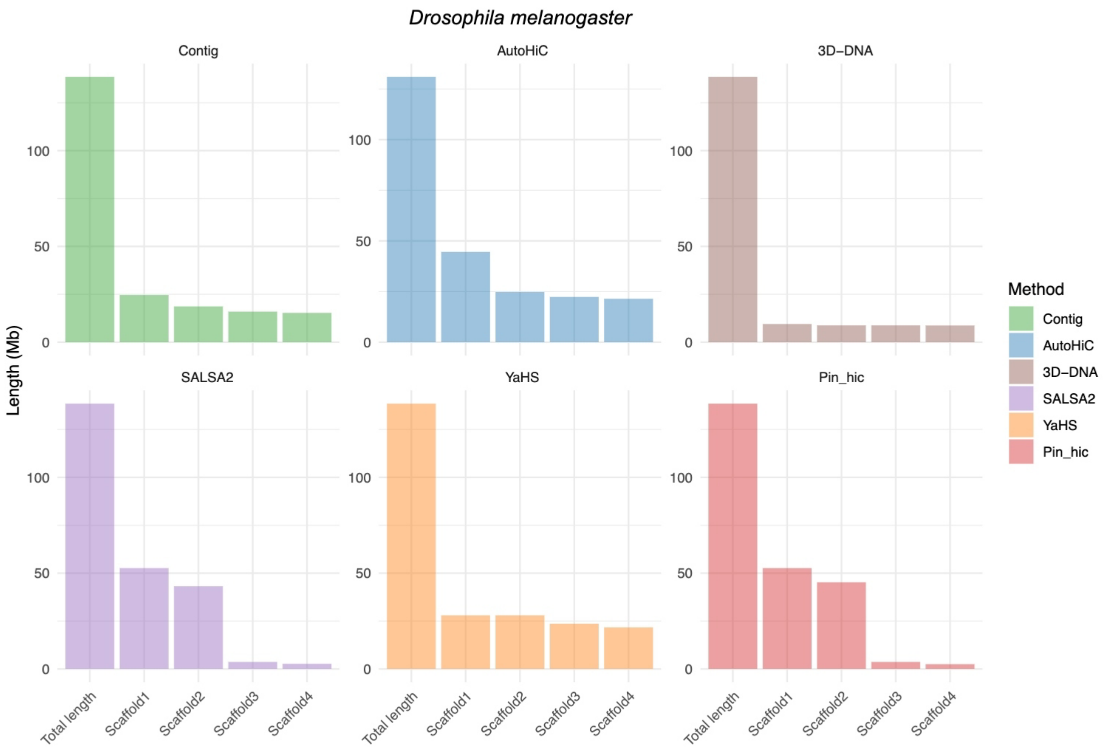

Figure S11. Genome and scaffold length of *Drosophila melanogaster*. Only the genome length and the length of the first n scaffolds were counted, where n is the number of chromosomes. One panel per software.

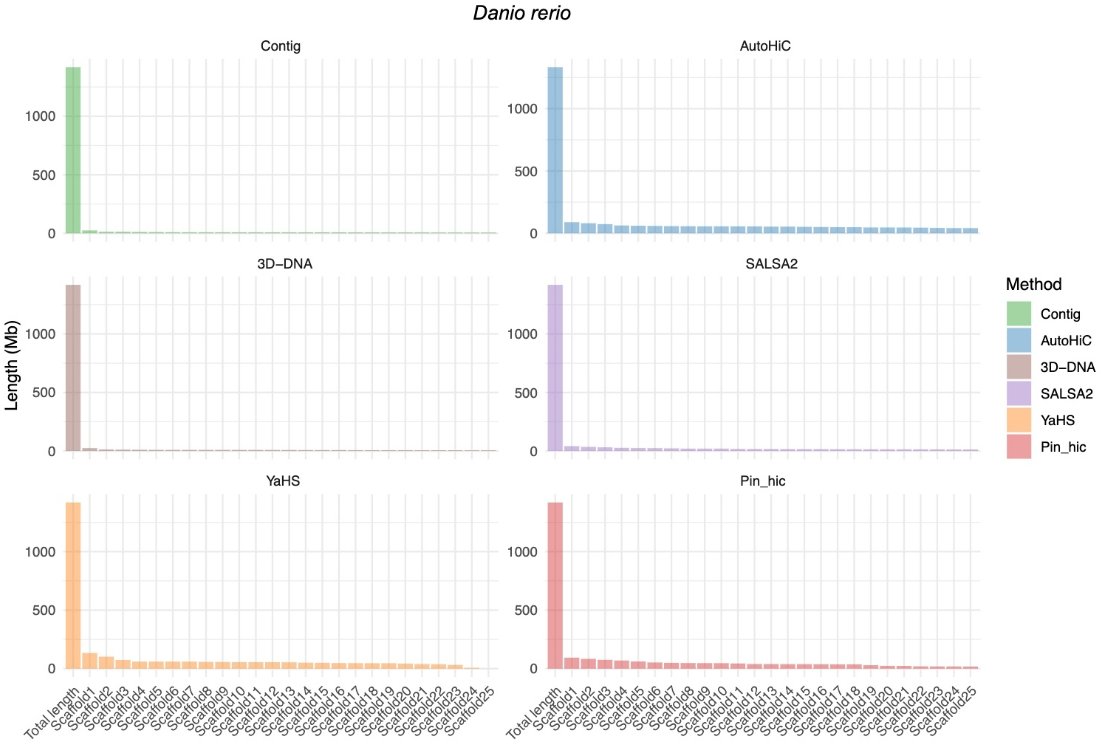

Figure S12. Genome and scaffold length of *Danio rerio*. Only the genome length and the length of the first n scaffolds were counted, where n is the number of chromosomes. One panel per software.

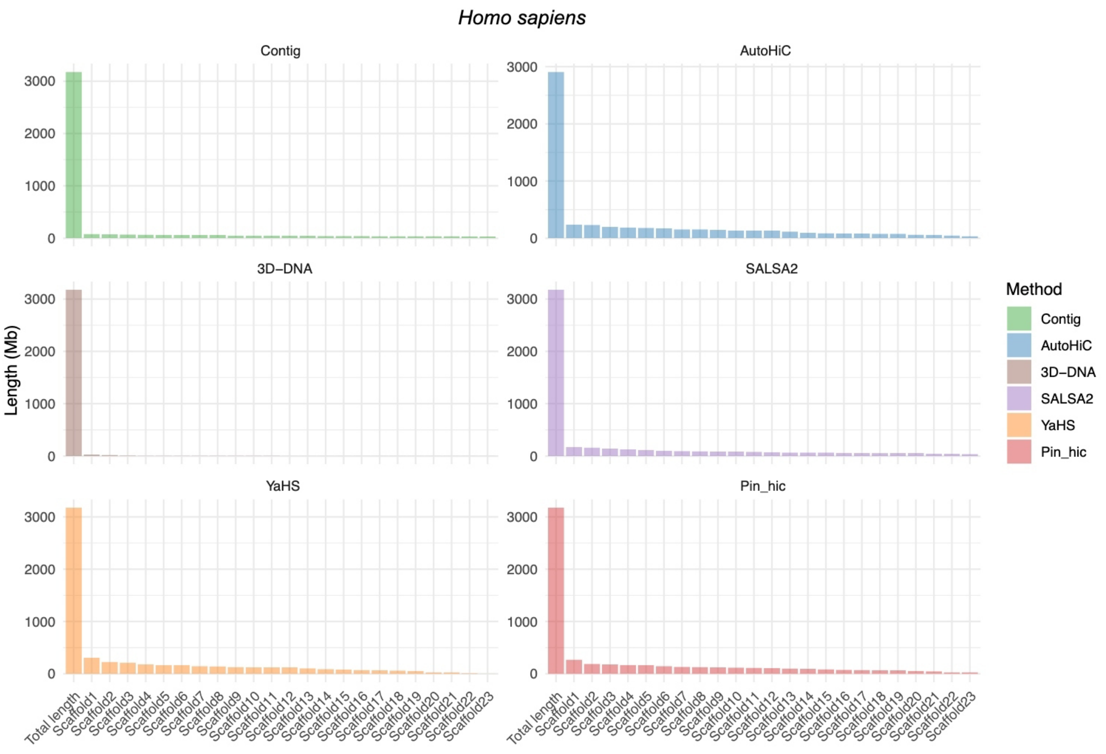

Figure S13. Genome and scaffold length of *Homo sapiens*. Only the genome length and the length of the first n scaffolds were counted, where n is the number of chromosomes. One panel per software.

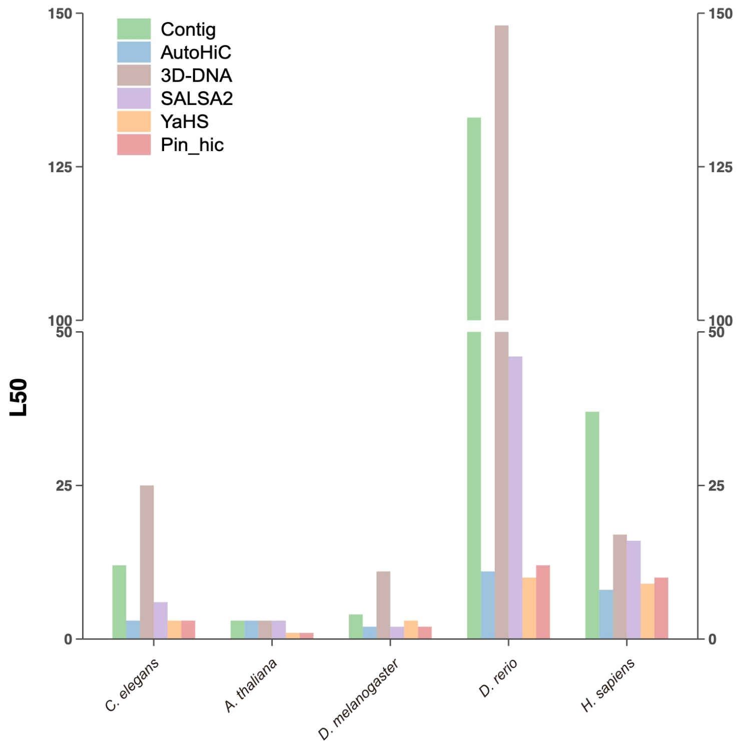

Figure S14. Performance evaluation of L50 on the five species. Histogram of L50 values for different software contig results. The figure contains contig results, and different colors represent different software packages.

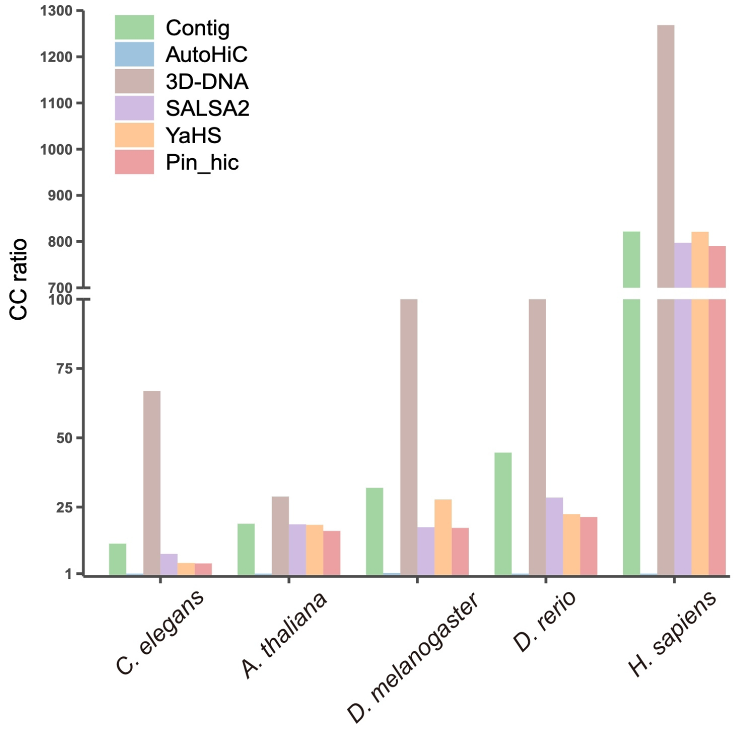

Figure S15. Histogram of CC ratio values of different software contig results. The results are presented for each contig, and different colors represent different software packages.

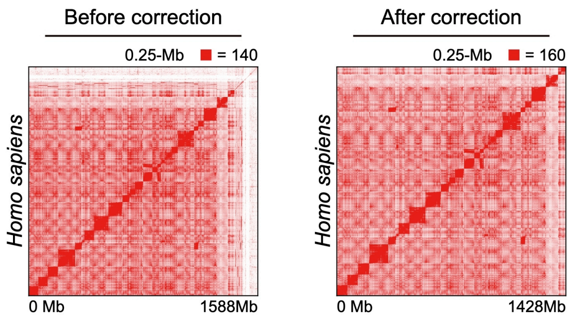

Figure S16. Contact heatmaps before and after AutoHiC correction were compared. Comparison of global contact heatmaps of *Homo sapiens* before and after correction. The left panel shows the data before adjustment. Right is after adjustment.

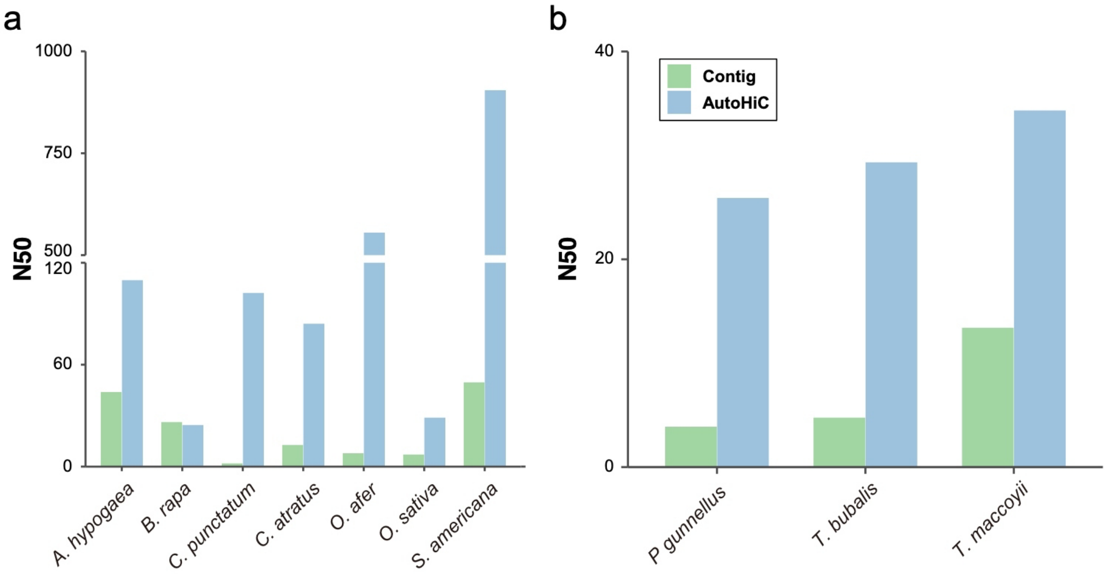

Figure S17. N50 histogram before and after genome assembly. Changes in N50 before and after genome assembly with AutoHiC. The Y-axis is the size of the N50, and the X-axis is the different genomes. The colors represent the genome before and after assembly.

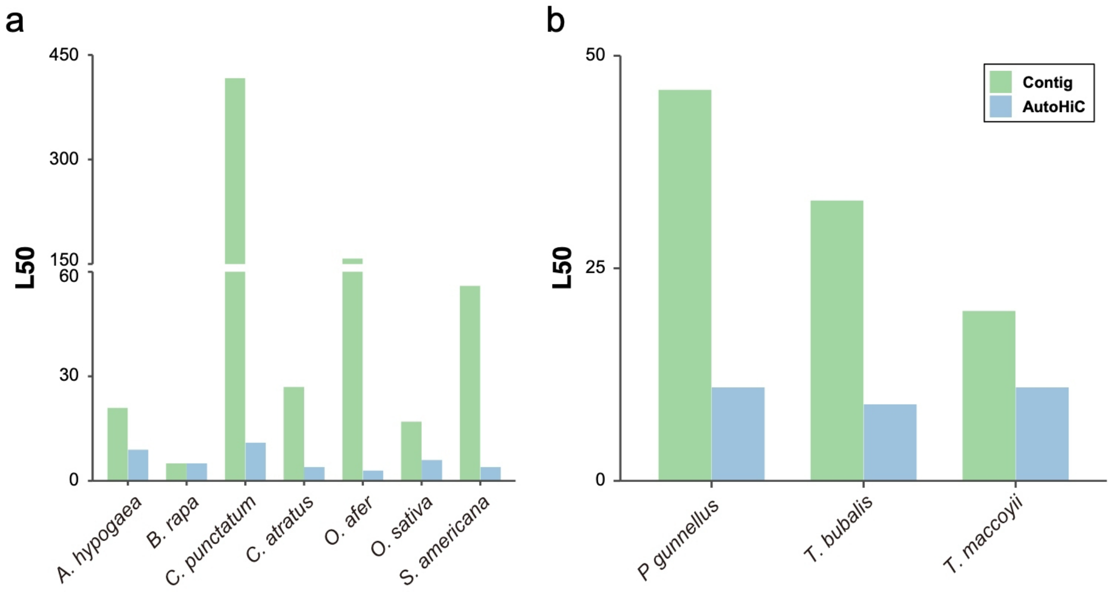

Figure S18. L50 histogram before and after genome assembly. Changes in L50 before and after genome assembly with AutoHiC. The Y-axis represents the size of the L50, and the X-axis represents the different genomes. The colors represent the genome before and after assembly.

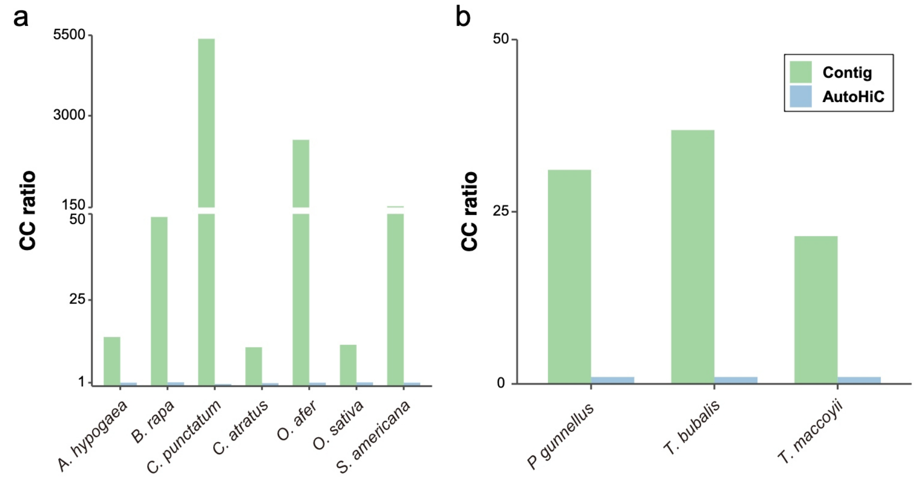

Figure S19. CC ratio histogram before and after genome assembly. Changes in the CC ratio before and after genome assembly with AutoHiC. The Y-axis is the size of the CC ratio, and the X-axis is the different genomes. The colors represent the genome before and after assembly.

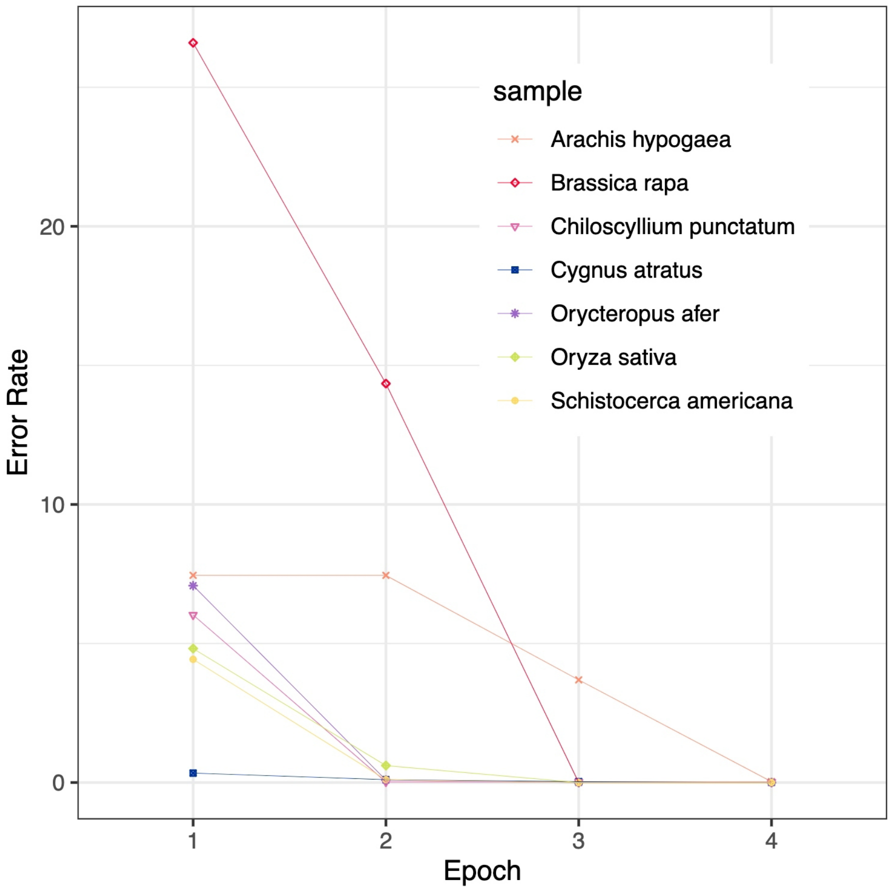

Figure S20. Error rate variation curve during genome assembly. Variation in the error rate for assembly errors corrected by AutoHiC. The X-axis is the number of iterative error corrections. The Y-axis is the magnitude of the error rate, scaled 1000-fold.

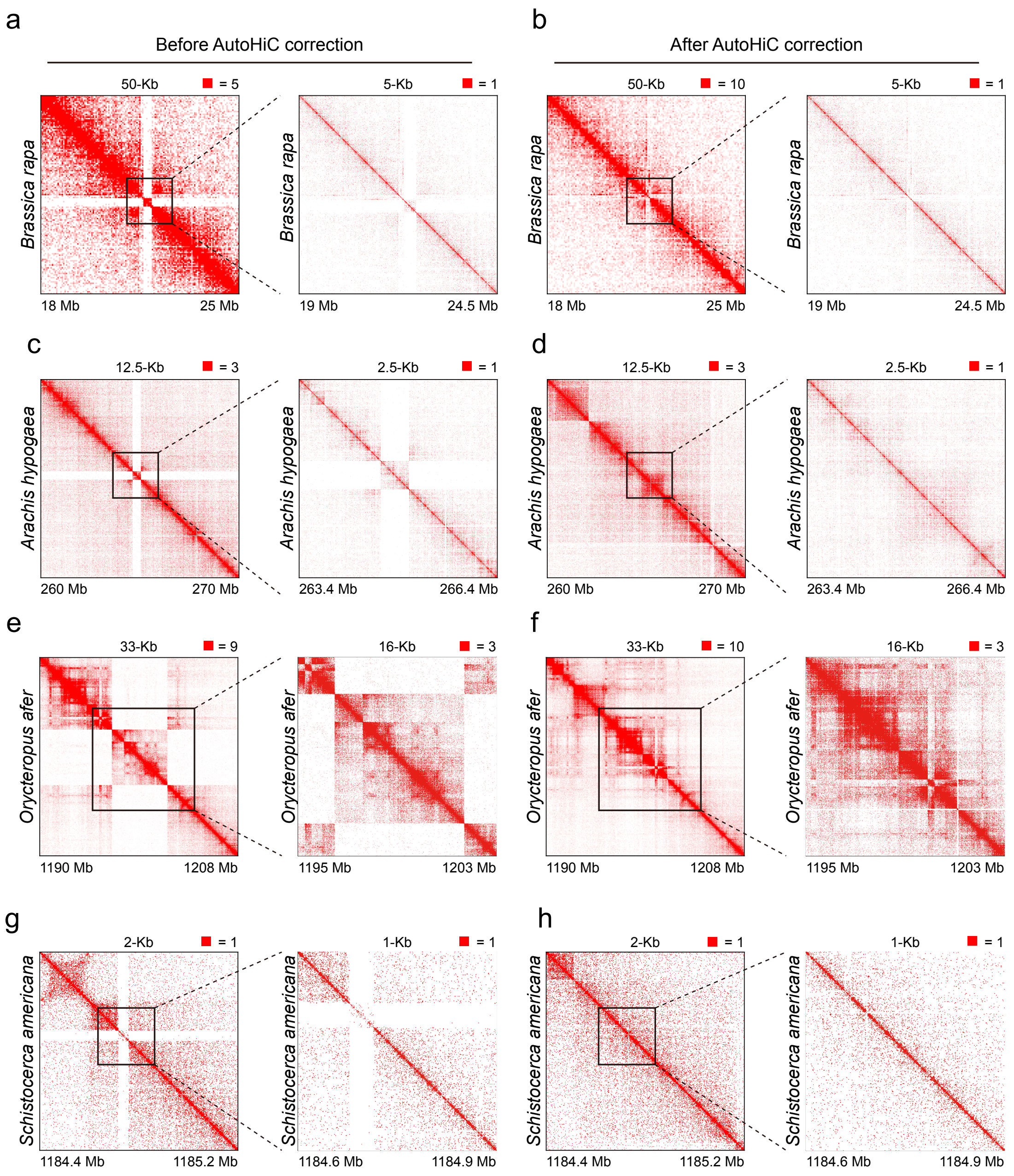

Figure S21. Contact heatmaps before and after AutoHiC correction. a, **b** Heatmap of contact before and after translocation error regions during correction in *Brassica rapa*. **c**, **d** Heatmap of contacts before and after translocation error regions were corrected in *Arachis hypogaea*. **e**, **f** Heatmap of contacts before and after translocation error regions were corrected in *Orycteropus afer*. **g**, **h** Heatmap of contacts before and after translocation error regions were corrected in *Schistocerca americana*. Low resolution is on the left, and high resolution is on the right.

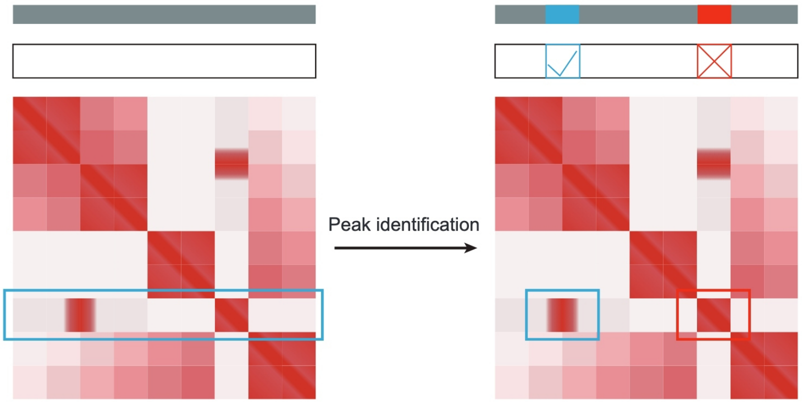

Figure S22. Peak algorithm diagram. In the left image, the blue box indicates the error matrix to be extracted. Its width is the full length of the contact matrix, and its height is the error length. The red box in the right image represents the area of error occurrence. The blue box is the insert site. The gray rectangle depicted above serves as a scaffold.

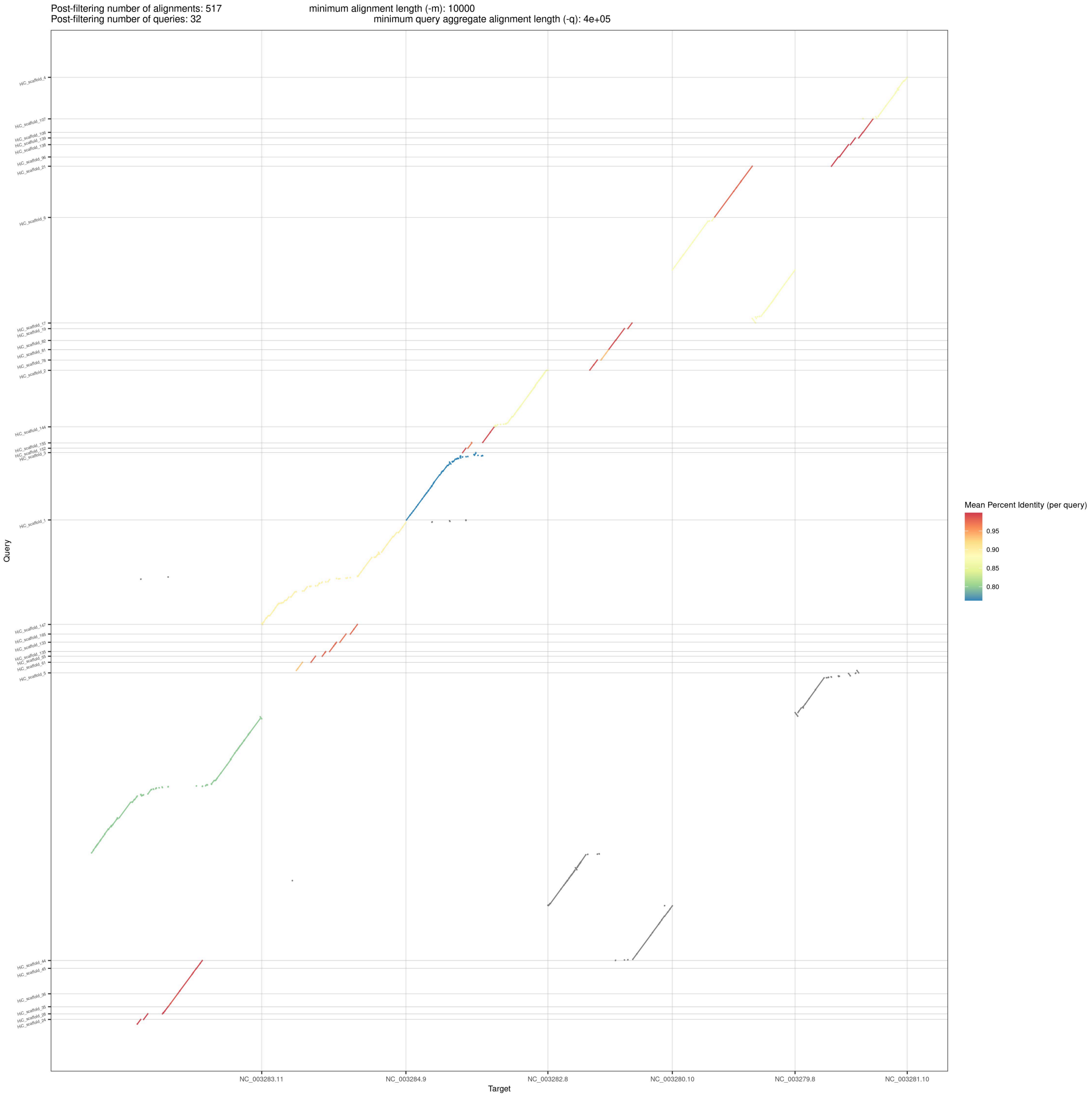

Figure S23. Dot plot of *Caenorhabditis elegans* for 3D-DNA. The horizontal axis represents the T2T reference genome, and the vertical axis represents the test genome. The colors indicate the degree of identity.

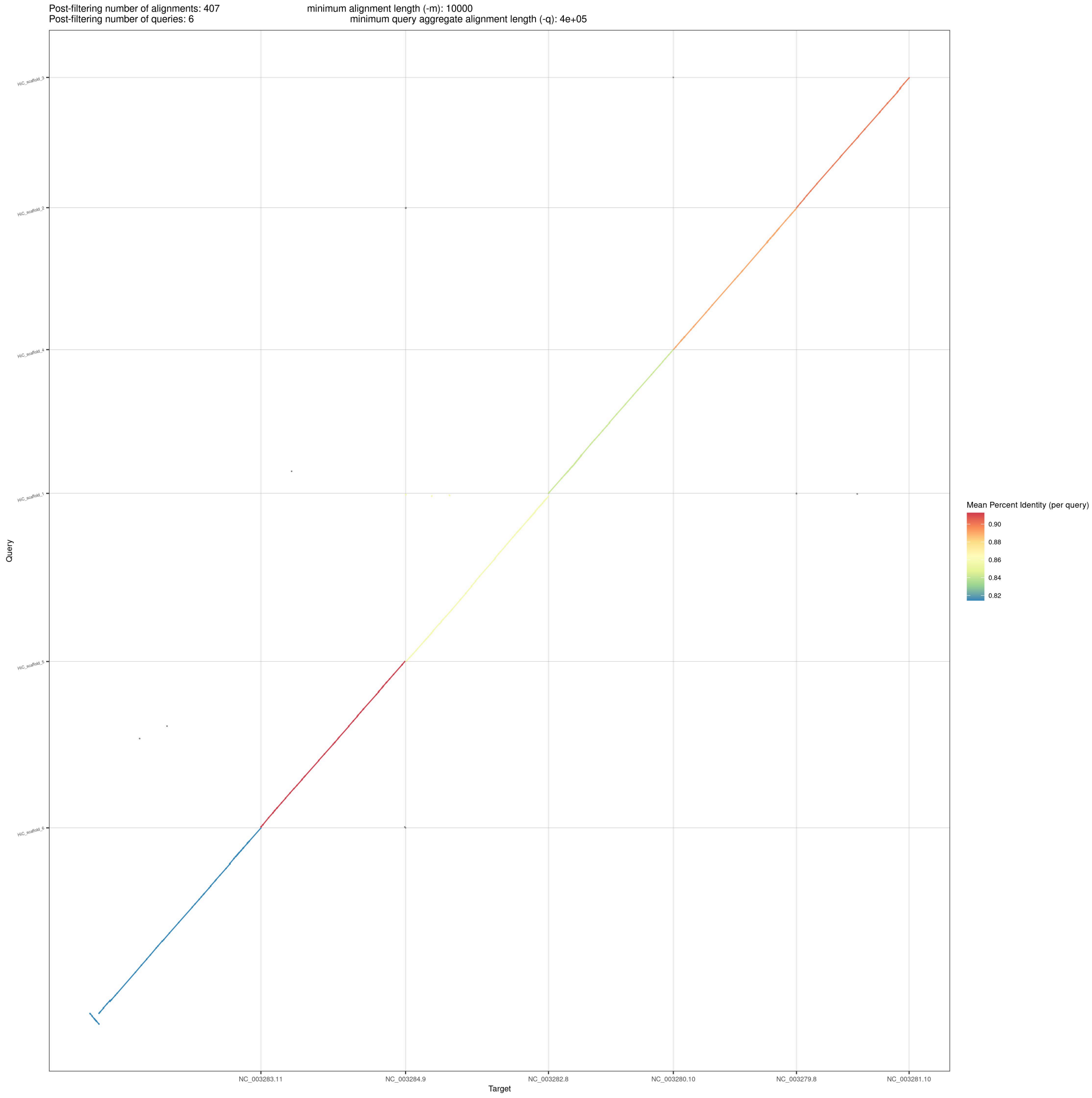

Figure S24. Dot plot of *Caenorhabditis elegans* for AutoHiC. The horizontal axis represents the T2T reference genome, and the vertical axis represents the test genome. The colors indicate the degree of identity.

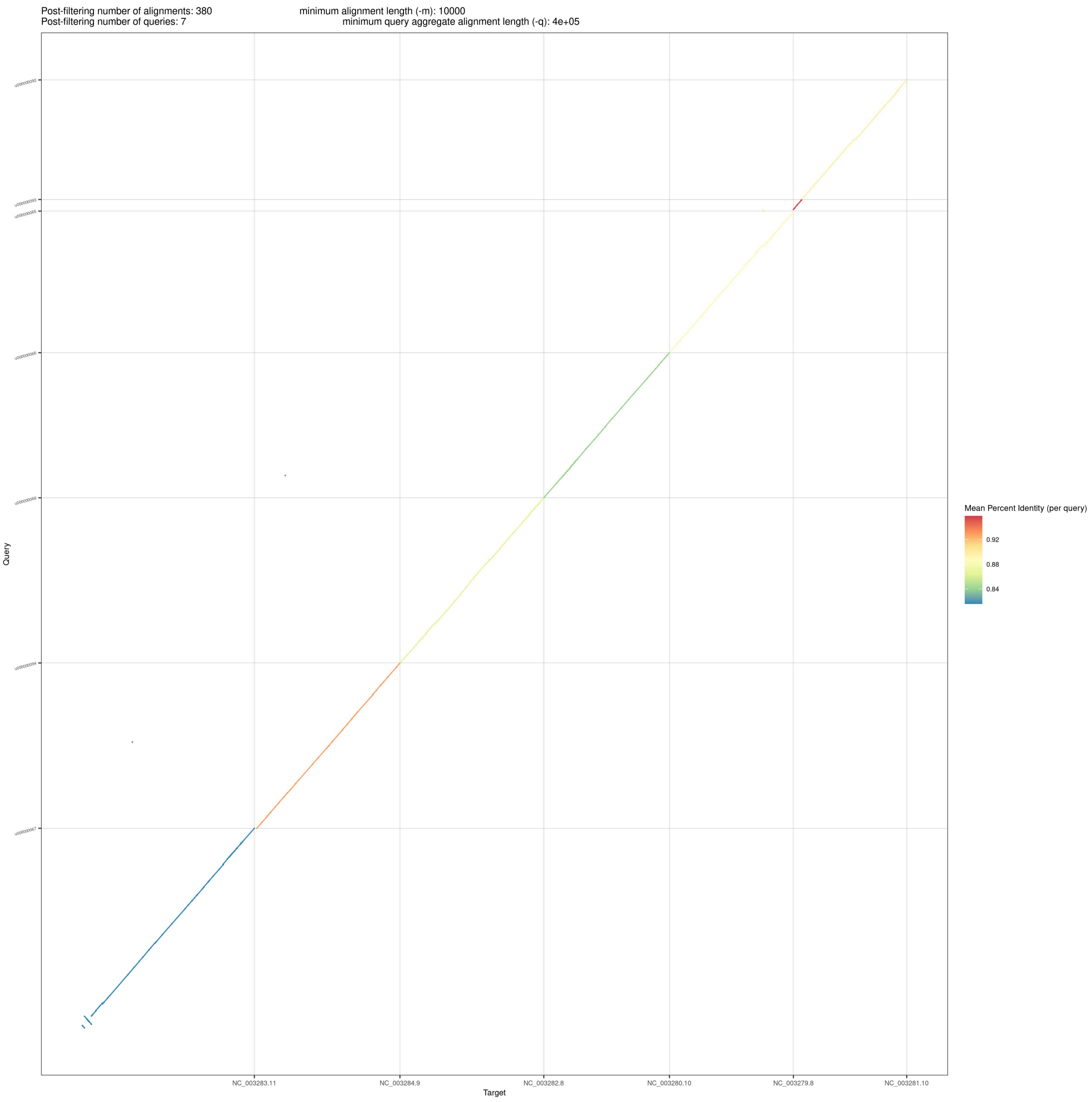

Figure S25. Dot plot of *Caenorhabditis elegans* for Pin_hic. The horizontal axis represents the T2T reference genome, and the vertical axis represents the test genome. The colors indicate the degree of identity.

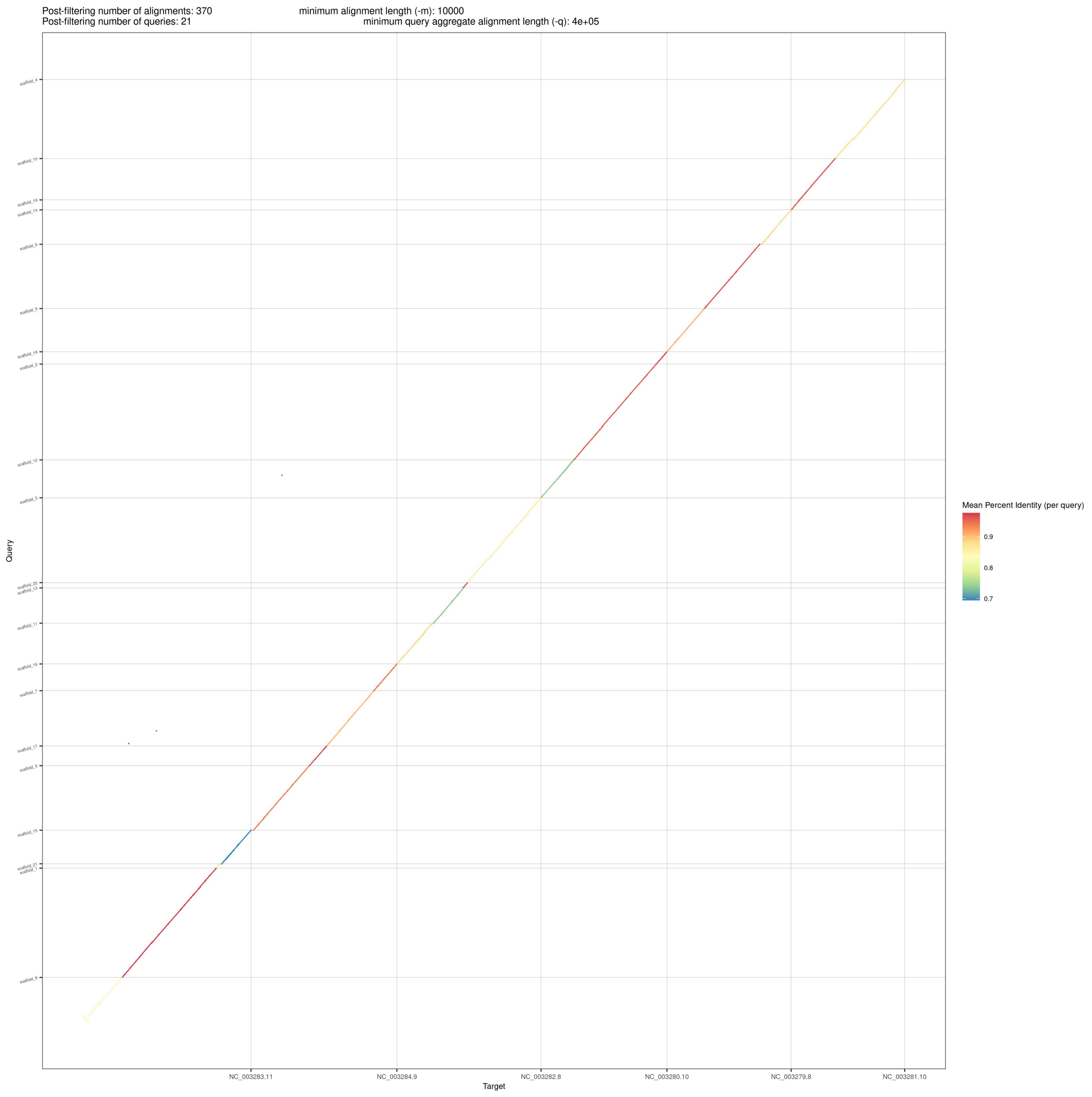

Figure S26. Dot plot of *Caenorhabditis elegans* for SALSA2. The horizontal axis represents the T2T reference genome, and the vertical axis represents the test genome. The colors indicate the degree of identity.

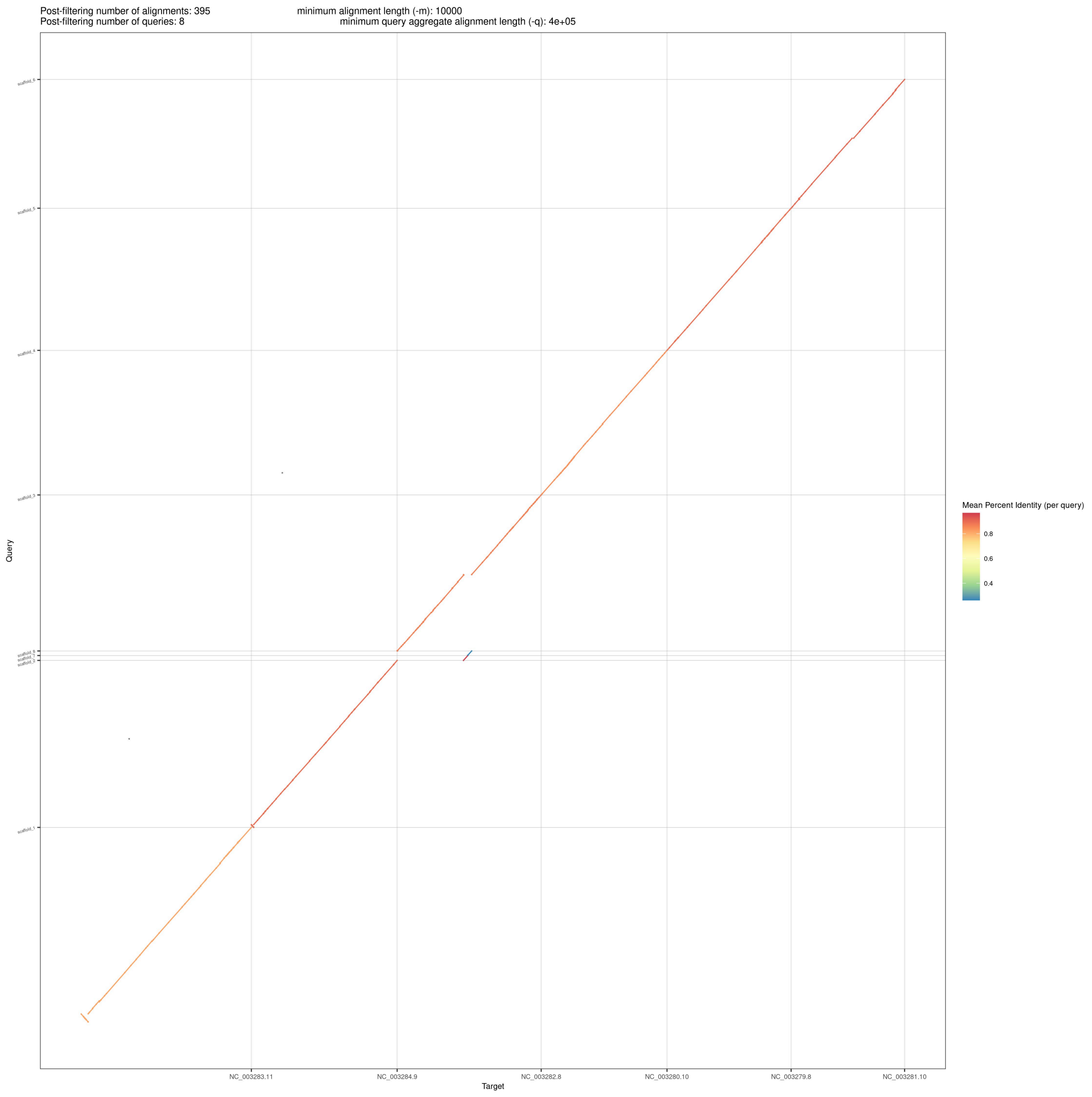

Figure S27. Dot plot of *Caenorhabditis elegans* for YaHS. The horizontal axis represents the T2T reference genome, and the vertical axis represents the test genome. The colors indicate the degree of identity.

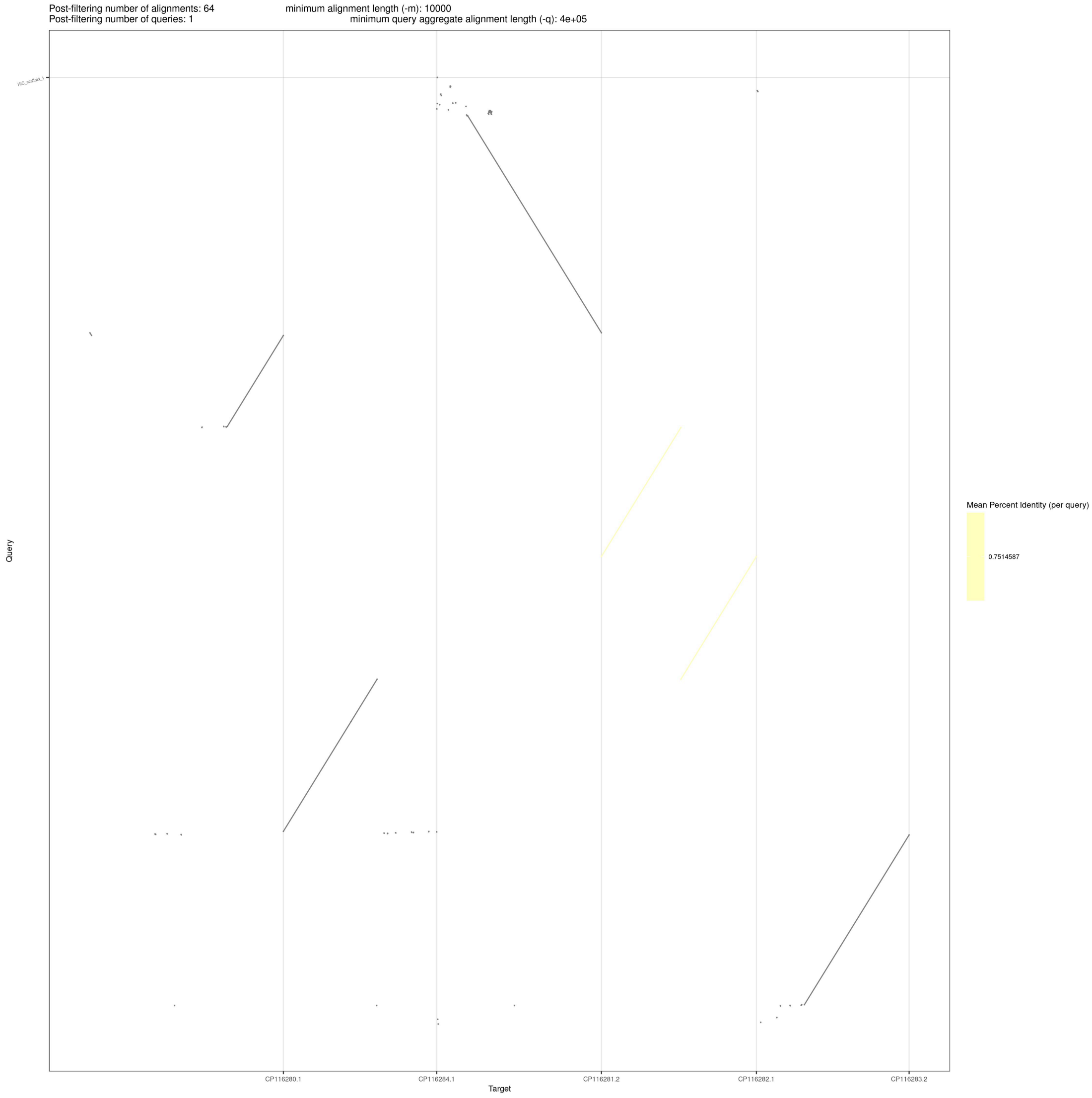

Figure S28. Dot plot of *Arabidopsis thaliana* for 3D-DNA. The horizontal axis represents the T2T reference genome, and the vertical axis represents the test genome. The colors indicate the degree of identity.

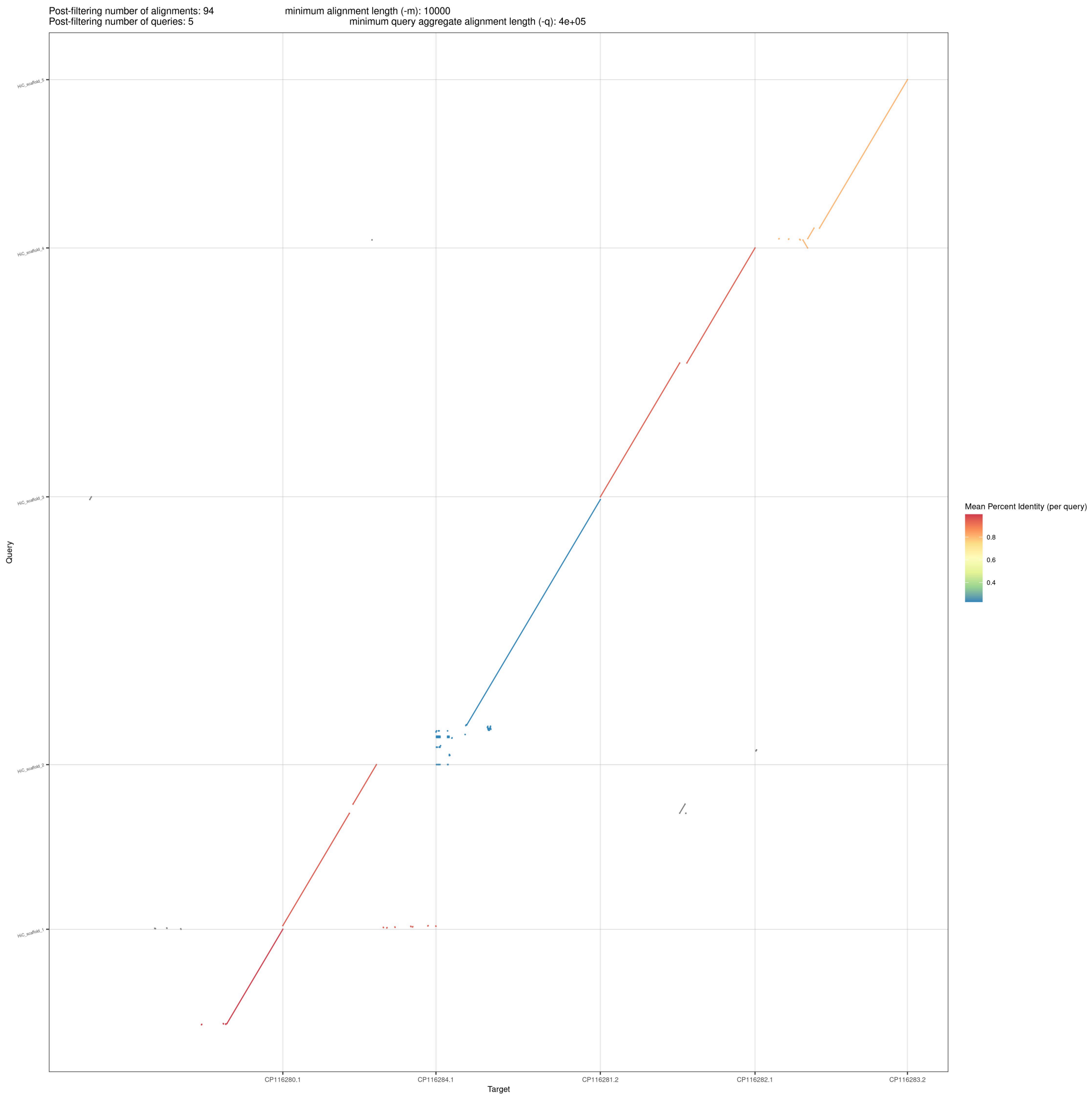

Figure S29. Dot plot of *Arabidopsis thaliana* for AutoHiC. The horizontal axis represents the T2T reference genome, and the vertical axis represents the test genome. The colors indicate the degree of identity.

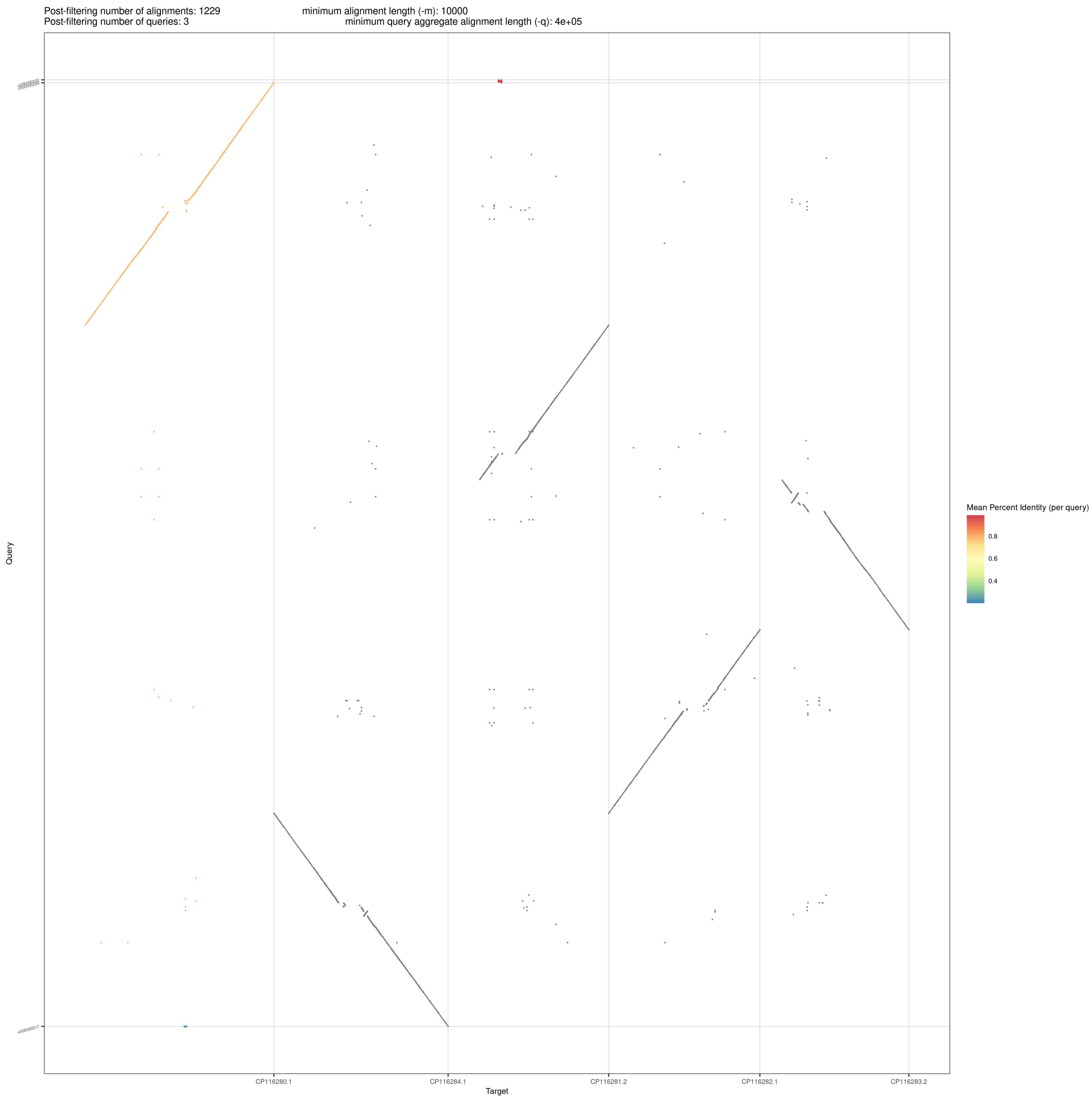

Figure S30. Dot plot of *Arabidopsis thaliana* for Pin_hic. The horizontal axis represents the T2T reference genome, and the vertical axis represents the test genome. The colors indicate the degree of identity.

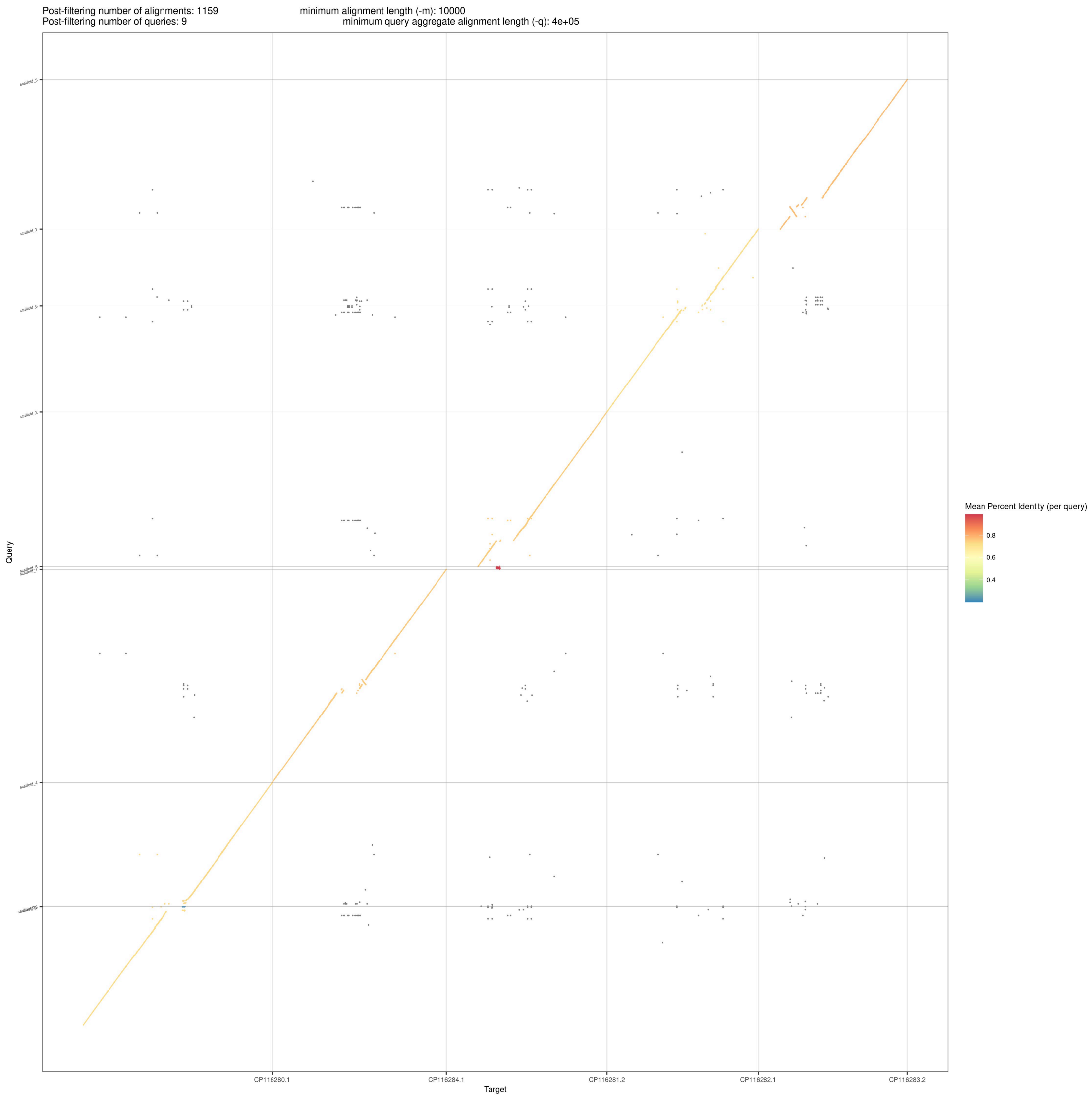

Figure S31. Dot plot of *Arabidopsis thaliana* for SALSA2. The horizontal axis represents the T2T reference genome, and the vertical axis represents the test genome. The colors indicate the degree of identity.

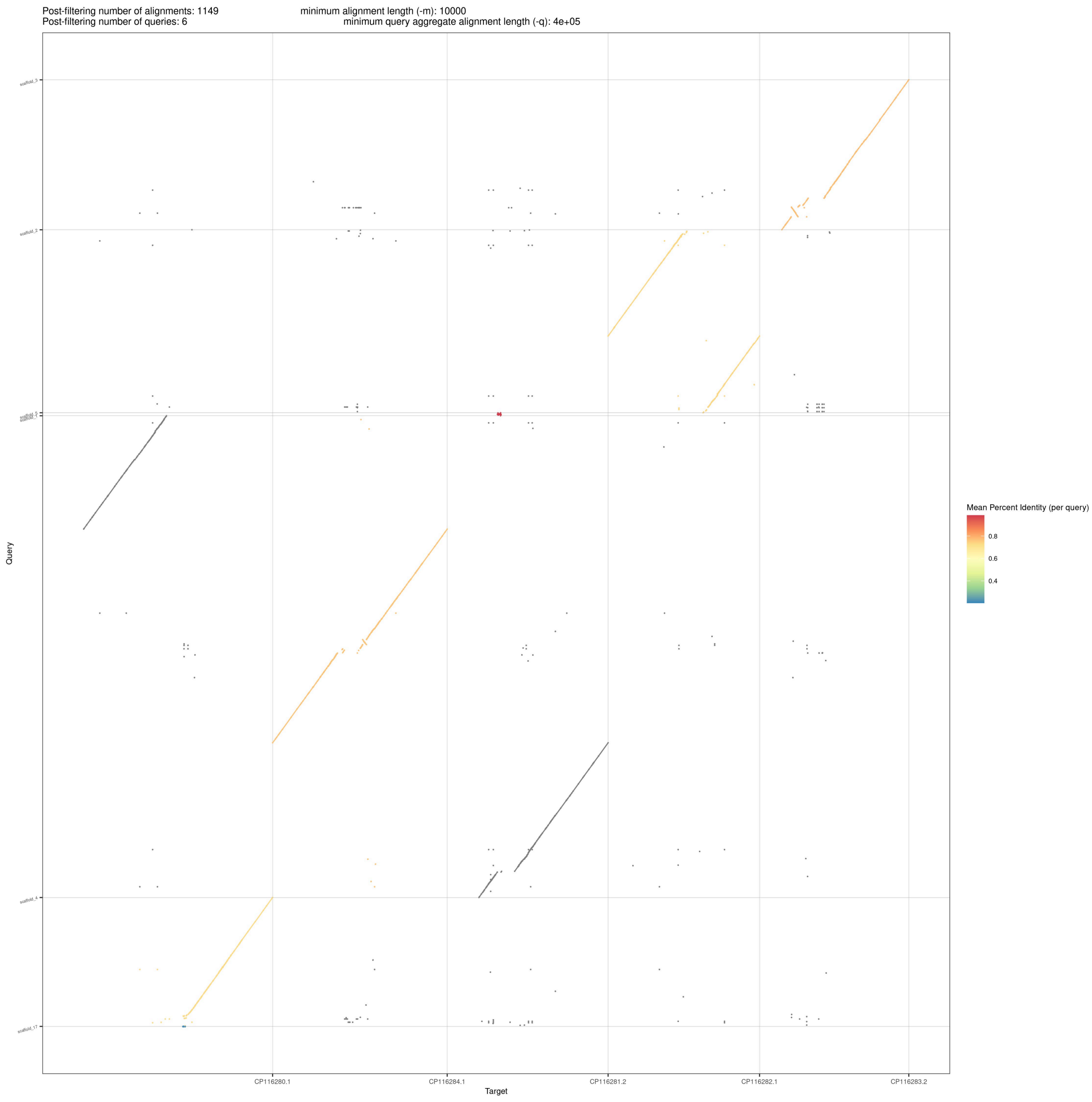

Figure S32. Dot plot of *Arabidopsis thaliana* for YaHS. The horizontal axis represents the T2T reference genome, and the vertical axis represents the test genome. The colors indicate the degree of identity.

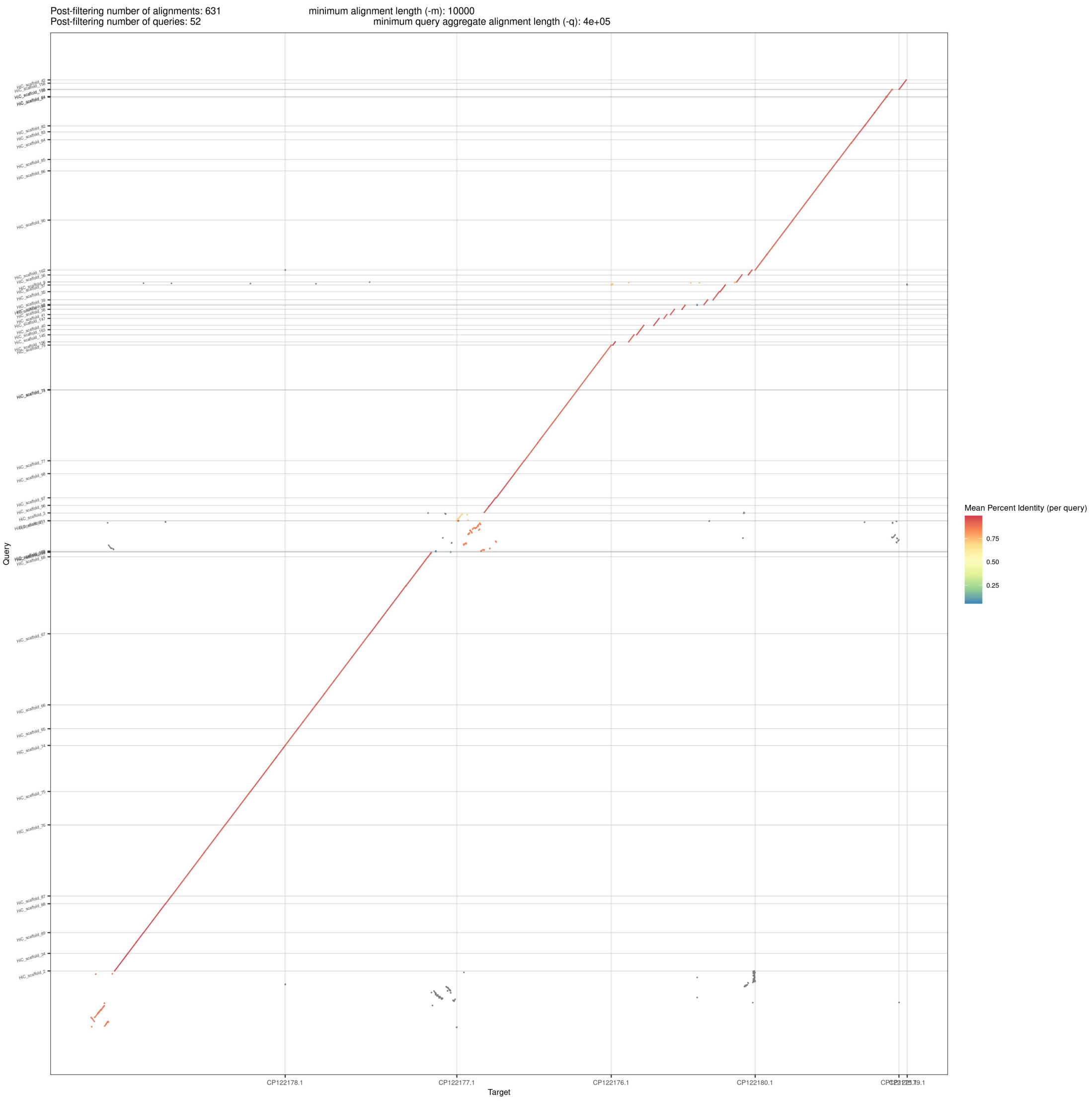

Figure S33. Dot plot of *Drosophila melanogaster* for 3D-DNA. The horizontal axis represents the T2T reference genome, and the vertical axis represents the test genome. The colors indicate the degree of identity.

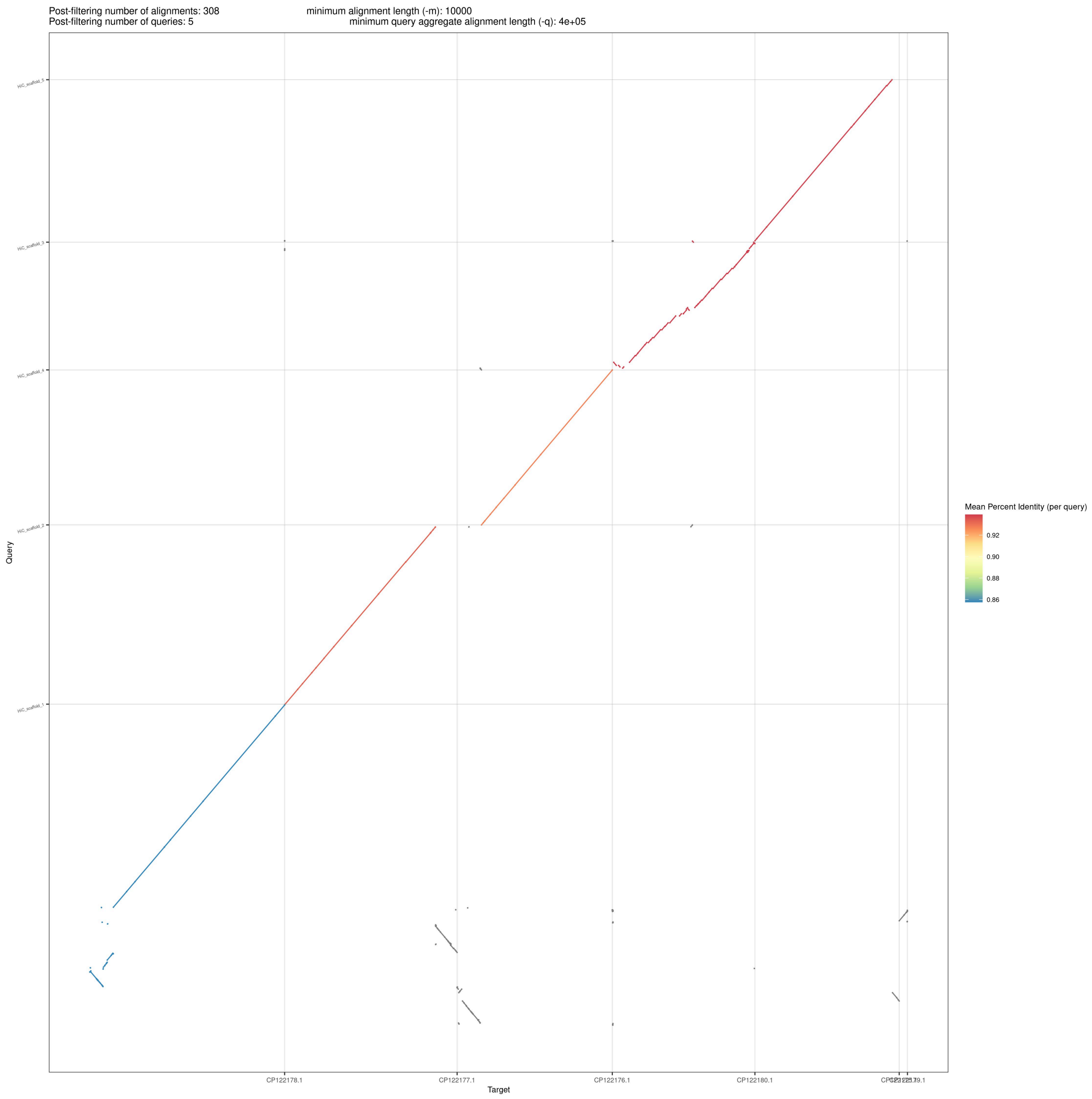

Figure S34. Dot plot of *Drosophila melanogaster* for AutoHiC. The horizontal axis represents the T2T reference genome, and the vertical axis represents the test genome. The colors indicate the degree of identity.

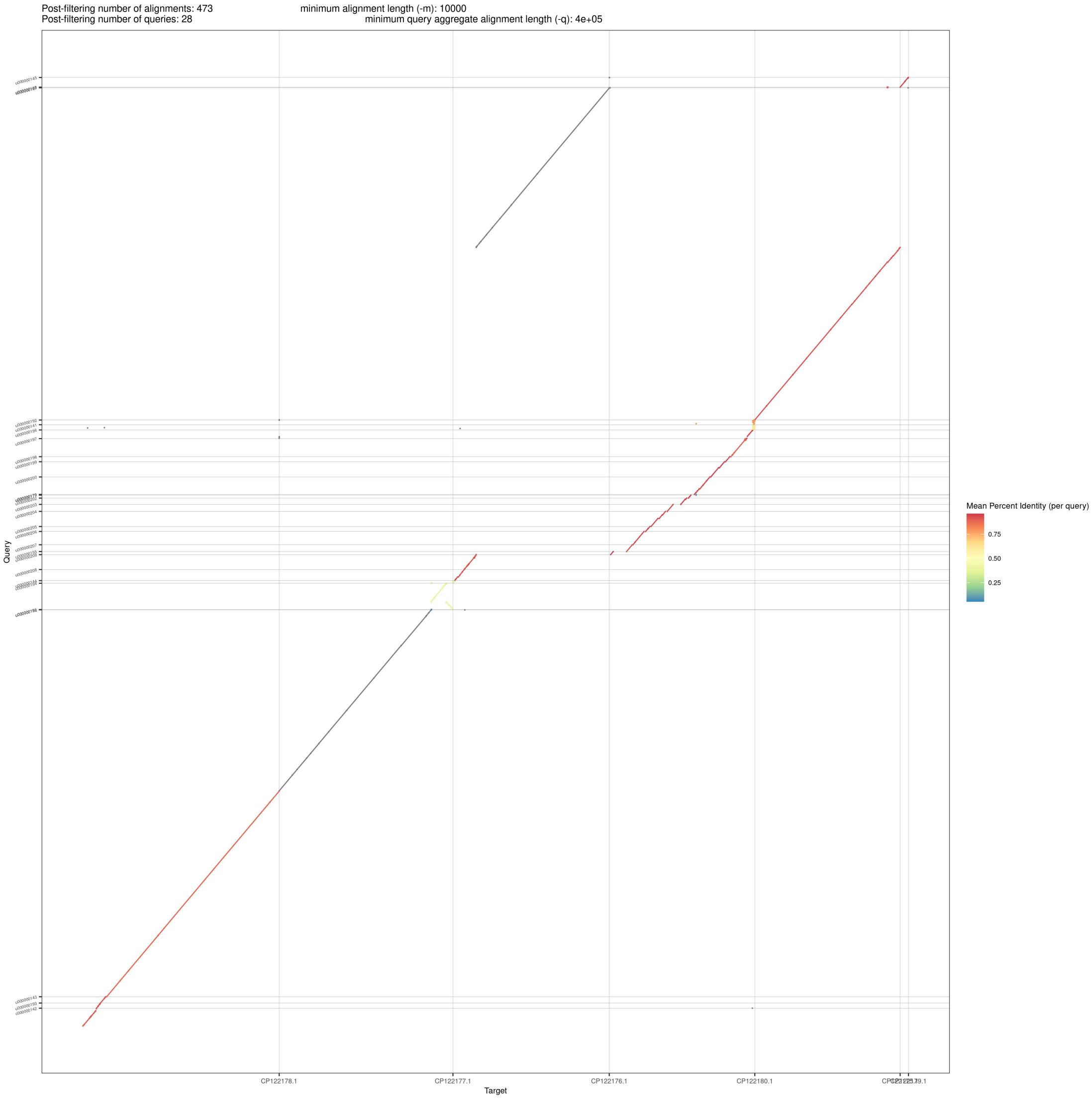

Figure S35. Dot plot of *Drosophila melanogaster* for Pin_hic. The horizontal axis represents the T2T reference genome, and the vertical axis represents the test genome. The colors indicate the degree of identity.

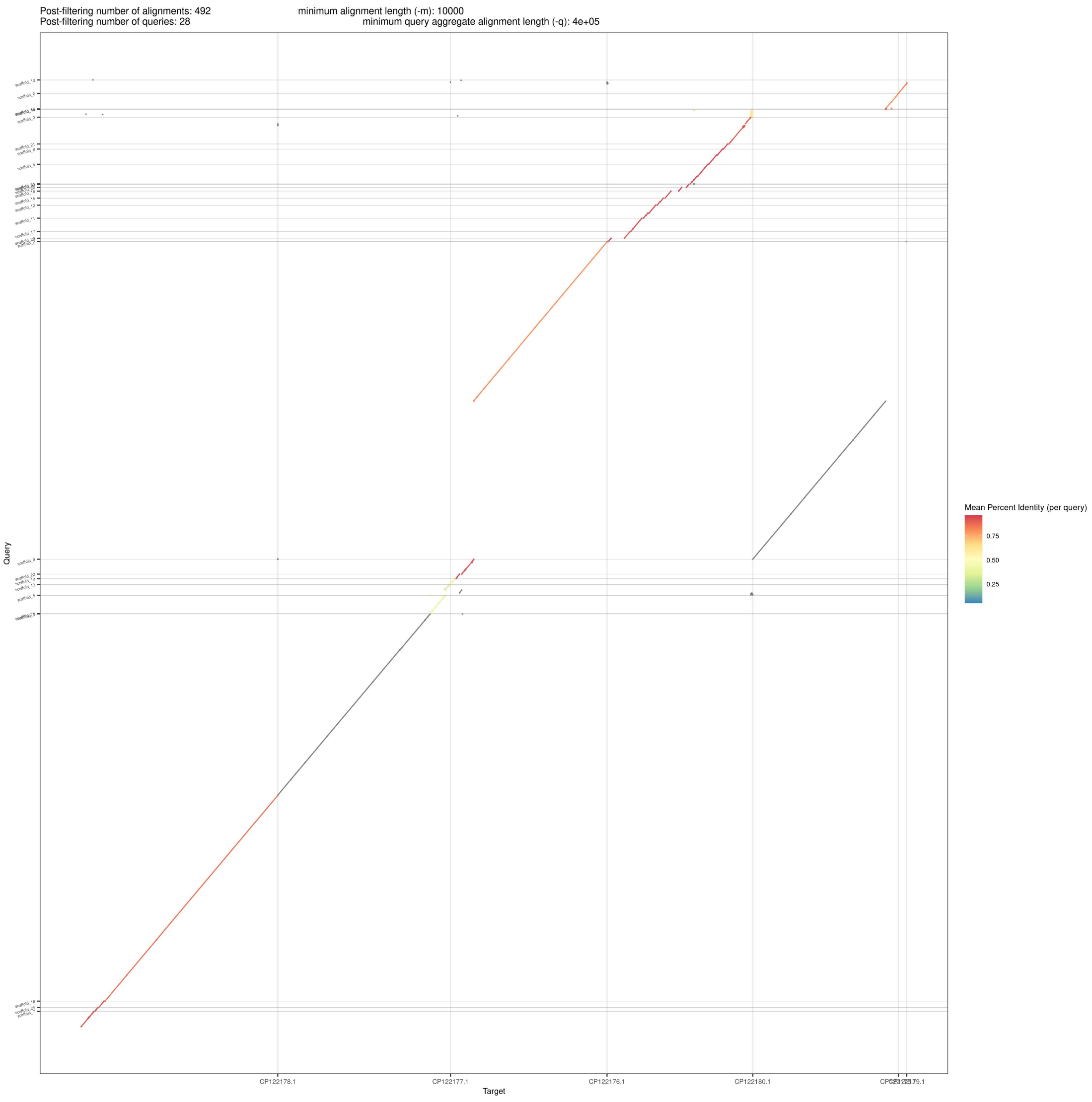

Figure S36. Dot plot of *Drosophila melanogaster* for SALSA2. The horizontal axis represents the T2T reference genome, and the vertical axis represents the test genome. The colors indicate the degree of identity.

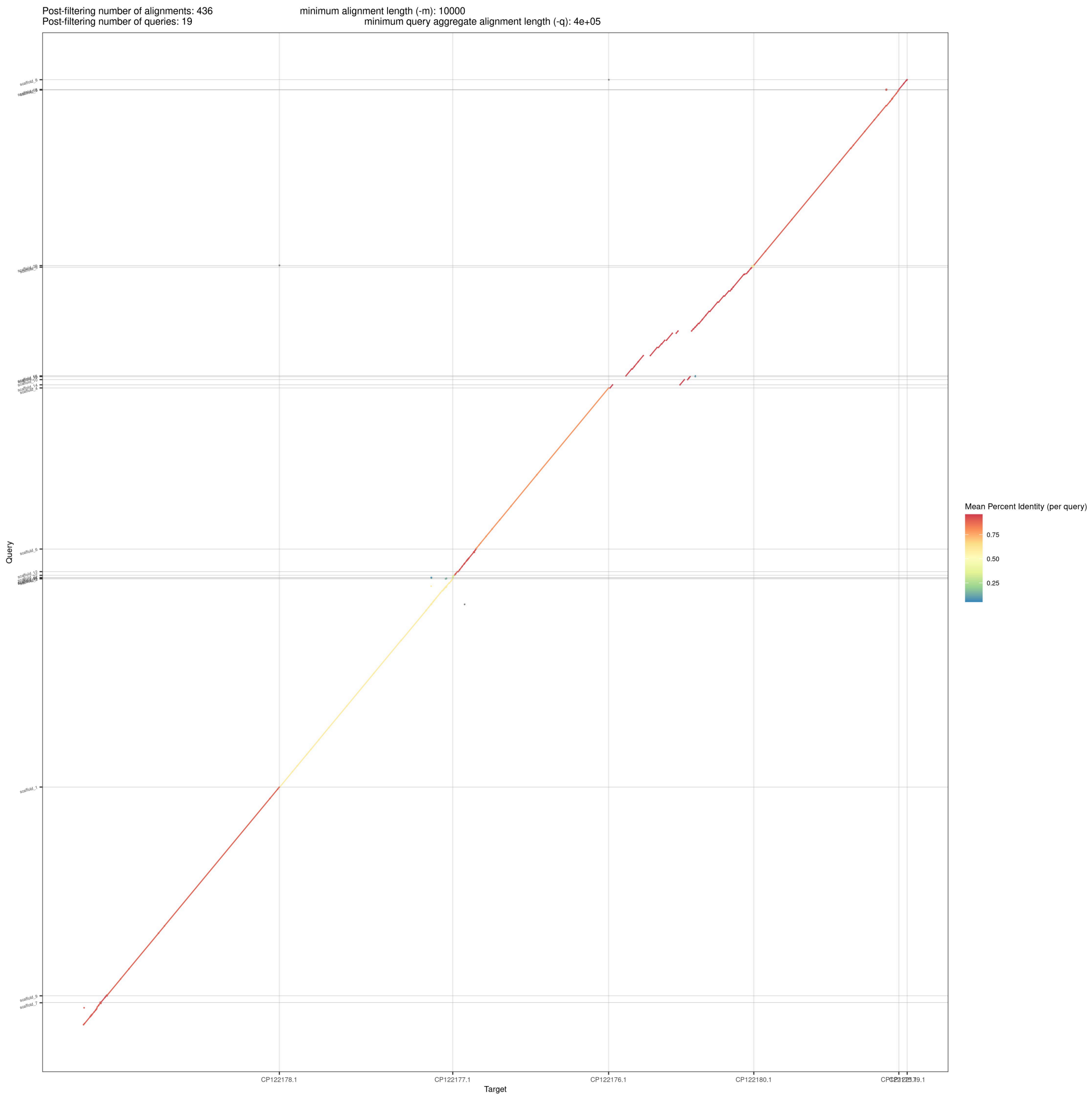

Figure S37. Dot plot of *Drosophila melanogaster* for YaHS. The horizontal axis represents the T2T reference genome, and the vertical axis represents the test genome. The colors indicate the degree of identity.

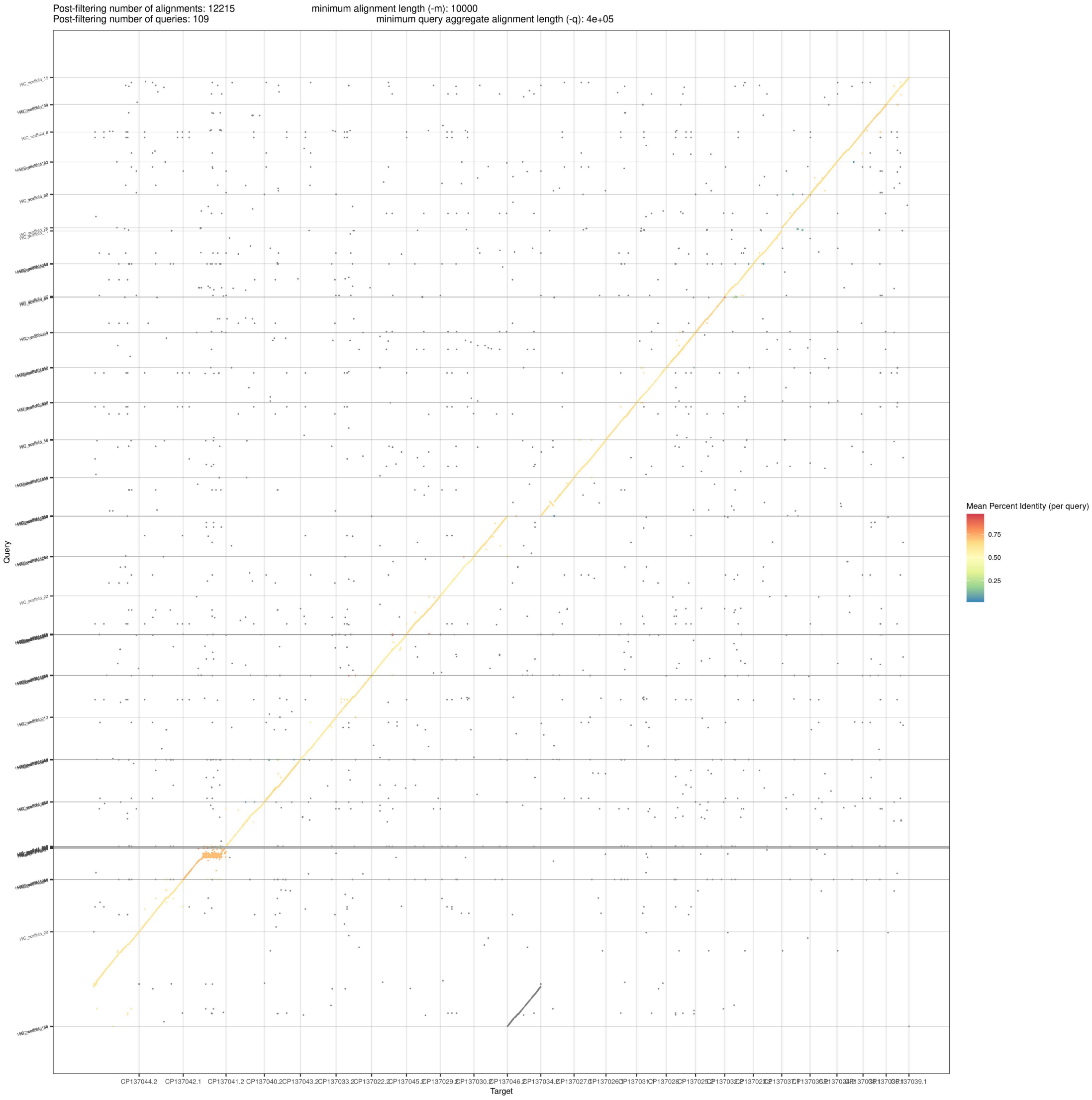

Figure S38. Dot plot of *Danio rerio* for 3D-DNA. The horizontal axis represents the T2T reference genome, and the vertical axis represents the test genome. The colors indicate the degree of identity.

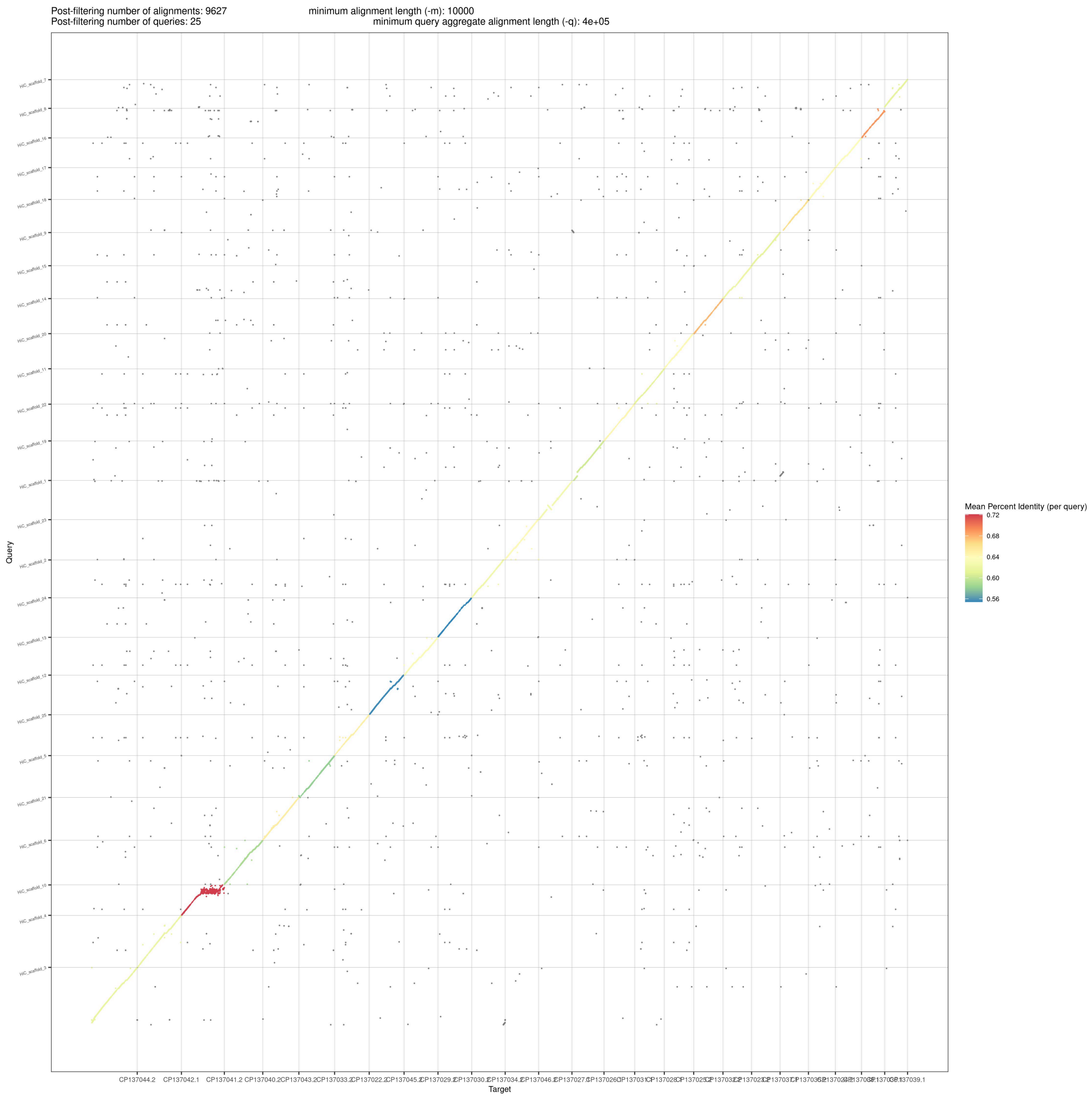

Figure S39. Dot plot of *Danio rerio* for AutoHiC. The horizontal axis represents the T2T reference genome, and the vertical axis represents the test genome. The colors indicate the degree of identity.

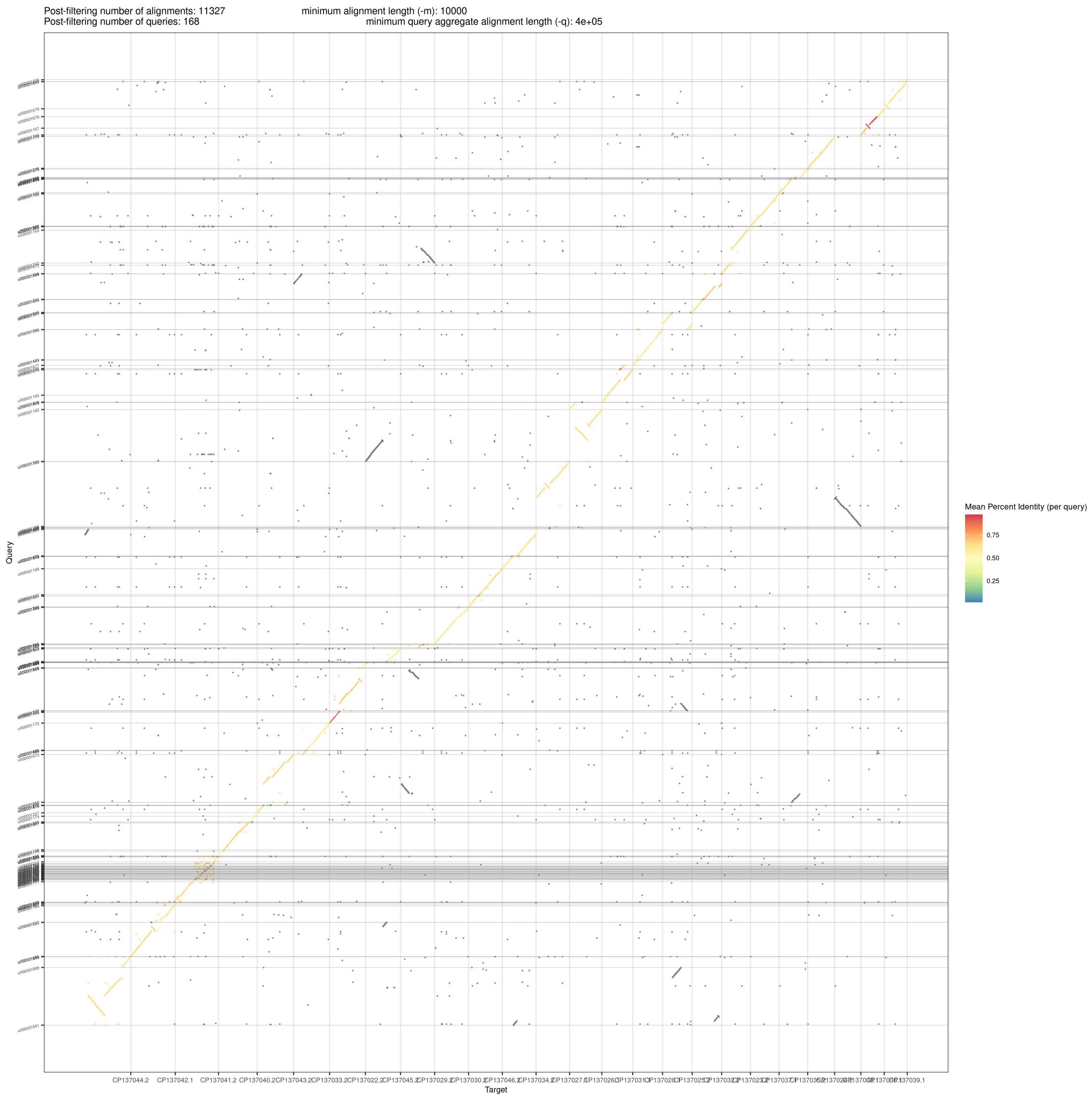

Figure S40. Dot plot of *Danio rerio* for Pin_hic. The horizontal axis represents the T2T reference genome, and the vertical axis represents the test genome. The colors indicate the degree of identity.

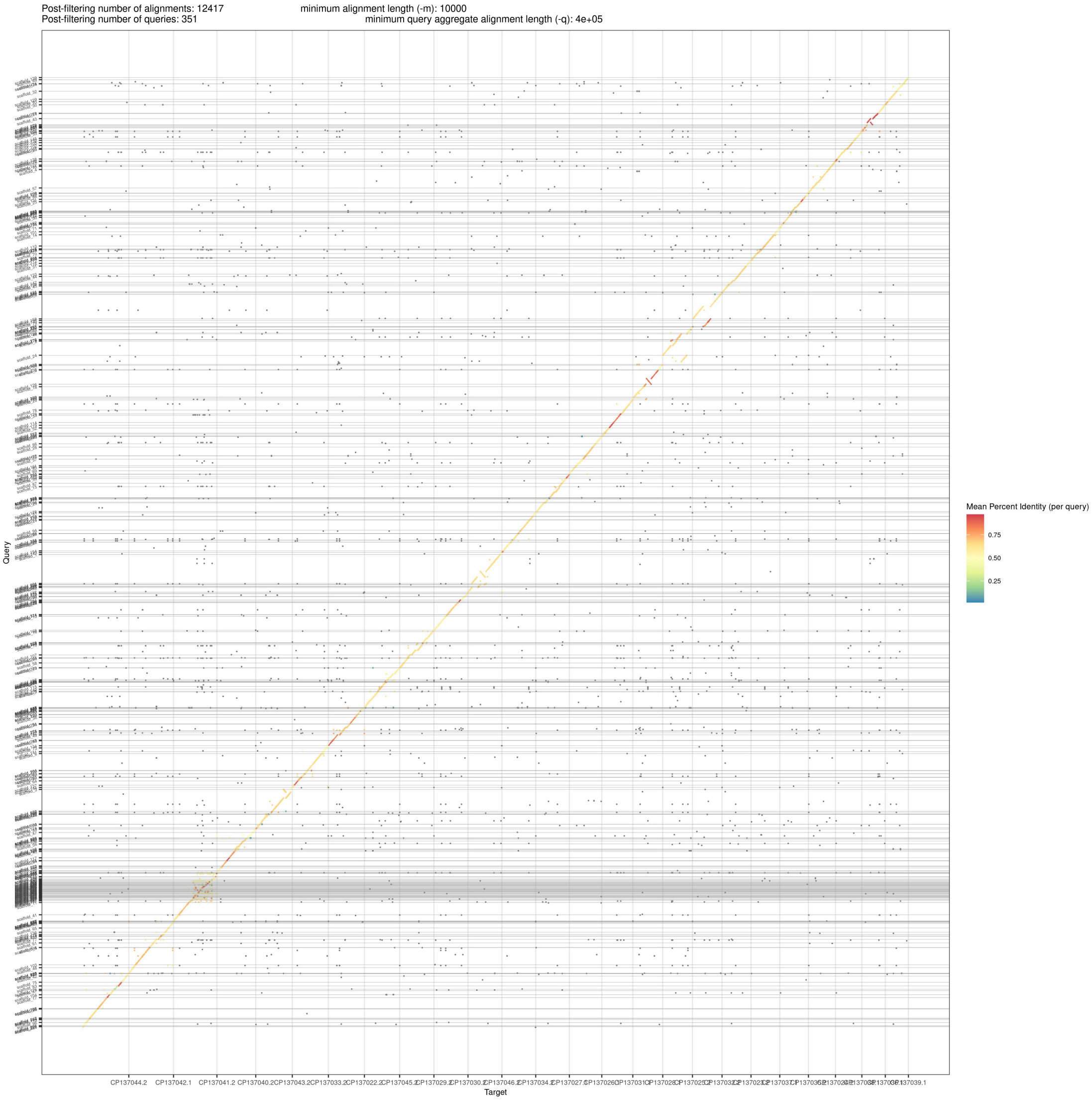

Figure S41. Dot plot of *Danio rerio* for SALSA2. The horizontal axis represents the T2T reference genome, and the vertical axis represents the test genome. The colors indicate the degree of identity.

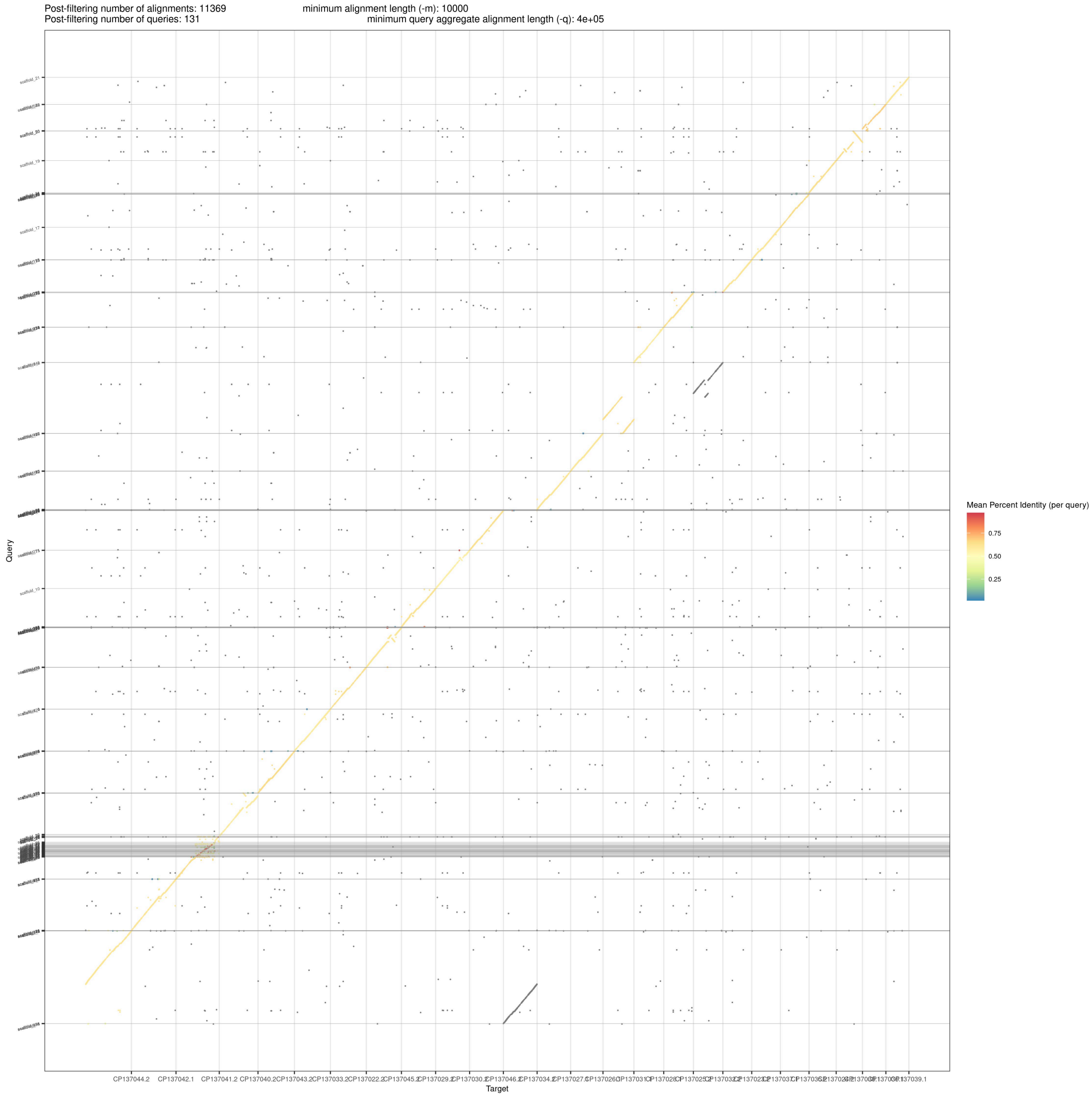

Figure S42. Dot plot of *Danio rerio* for YaHS. The horizontal axis represents the T2T reference genome, and the vertical axis represents the test genome. The colors indicate the degree of identity.

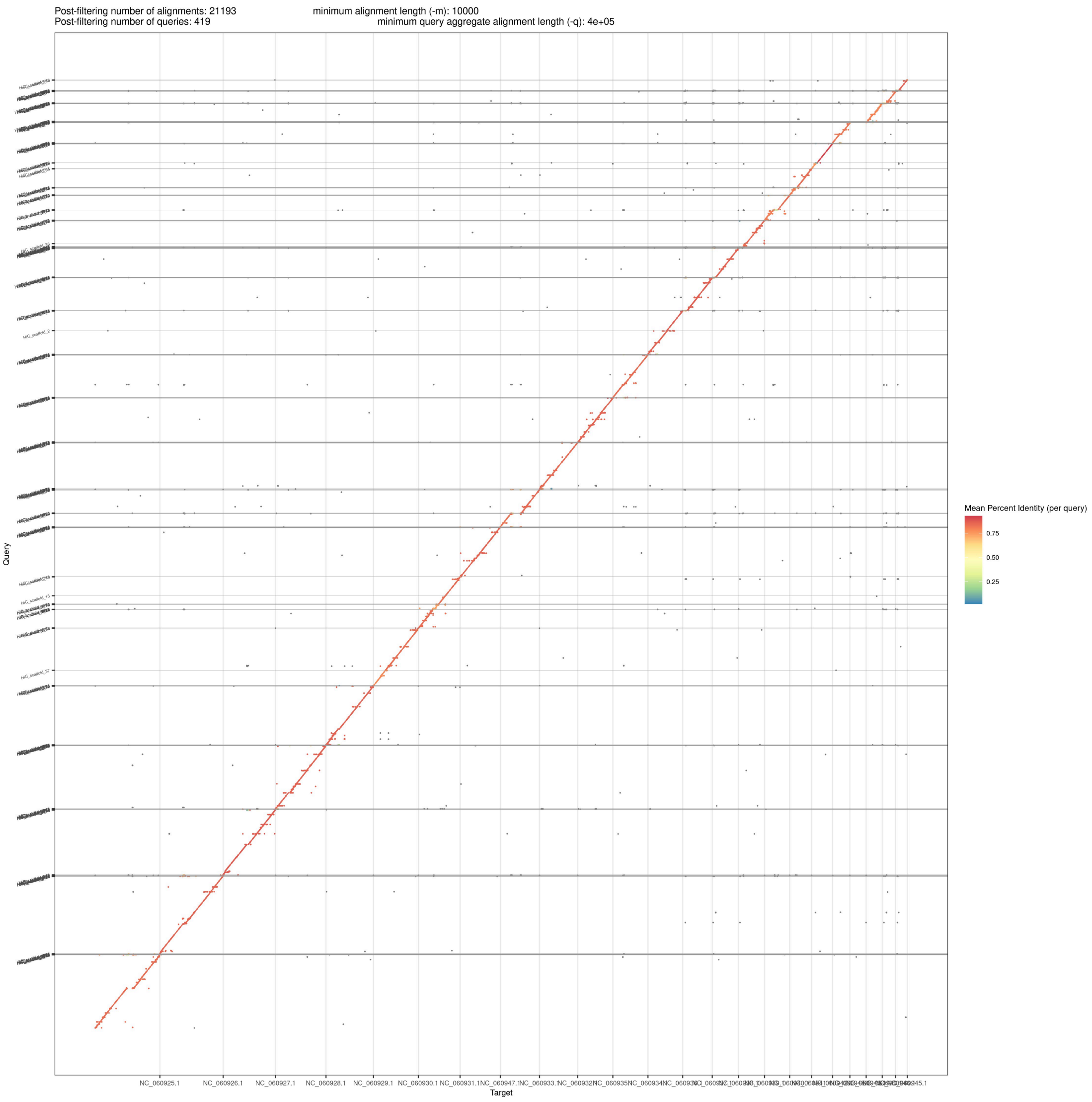

Figure S43. Dot plot of *Homo sapiens* for 3D-DNA. The horizontal axis represents the T2T reference genome, and the vertical axis represents the test genome. The colors indicate the degree of identity.

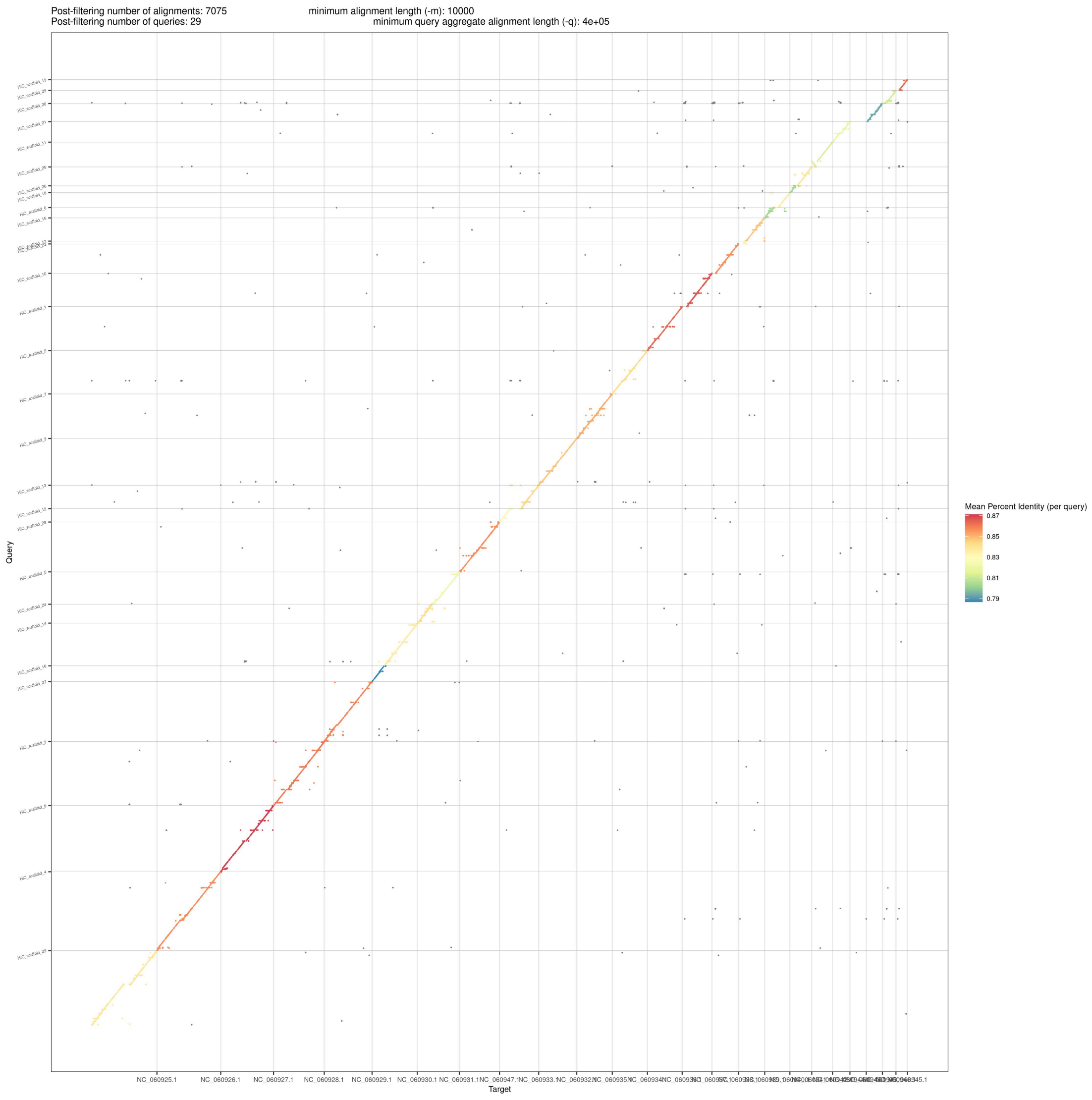

Figure S44. Dot plot of *Homo sapiens elegans* for AutoHiC. The horizontal axis represents the T2T reference genome, and the vertical axis represents the test genome. The colors indicate the degree of identity.

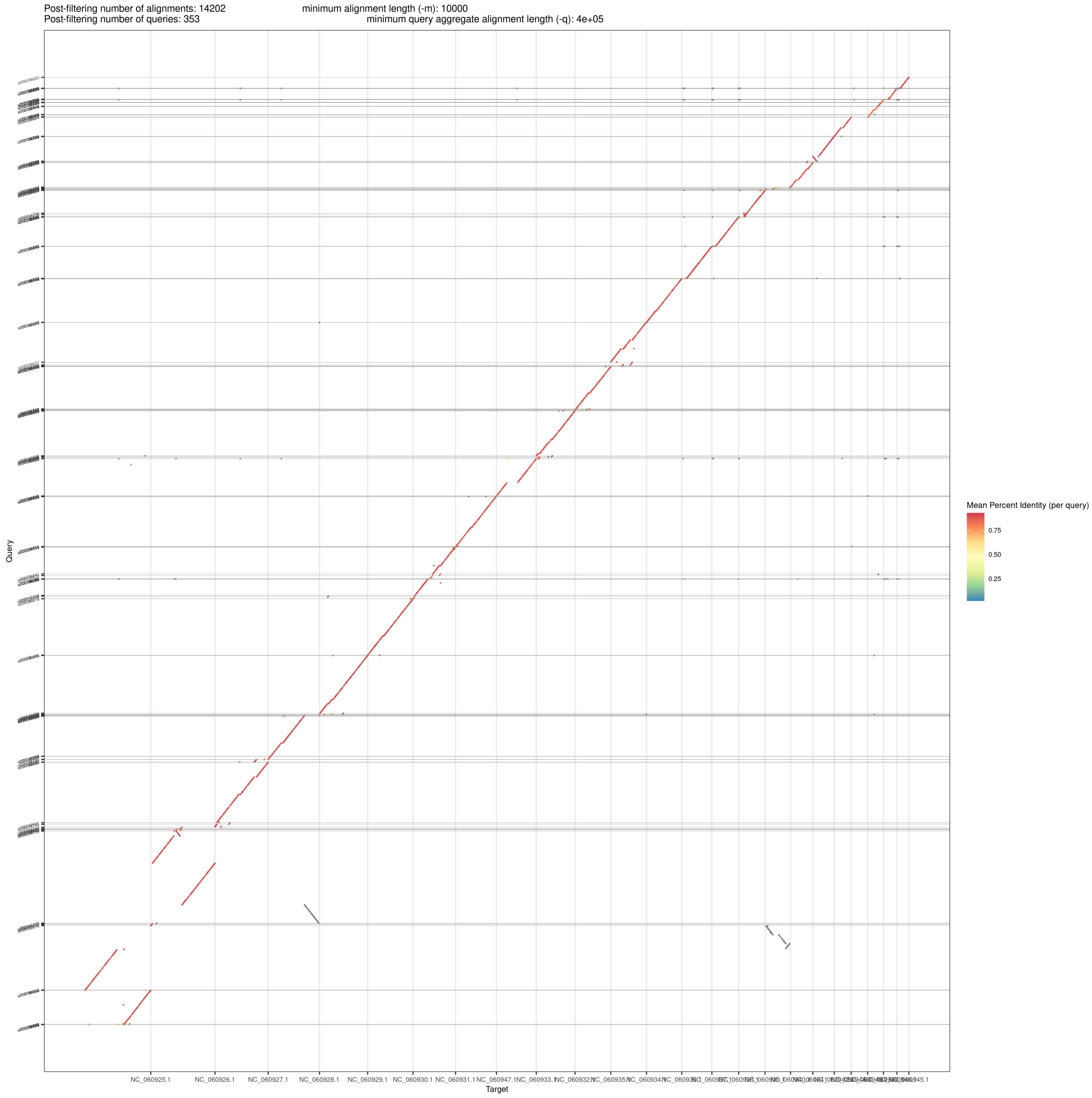

Figure S45. Dot plot of *Homo sapiens* for Pin_hic. The horizontal axis represents the T2T reference genome, and the vertical axis represents the test genome. The colors indicate the degree of identity.

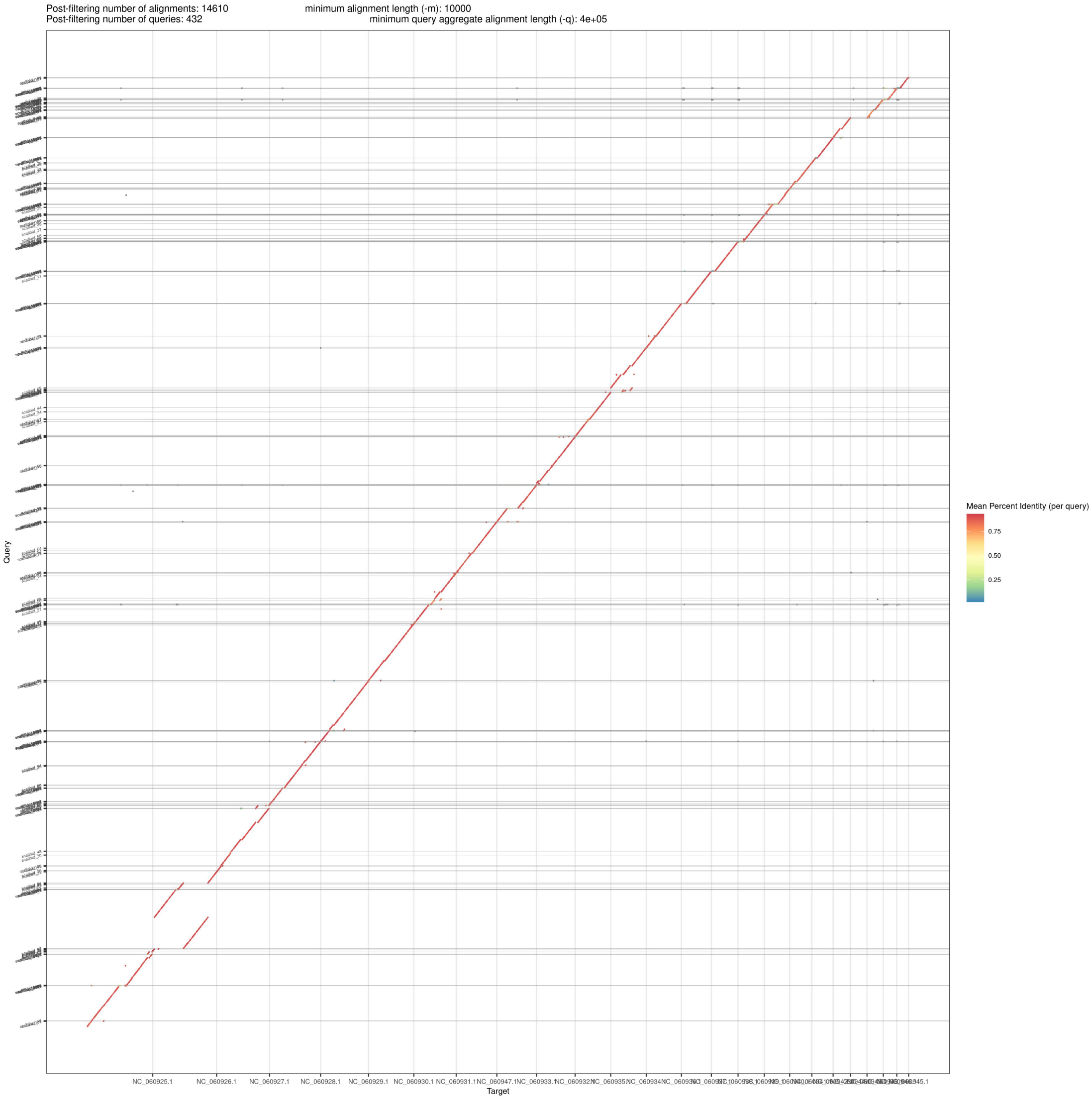

Figure S46. Dot plot of *Homo sapiens* for SALSA2. The horizontal axis represents the T2T reference genome, and the vertical axis represents the test genome. The colors indicate the degree of identity.

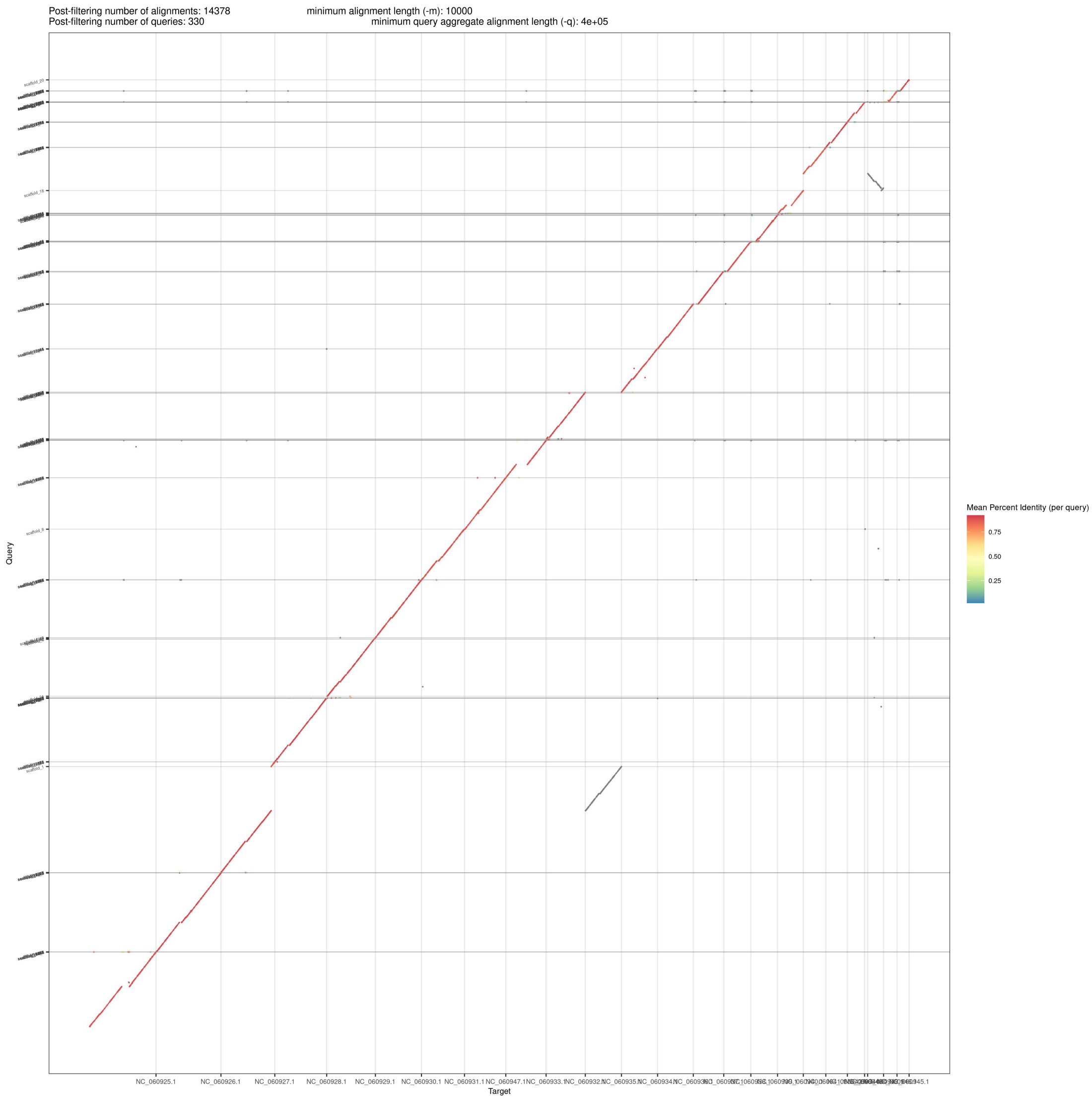

Figure S47. Dot plot of *Homo sapiens* for YaHS. The horizontal axis represents the T2T reference genome, and the vertical axis represents the test genome. The colors indicate the degree of identity.

Supplementary Tables

Table S1. Scaffolding software statistics.

Name	Publication Date	Current version	Link
Lachesis	December 1, 2013		https://github.com/shendurelab/LACHESIS
3D-DNA	Mar 23, 2017	201008	https://github.com/aidenlab/3d-dna
SALSA2	August 21, 2019	2.3	https://github.com/marbl/SALSA
yahs	December 16, 2022	1.1	https://github.com/c-zhou/yahs
instaGRAAL	June 1, 2020	0.1.6	https://github.com/koszullab/instaGRAAL
EndHiC	December 8, 2022	1.0.0	https://github.com/fanagislab/EndHiC
Pin_hic	December 31, 2021	3.0.0	https://github.com/dfguan/pin_hic
HiRise	March 26, 2016		https://github.com/fogleman/HiRISE
AllHiC	August 5, 2019	0.9.8	https://github.com/tangerzhang/ALLHiC
SALSA1	July 12, 2017	2.3	https://github.com/marbl/SALSA

Table S2. QUAST results compared with the software assembly results.

Please see separate table file (Supplementary Tables).

Table S3. Nx index statistics for comparing software assembly results.

Please see separate table file (Supplementary Tables).

Table S4. Assembly result sequence length statistics.

Please see separate table file (Supplementary Tables).

Table S5. QUAST results of the T2T assembly.

Please see separate table file (Supplementary Tables).

Table S6. *Homo sapiens* MUM&Co results before correcting errors.

Please see separate table file (Supplementary Tables).

Table S7. *Homo sapiens* MUM&Co results after correcting the errors.

Please see separate table file (Supplementary Tables).

Table S8. QUAST results on the extended test dataset.

Please see separate table file (Supplementary Tables).

Table S9. Genome data and Hi-C raw data statistics.

Please see separate table file (Supplementary Tables).

Table S10. Model training dataset statistics

Please see separate table file (Supplementary Tables).

Table S11. AutoHiC runtime.

Please see separate table file (Supplementary Tables).

Table S12. Genome size statistics.

Please see separate table file (Supplementary Tables).

References

1. D. Guan, *et al.*, Efficient iterative Hi-C scaffolder based on N-best neighbors. *BMC Bioinformatics* 22, 569 (2021).

2. C. Zhou, S. A. McCarthy, R. Durbin, YaHS: yet another Hi-C scaffolding tool. *Bioinformatics* 39, btac808 (2023).

3. K. Li, P. Xu, J. Wang, X. Yi, Y. Jiao, Identification of errors in draft genome assemblies at single-nucleotide resolution for quality assessment and improvement. *Nat. Commun.* 14, 6556 (2023).

4. O. Dudchenko, *et al.*, De novo assembly of the Aedes aegypti genome using Hi-C yields chromosome-length scaffolds. *Science* 356, 92–95 (2017).

5. O. Dudchenko, *et al.*, The Juicebox Assembly Tools module facilitates de novo assembly of mammalian genomes with chromosome-length scaffolds for under $1000. [Preprint] (2018). Available at: https://www.biorxiv.org/content/10.1101/254797v1 [Accessed 29 January 2024].
